# Prognosis prediction and immune microenvironment features of breast cancer indicated by a cuproptosis-associated long non-coding RNA signature

**DOI:** 10.1016/j.gendis.2023.101110

**Published:** 2023-09-22

**Authors:** Siyi Liu, Juanchan Huang, Wei Zhao

**Affiliations:** a Department of Radiation Oncology, Guangxi Medical University Cancer Hospital, Nanning, Guangxi 530000, China; b Guangxi Medical University, Nanning, Guangxi 530000, China; c Department of Oncology, Affiliated Wuming Hospital of Guangxi Medical University, Nanning, Guangxi 530000, China

Cuproptosis is a newly discovered type of programmed cell death that involves the depletion of intracellular copper and is not influenced by other inhibitors of programmed cell death. This modality was first identified and named by Tsvetkov et al in the previous year.^1^ To further explore the relationship between cuproptosis, the tumor microenvironment, immunotherapy, and prognosis for breast cancer (BC), the expression patterns of 19 cuproptosis-related long non-coding RNAs (lncRNAs) were determined using data from the Cancer Genome Atlas (TCGA). Through Cox and Lasso regression analyses, we identified three crucial lncRNAs: AL137847.1, LRRC8C-DT, and NIFK-AS1. These lncRNAs were selected to establish a risk prediction model. Additionally, a nomogram was constructed by combining the clinical characteristics with the developed model. Differential expression analysis and functional enrichment analysis were performed based on the high- and low-risk groups derived from the risk prediction model. In addition, the mutant landscape of lncRNAs in the TCGA cohort was investigated, and the correlation between tumor mutational burden (TMB), immune activation pathways, and the prognostic model was analyzed. Real-time quantitative PCR experiments confirmed that the expression levels of AL137847.1, LRRC8C-DT, and NIFK-AS1 were significantly higher in MDA-MB-231 breast cancer cells compared with normal mammary epithelial cells. Furthermore, drug sensitivity analyses were carried out. The findings of this study may serve as a reference for individualized prognosis prediction and immunotherapy strategies for BC patients.

The transcriptome data of lncRNAs and clinical data from 1113 BC patients and 113 normal samples were obtained from the TCGA database. The samples were selected based on specific criteria: (i) histological diagnosis of breast cancer, (ii) availability of gene expression data, and (iii) data retrievability. Patients with duplicate enrollments were excluded from the dataset. The present study included a Sankey map (Fig. S1) illustrating the co-expression relationships between the lncRNAs associated with breast cancer and 16 cuproptosis-related genes. From this analysis, seven cuproptosis-related lncRNAs with potential prognostic significance were identified through univariate Cox analysis. Subsequently, multivariate Cox regression and Lasso regression were performed to narrow down the selection to the three central prognostic-related lncRNAs: AL137847.1, LRRC8CDT, and NIFKAS1. These lncRNAs were used to establish the risk prediction model (Fig. S1). The risk scores and survival states of different risk groups display in Figure S2. Survival curves demonstrated that patients in the high-risk group had worse overall survival and progression-free survival over 10 years compared with those in the low-risk group (Fig. 1A–D). Independent prognostic analysis, as well as the ROC curve and C-index curve, verified that our risk prediction model could serve as an independent prognostic indicator with good prognostic ability to predict outcomes for BC patients (Fig. 1E–G). To facilitate clinical application, a nomogram was constructed by combining the clinical characteristics with the established risk prediction model. The calibration curves for 1-year, 3-year, and 5-year overall survival probability demonstrated a high correlation between the predicted survival and the actual survival (Fig. 1H, I). A subgroup analysis based on clinical factors indicated that women over 40 years old with any clinical stage and TNM stage could effectively utilize the risk score model with good predictive accuracy, presented in Figure S3. Principal component analysis showed that all genes, cuproptosis-associated lncRNAs, CRGs, and lncRNAs included in the model could effectively differentiate between high- and low-risk populations (Fig. S4). Gene Ontology and Kyoto Encyclopedia of Genes and Genomes enrichment analyses were conducted to further evaluate the relevant functions and pathways in the high- and low-risk groups (Fig. S4). Notably, cuproptosis-related lncRNAs were found to be involved in various biological functions associated with BC, including epithelial cell proliferation, muscle system process, leukocyte migration, signal receptor activation activity, receptor ligand activity, DNA-binding transcription activator activity, RNA polymerase II specific DNA-binding transcription activator activity, and neuroactive ligand–receptor interactions. Additionally, these lncRNAs were related to the composition of the extracellular matrix and collagen in BC. To assess genetic variation among high-risk and low-risk groups in the TCGA cohort, the top 15 genes with the highest frequency of change were screened, and the TMB scores of the different groups were compared (Fig. S5). The results indicated that the TMB of the high-risk group was significantly higher than that of the low-risk group. In addition, survival analysis incorporating both TMB and risk scores demonstrated that the risk level was a robust prognostic factor. Specifically, the high-TMB/high-risk group exhibited the worst prognosis. To investigate differences in immune pathways between risk groups, immune pathway heat maps were generated. The heat maps revealed significant distinctions in type II interferon response and antigen-presenting cell co-stimulation between the two risk groups (Fig. S5). Furthermore, cuproptosis-related lncRNAs were used to predict drug sensitivity in high- and low-risk patients. It was discovered that the sensitivity to 77 medications varied significantly between the high-risk and low-risk categories (Fig. S6). Notably, patients in the low-risk group showed greater susceptibility to drugs such as AS605240, CP724714, FH535, GW−2580, MS−275, YM155, and pyrimethamine. On the other hand, drugs like A−770041, AC220, AP−24534, AS601245, BAY 61−3606, BI−2536, BMS−509744, BMS345541, BX−912, CAL−101, CGP−082996, CGP−60474, CMK, DMOG, FMK, FR−180204, GSK107091 6, GSK1904529A, GW843682X, IPA−3, JW−7−52−1, KIN001−102, KIN001−135, NG−25, NPK76−II−72−1, NSC−87877, OSU−03012, QL− XII−47, PHA−665752, PF−562271, S-triphenylmethyl-l-cysteine, TAE684, TGX221, TL−2−105, VX−11e, VX−680, WZ−1−84, XMD8−85, XMD14−99, X L−184, Z−LLNle−CHO, ZSTK474, ebomycin B, obachra mesulfonate, siterpene lactone, besalodin, pheneformin, bleomycin, dasatinib, toxic carotene, anthracene, fluorouracil, nuclear inhibitor, cyclopamine, gemcitabine, Genentech Cpd 10, lapatinib, rapamycin, lincetinib, rusotinib, middotolin, pazopanib, zipotentam, secatinib, sunitinib, bryostatin 1, tipirfanil, ispin mesulfonate, shiverin, and paclitaxel showed greater affinity in the high-risk group. Additionally, real-time quantitative PCR analysis was conducted to evaluate the differential expression levels of AL137847.1, LRRC8C−DT, and NIFK−AS1 between breast cancer cells (MDA-MB-231) and normal cells. The expression levels of these lncRNAs were significantly higher in MDA-MB-231 cells compared with normal mammary epithelial cells (Fig. S7).

**Figure 1:**
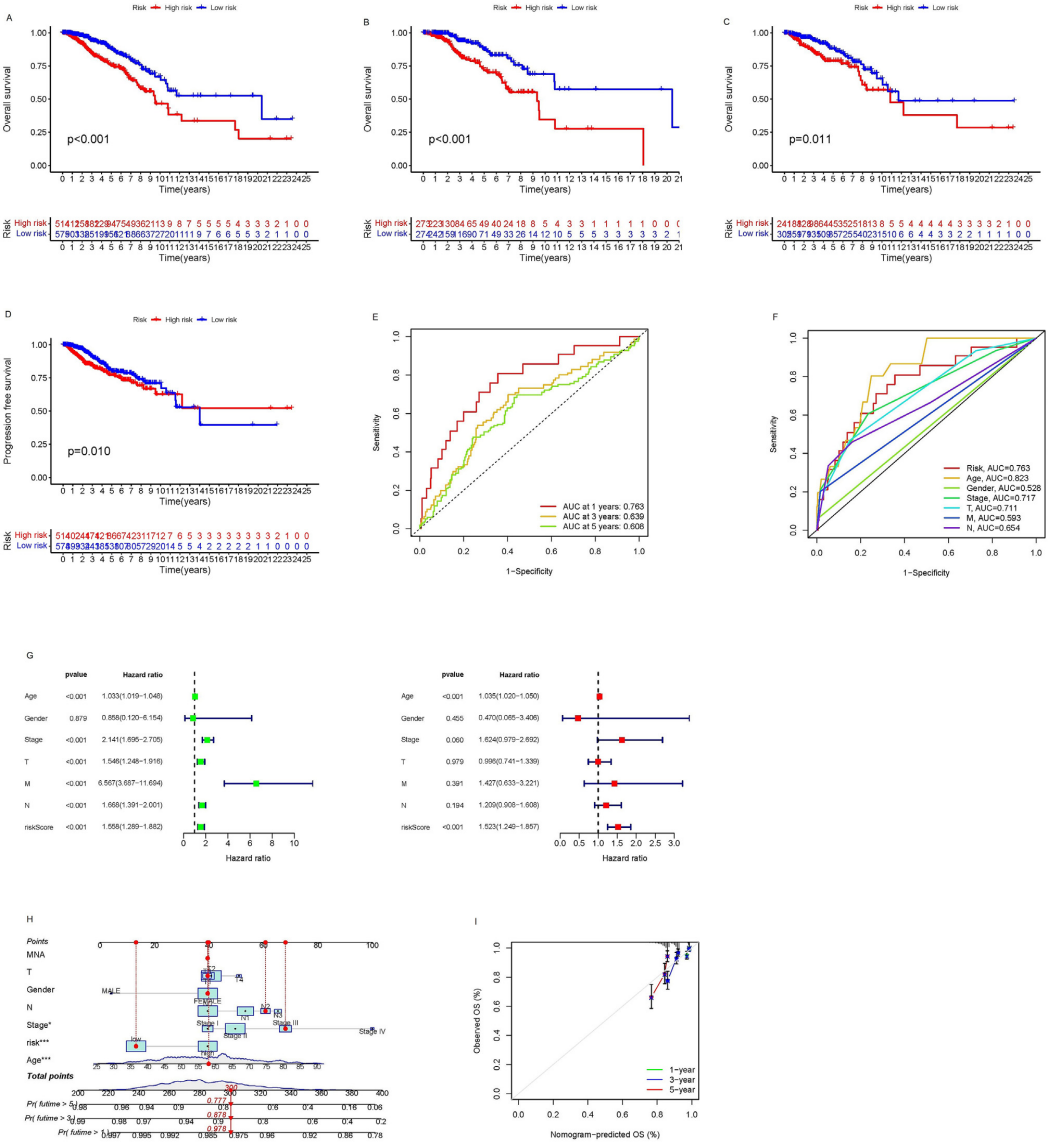
Characteristic extraction of cuproptosis-related long non-coding RNA and prognosis prediction of breast cancer. **(A)** Overall survival curve, **(B)** the training group's survival curve, and **(C)** the test group's survival curve. **(D)** Overall progression-free survival. **(E)** Receiver operator characteristic (ROC) curve of a prognostic model. **(F)** ROC curve of all risk factors. **(G)** Univariate independent prognostic analysis. **(H)** Nomogram of overall survival prognosis. **(I)** Calibrated curves of the nomogram.

This research holds several implications. Initially, the development of an algorithm for predicting BC based on cuproptosis-related lncRNAs has the potential to lead to novel therapeutic approaches for BC treatment. The study went beyond the conventional focus on overall survival alone and considered progression-free survival, risk score, immune-related function analysis, immune escape score, potential drug screening, and other variables. This comprehensive approach provides a more holistic understanding of BC prognosis compared with traditional prognostic models that solely rely on overall survival as the primary outcome. In particular, improving progression-free survival in individuals with advanced cancer can lead to reduced tumor burden and symptom relief. The division of patients into high- and low-risk groups allowed for the estimation of survival time and investigation of risk factors associated with BC. Additionally, the incorporation of the tumor immune dysfunction and exclusion score and TMB analysis can provide valuable guidance for designing immunotherapy strategies.

It is important to note some limitations of this research. Firstly, retrospective data from a public database were utilized, and the lack of prospective clinical data to validate the findings may limit the significance of the results in terms of prognosis. Secondly, since BC is a complex polygenic disease, this study only included three cuproptosis-related lncRNAs in the risk prognosis model. This approach may overlook additional lncRNAs that could potentially have a stronger predictive value for BC prognosis.

In conclusion, the incorporation of three cuproptosis-related lncRNAs (AL137847.1, LRRC8C-DT, and NIFK-AS1) in a novel risk prediction model demonstrated independent associations with both overall survival and progression-free survival. This model offers valuable insights for predicting the prognosis of BC and opens avenues for further research and clinical applications.

## Conflict of interests

None.

## Funding

This work was supported by The Medical and Health Appropriate Technology Development and Promotion Project of Guangxi Zhuang Autonomous Region (China) (No. S2018008); The Scientific Research & Technical Development Project of Wuming District, Nanning City (China) (No. 20200213 and 20220117); The Chinese Medical and Health Appropriate Technology Development and Promotion Project of Guangxi Zhuang Autonomous Region (China) (No. GZSY22-70).

## Appendix A Supplementary data

Multimedia component 1 **Figure S1** Extraction of cuproptosis-related lncRNAs features in breast cancer **(A)** A Sankey diagram visualizes the cuproptosis-related genes (CRGs) and co-expressed lncRNAs. **(B, C)** Lasso regression. **(D)** Forest plot of univariate Cox regression analysis of transcriptome differential expression between tumor and normal tissues. **(E)** Heat map of multivariate analysis of the correlation between lncRNAs and copper poisoning-related genes involved in model construction. Multimedia component 1

Multimedia component 2 **Figure S2** The risk scores and survival states of different risk group **(A)** Overall risk heat map and risk score. **(B)** Risk heat map and risk score of the training group. **(C)** Risk heat map and risk score of the test group. Multimedia component 2

Multimedia component 3 **Figure S3** Subgroup analysis of the prediction model. Multimedia component 3

Multimedia component 4 **Figure S4** Principal component analysis of **(A)** overall genes in the TCGA cohort, **(B)** the cuproptosis-related genes, **(C)** the cuproptosis-related lncRNAs, and **(D)** the lncRNAs included in the prognostic model. **(E)** Results of Gene Ontology analyses in the TCGA cohort. **(F)** Results of Kyoto Encyclopedia of Genes and Genomes analyses in the TCGA cohort. Multimedia component 4

Multimedia component 5 **Figure S5** Immune microenvironment features of breast cancer indicated by a cuproptosis-associated long non-coding RNA signature **(A)** The hierarchical clustering heat diagram of ssGSEA enrichment score. **(B)** Survival analysis of tumor mutational burden (TMB). **(C)** Violin diagram of the variation analysis of TMB. **(D)** Heatmap of immune function. Multimedia component 5

Multimedia component 6 **Figure S6** Boxplot of the drugs with the statistical difference in drug sensitivity between the high- and low-risk groups. Multimedia component 6

Multimedia component 7 **Figure S7** The expression levels of AL137847.1, LRRC8C−DT, and NIFK−AS1. Multimedia component 7

Multimedia component 8 **Table S1** Materials and methods. Multimedia component 8

Multimedia component 9 **Table S2** Forward and reverse sequences of primers. Multimedia component 9

Multimedia component 10 **Table S3** The baseline clinical characteristics of included patients. Multimedia component 10
