# Supplementary figures and images for: Prognosis prediction and immune microenvironment features of breast cancer indicated by a cuproptosis-associated long non-coding RNA signature
Source: Genes Dis. 2023 Sep 22;11(5):101110. doi: 10.1016/j.gendis.2023.101110 (PMC11177056; doi:10.1016/j.gendis.2023.101110)

A

Cuproptosis

lncRNA

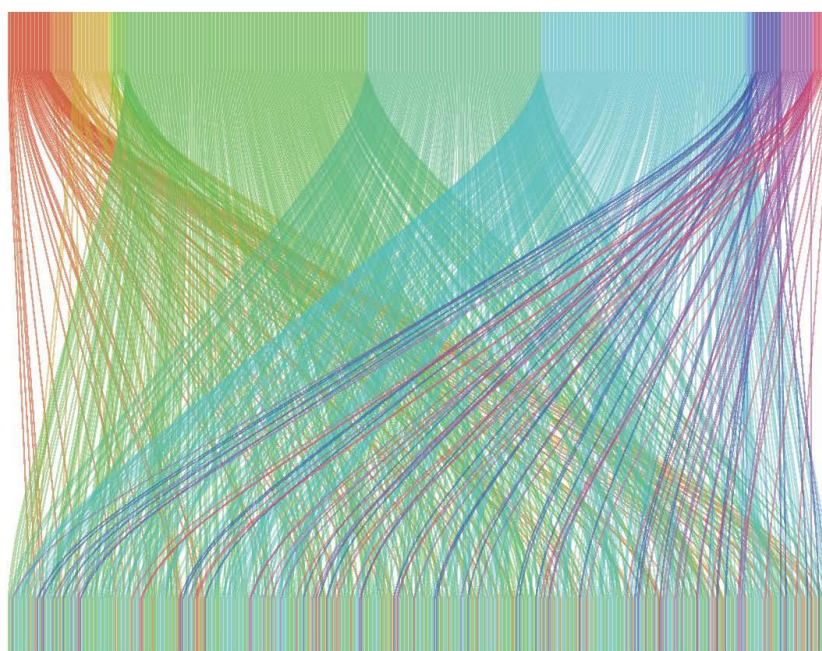

Cuproptosis

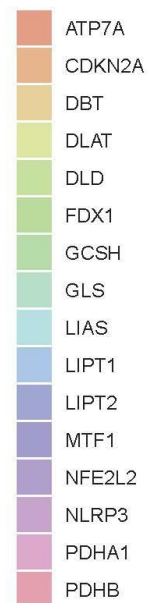

B

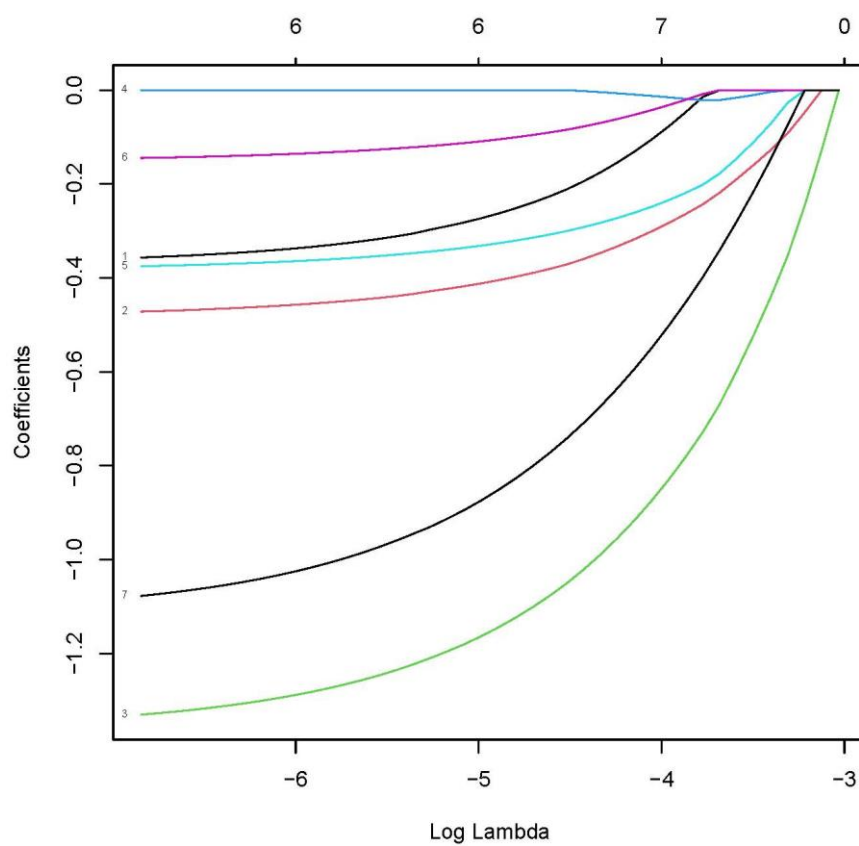

C

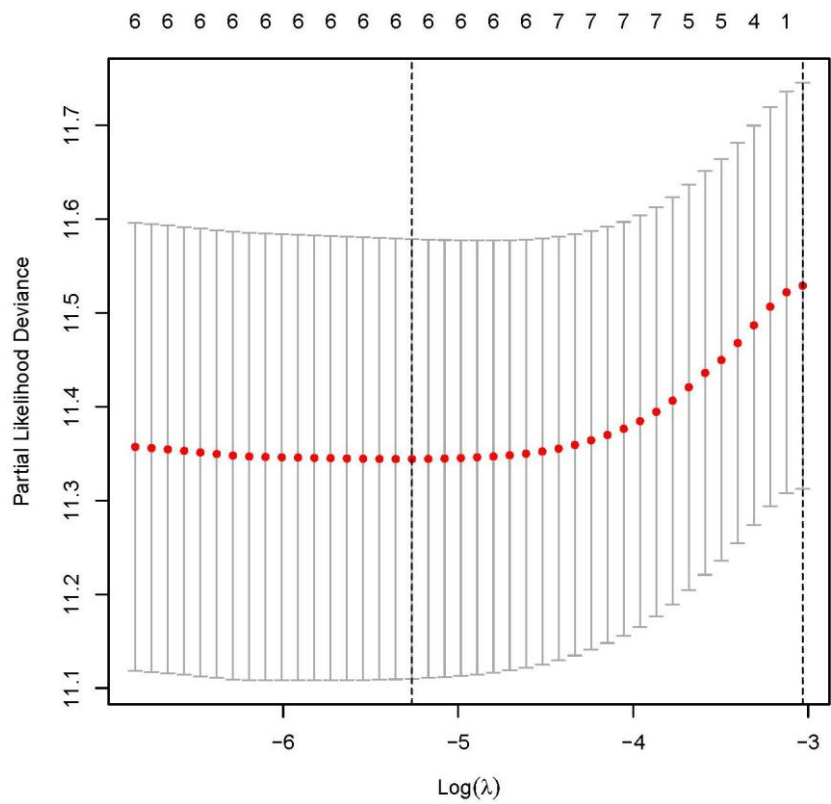

D

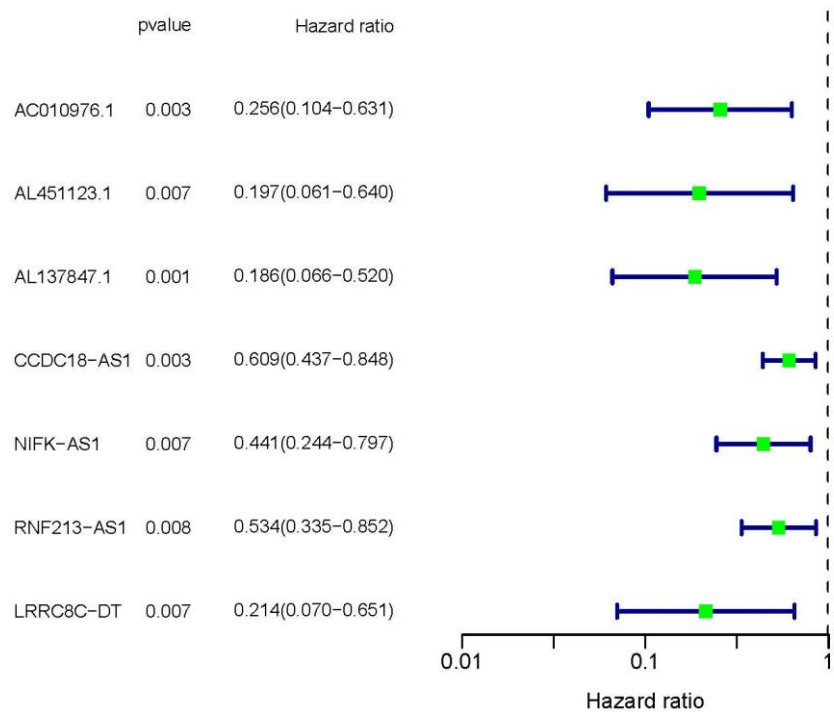

E

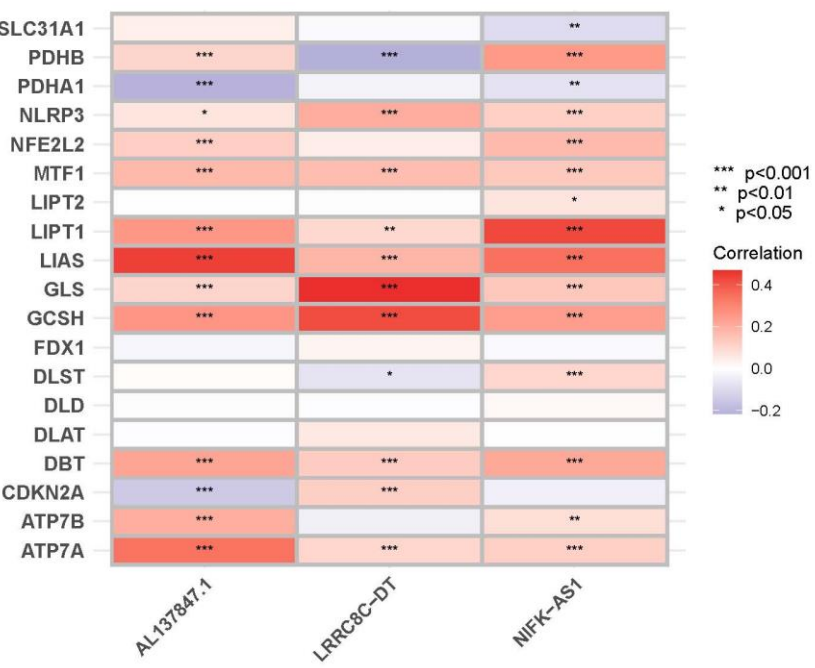

Supplement: Multimedia component 1 — Figure S1 Extraction of cuproptosis-related lncRNAs features in breast cancer (A) A Sankey diagram visualizes the cuproptosis-related genes (CRGs) and co-expressed lncRNAs. (B, C) Lasso regression. (D) Forest plot of univariate Cox regression analysis of transcriptome differential expression between tumor and normal tissues. (E) Heat map of multivariate analysis of the correlation between lncRNAs and copper poisoning-related genes involved in model construction. [file mmc1.pdf]

A

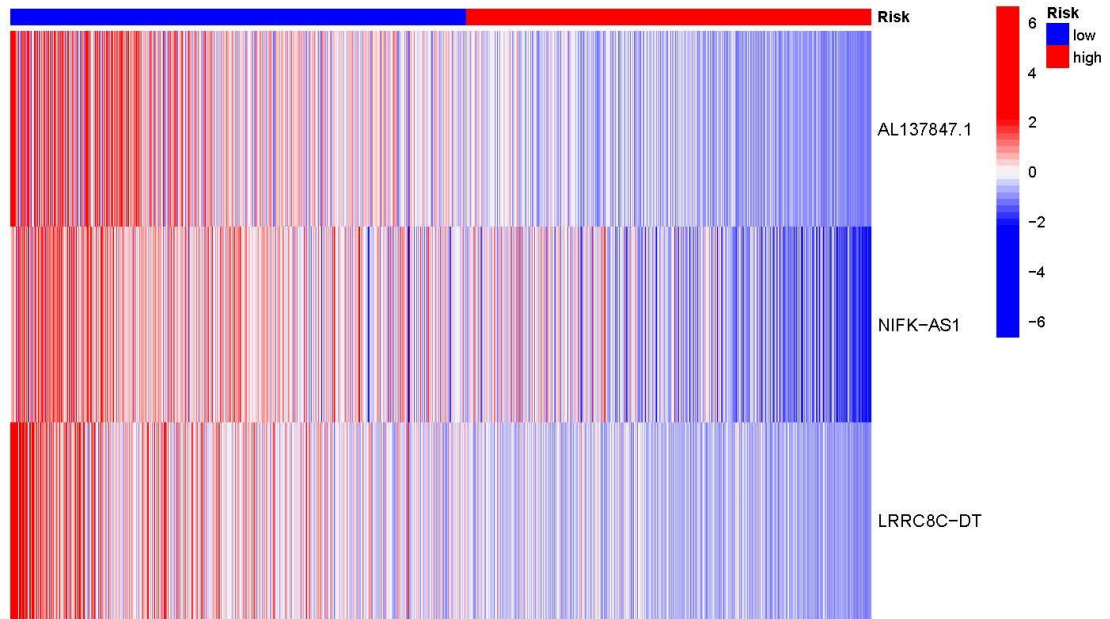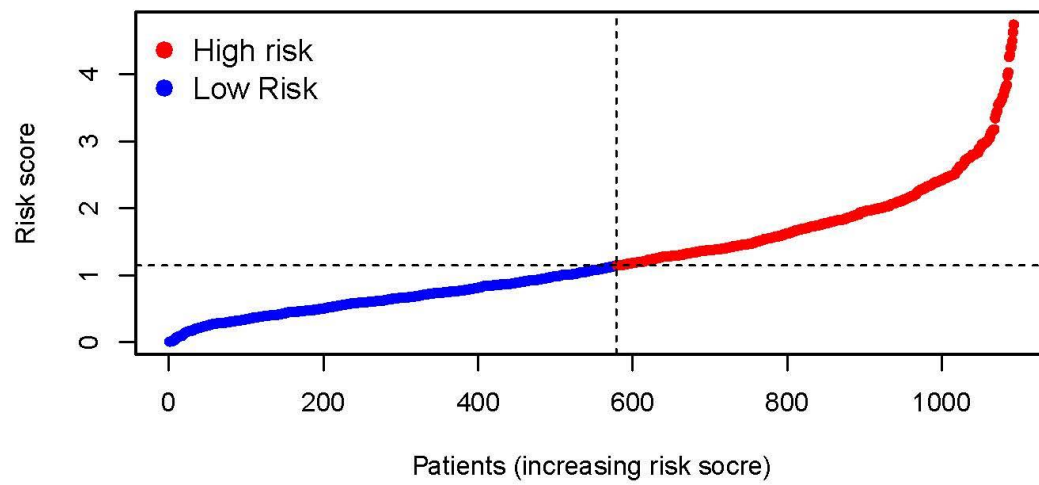

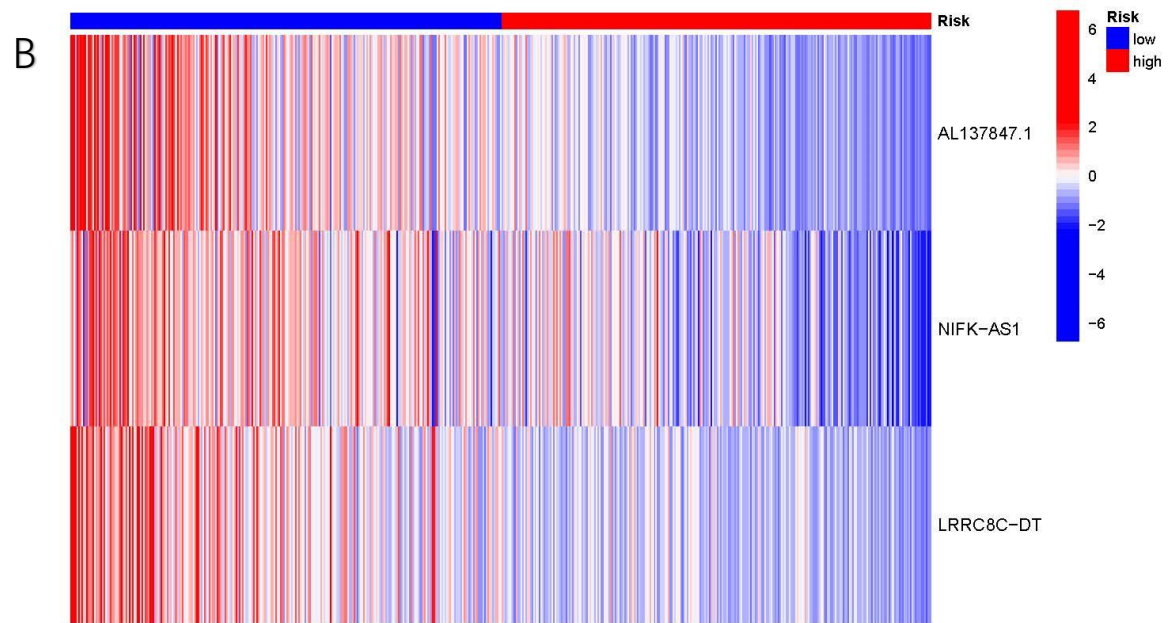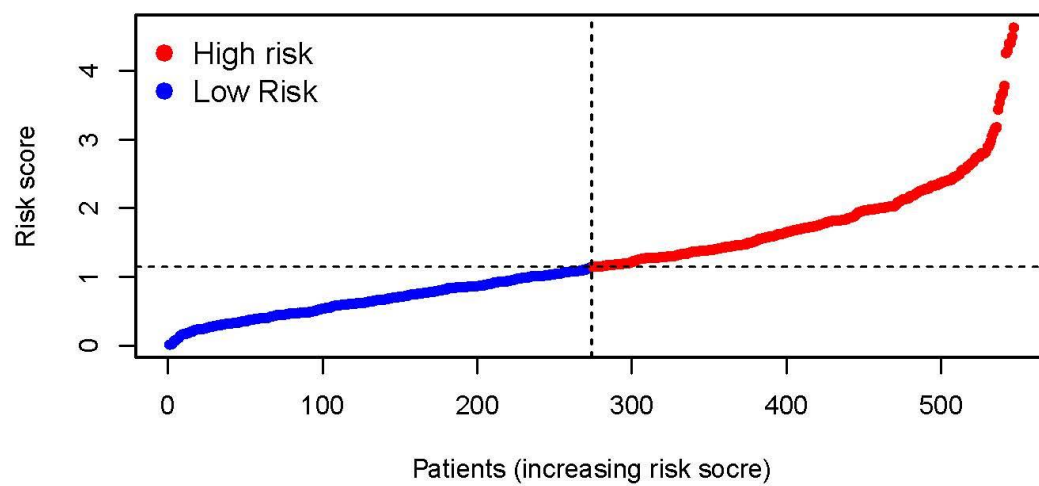

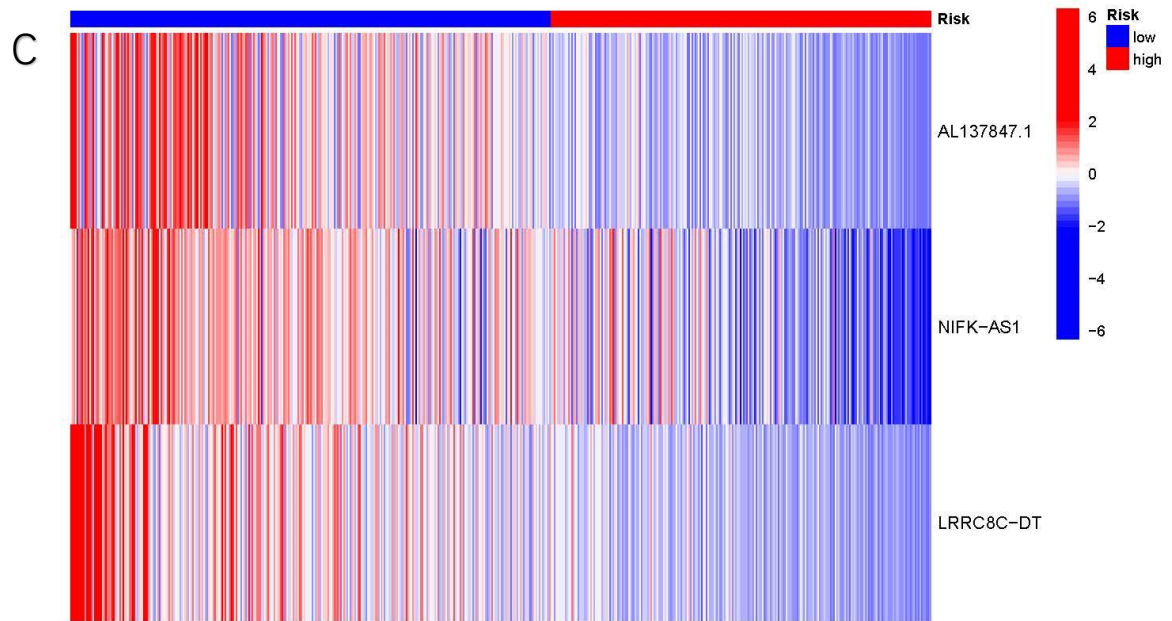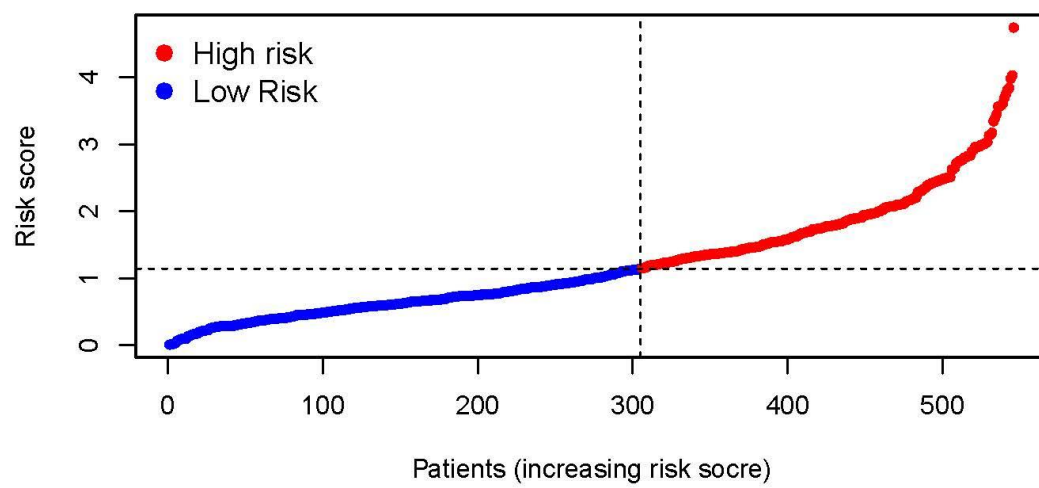

Supplement: Multimedia component 2 — Figure S2 The risk scores and survival states of different risk group (A) Overall risk heat map and risk score. (B) Risk heat map and risk score of the training group. (C) Risk heat map and risk score of the test group. [file mmc2.pdf]

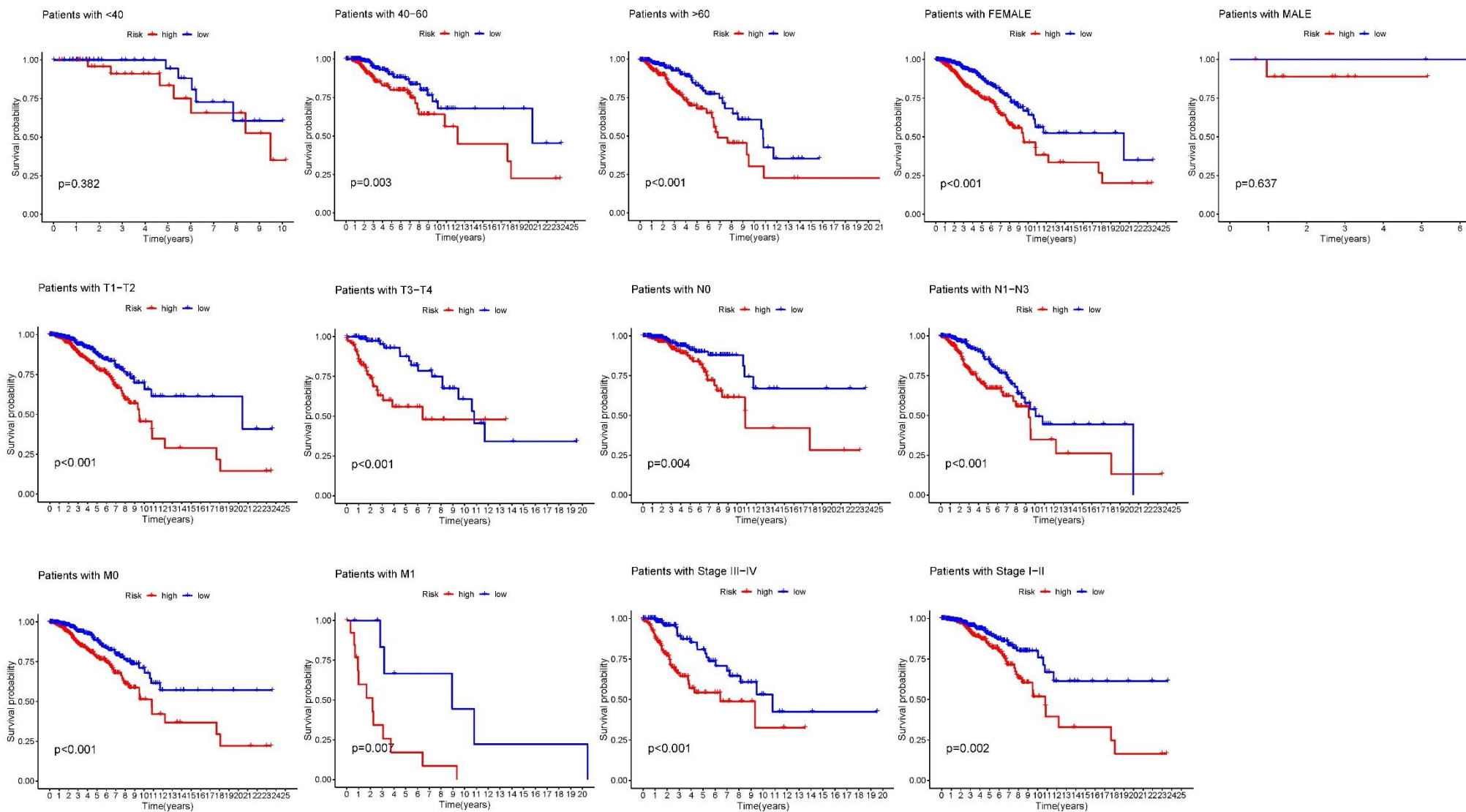

Supplement: Multimedia component 3 — Figure S3 Subgroup analysis of the prediction model. [file mmc3.pdf]

A

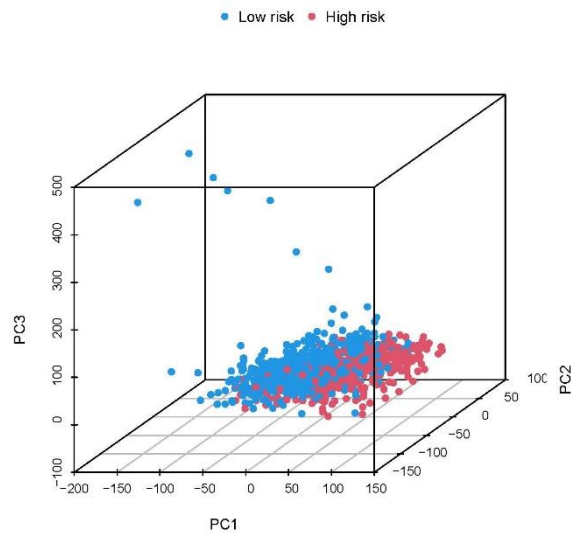

B

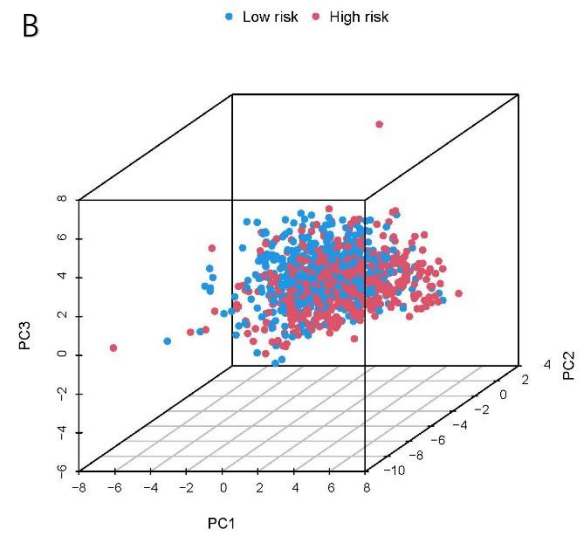

C

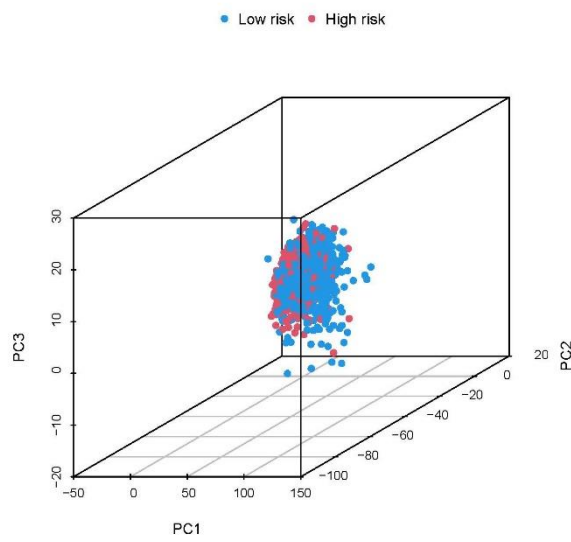

D

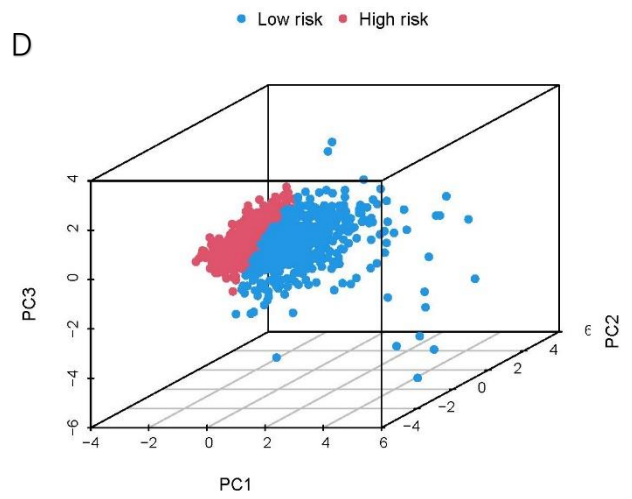

E

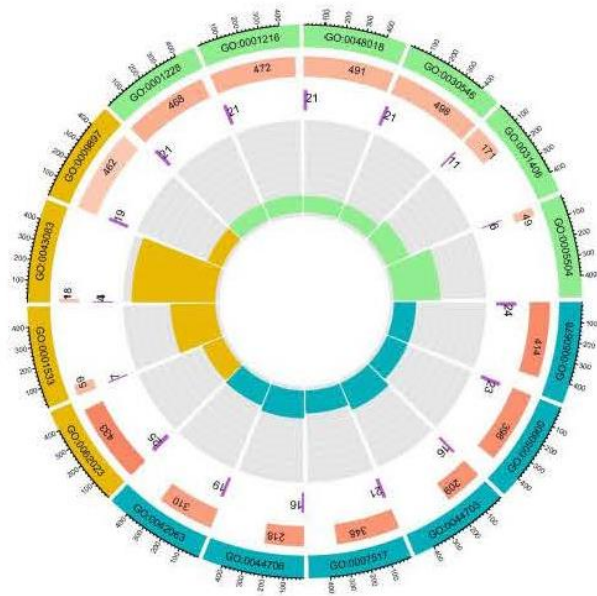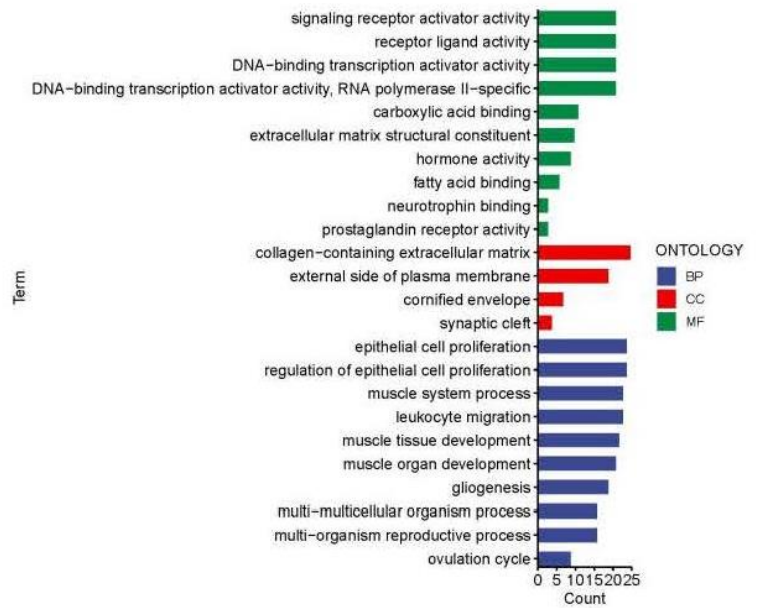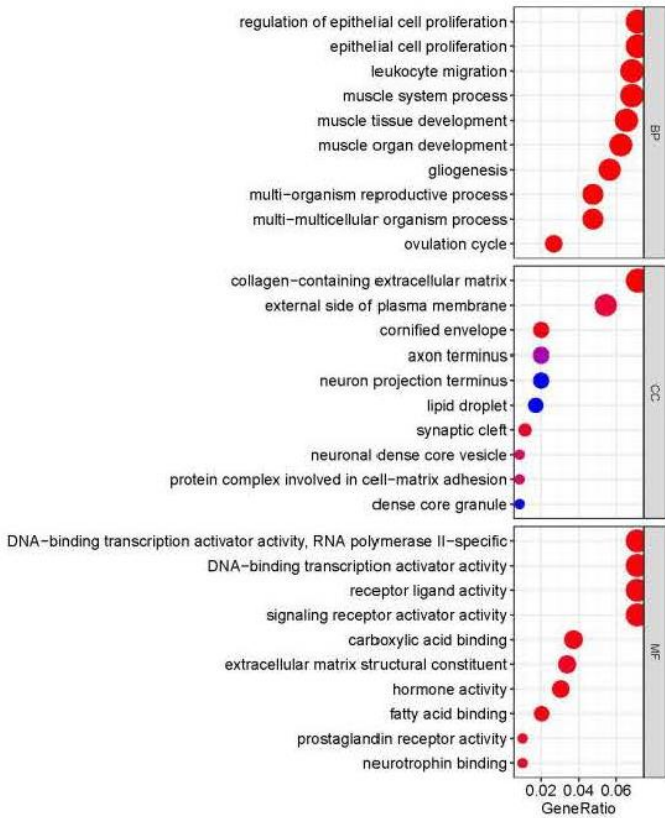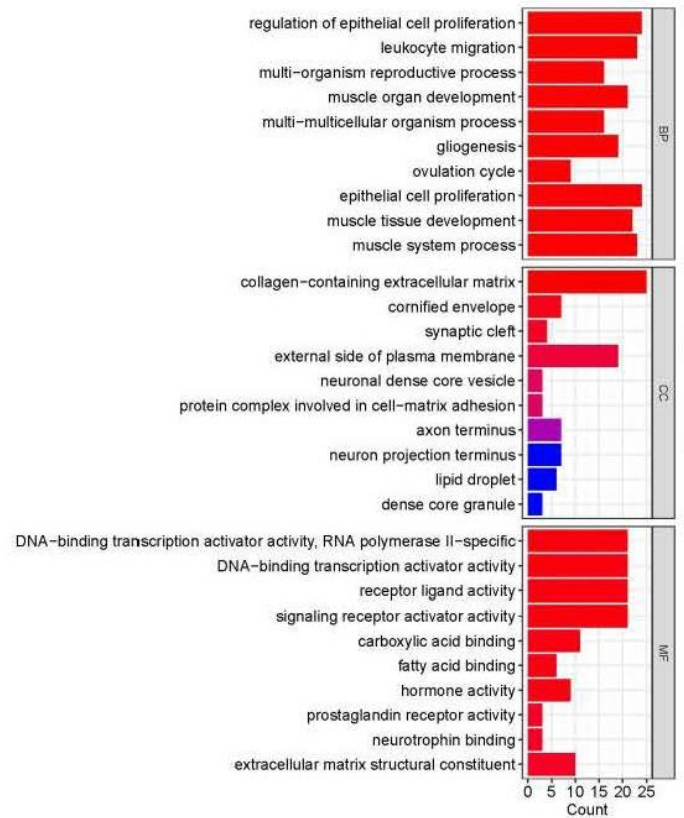

F

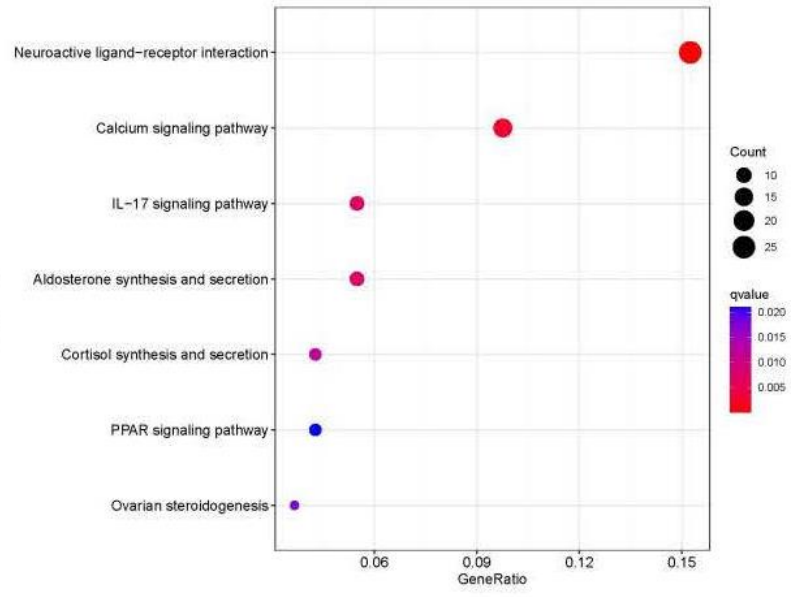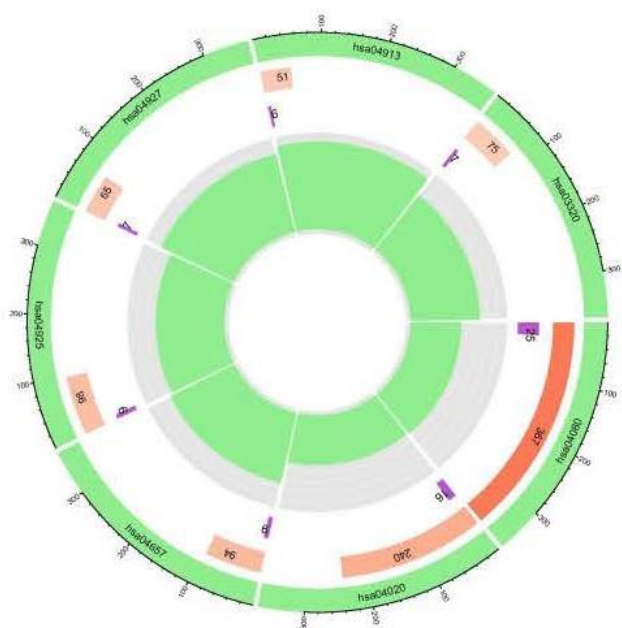

Supplement: Multimedia component 4 — Figure S4 Principal component analysis of (A) overall genes in the TCGA cohort, (B) the cuproptosis-related genes, (C) the cuproptosis-related lncRNAs, and (D) the lncRNAs included in the prognostic model. (E) Results of Gene Ontology analyses in the TCGA cohort. (F) Results of Kyoto Encyclopedia of Genes and Genomes analyses in the TCGA cohort. [file mmc4.pdf]

A

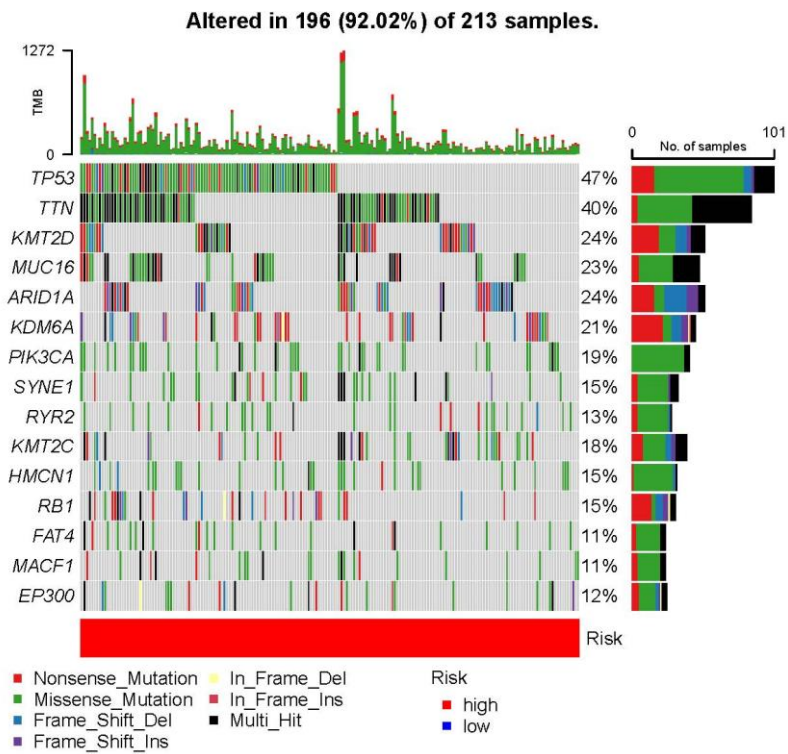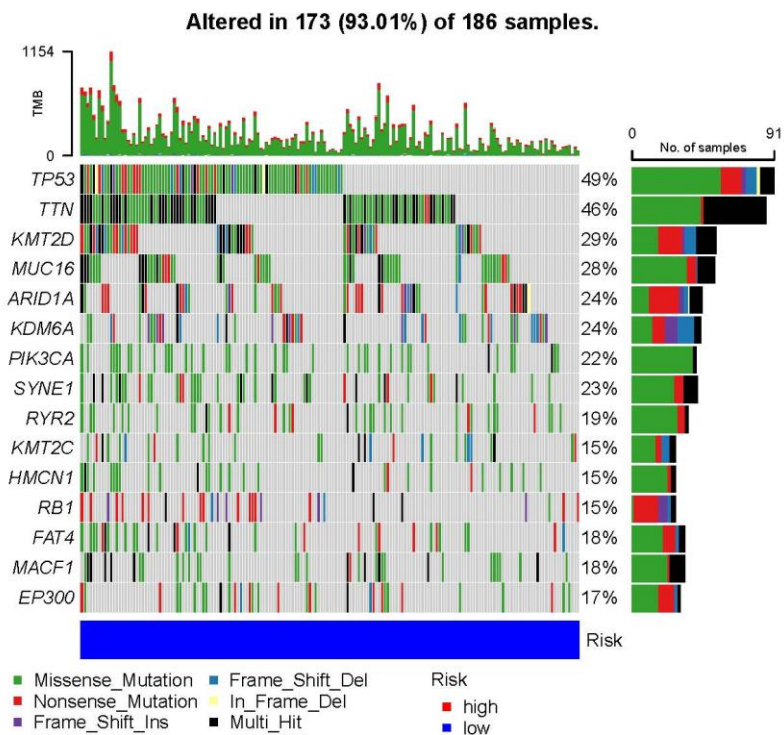

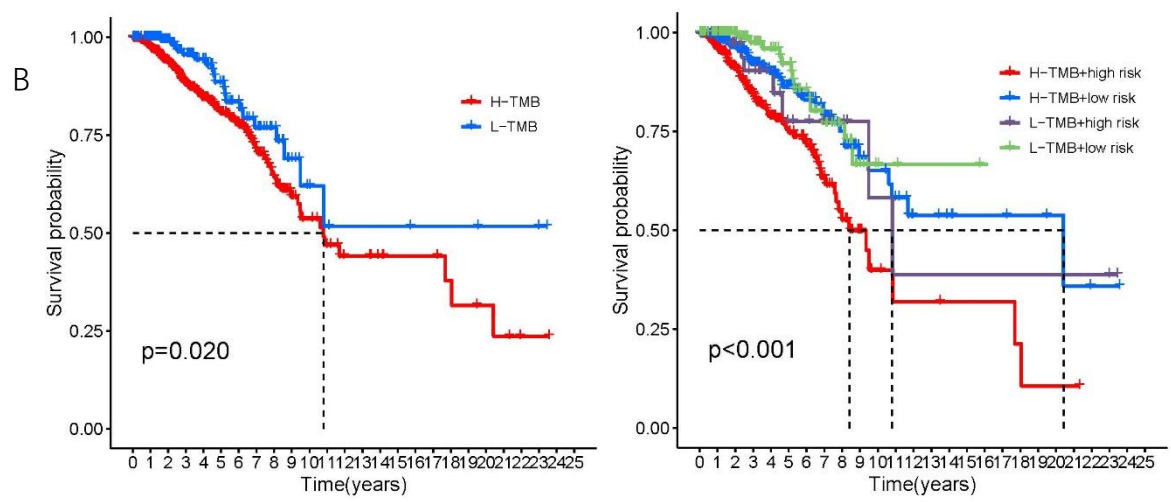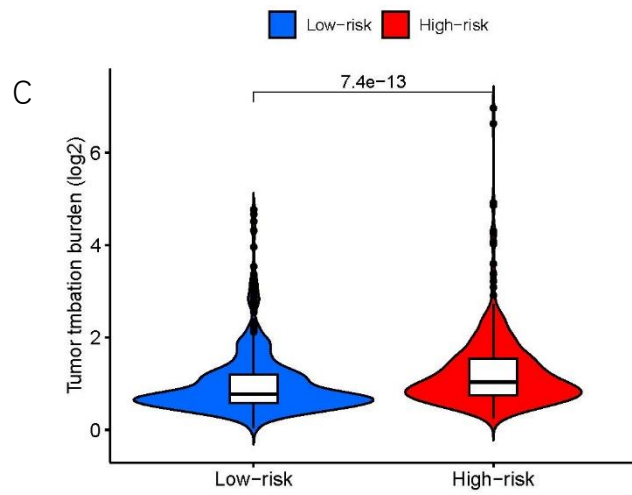

D

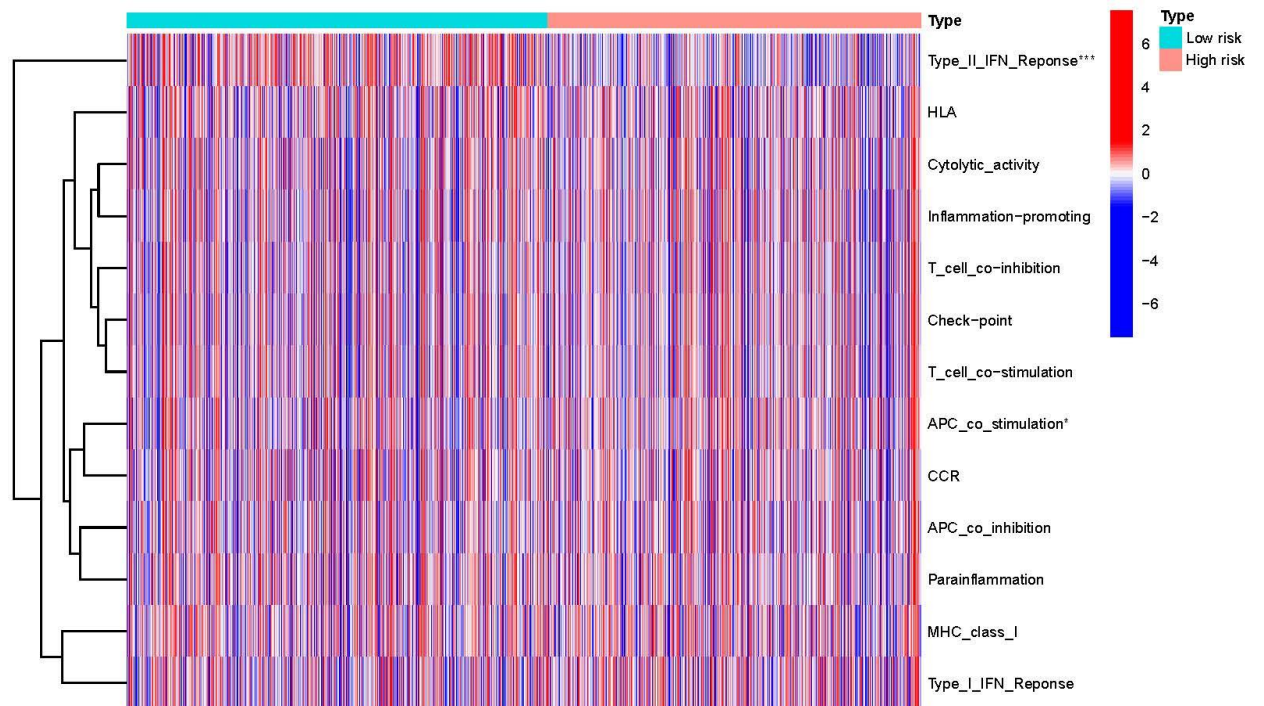

Supplement: Multimedia component 5 — Figure S5 Immune microenvironment features of breast cancer indicated by a cuproptosis-associated long non-coding RNA signature (A) The hierarchical clustering heat diagram of ssGSEA enrichment score. (B) Survival analysis of tumor mutational burden (TMB). (C) Violin diagram of the variation analysis of TMB. (D) Heatmap of immune function. [file mmc5.pdf]

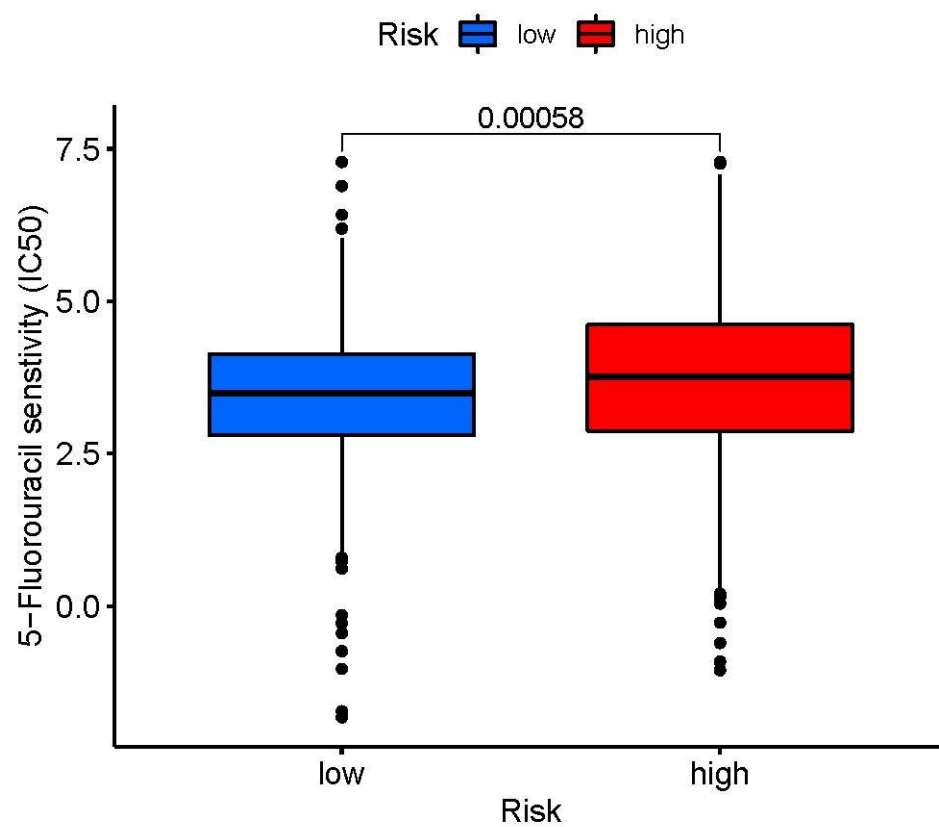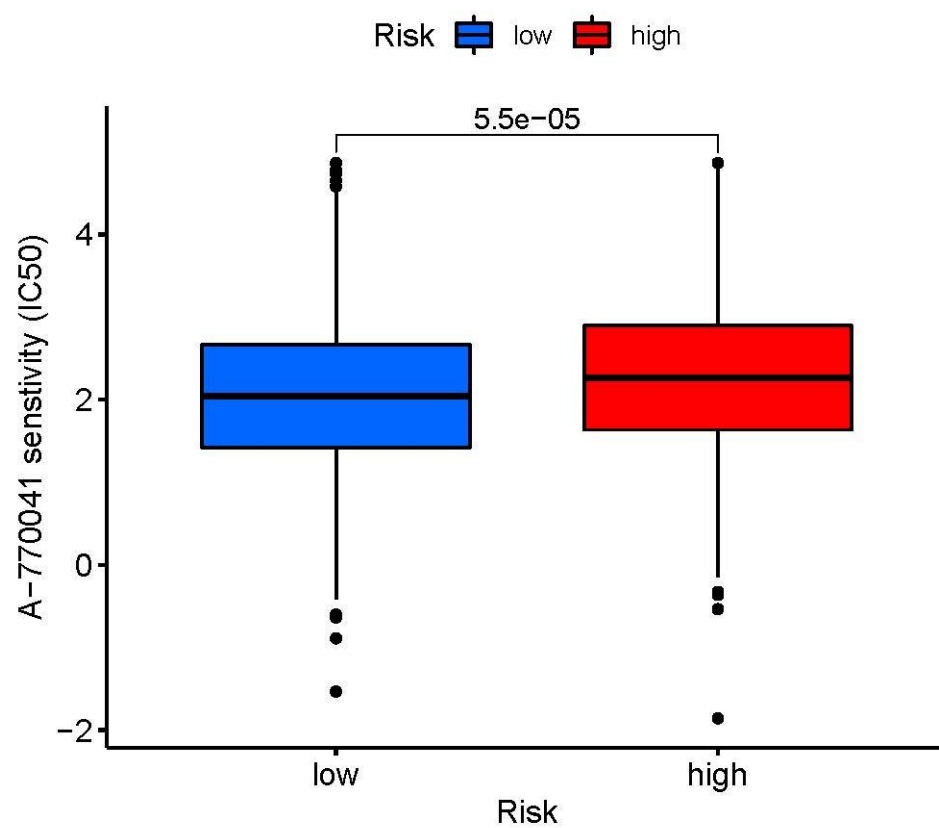

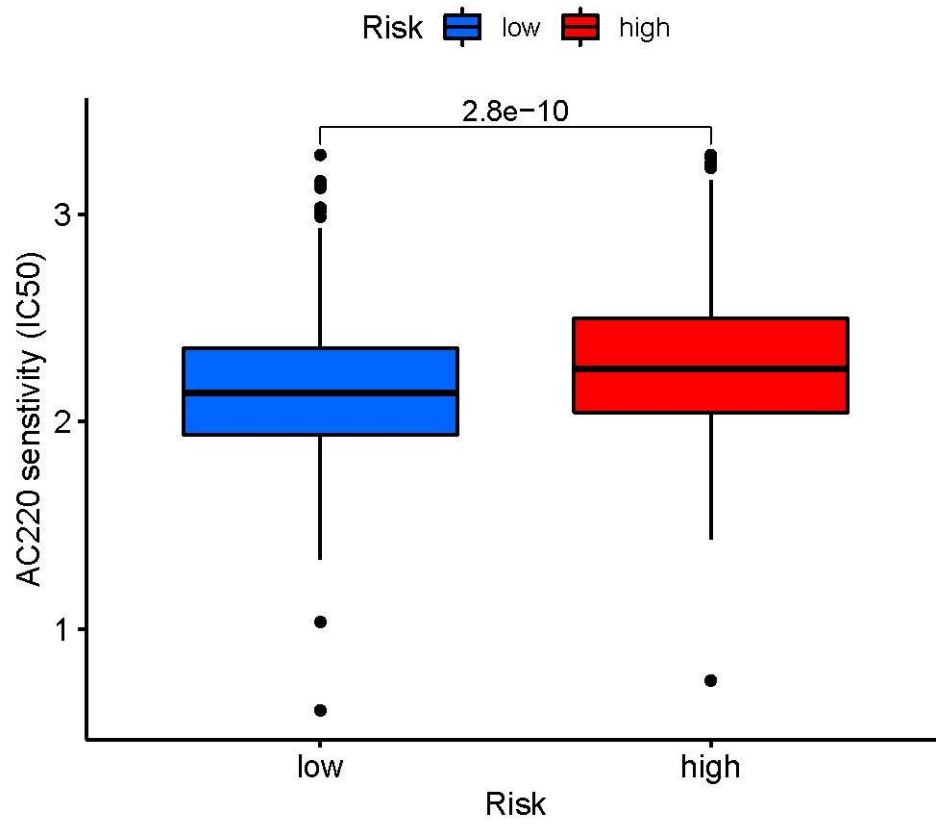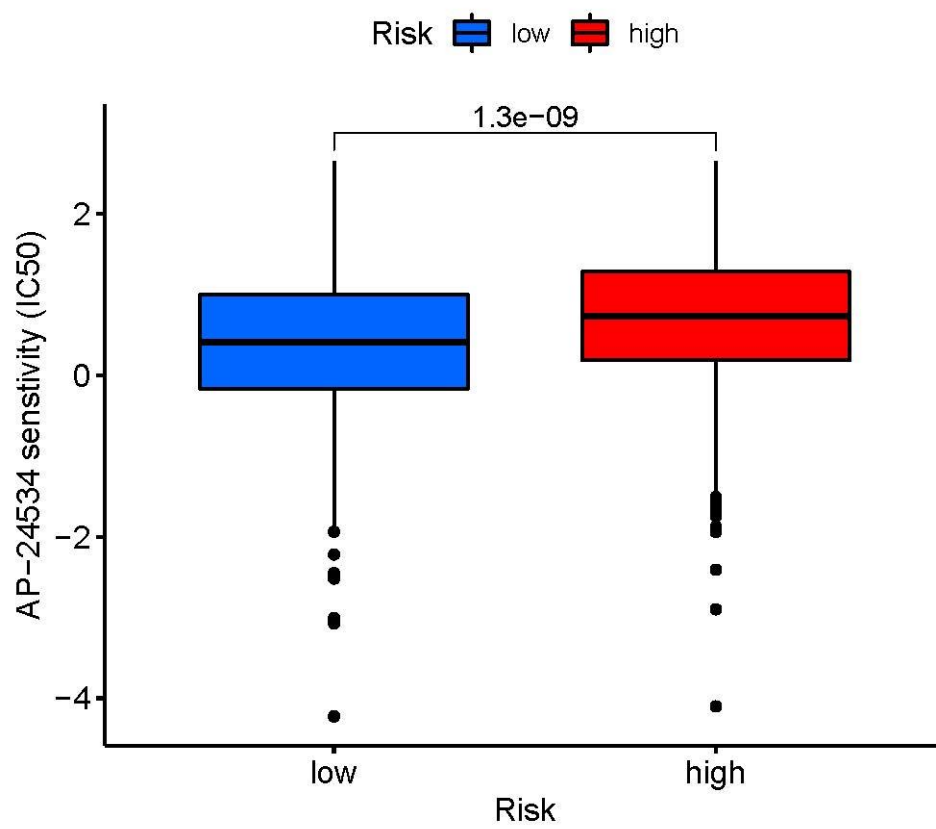

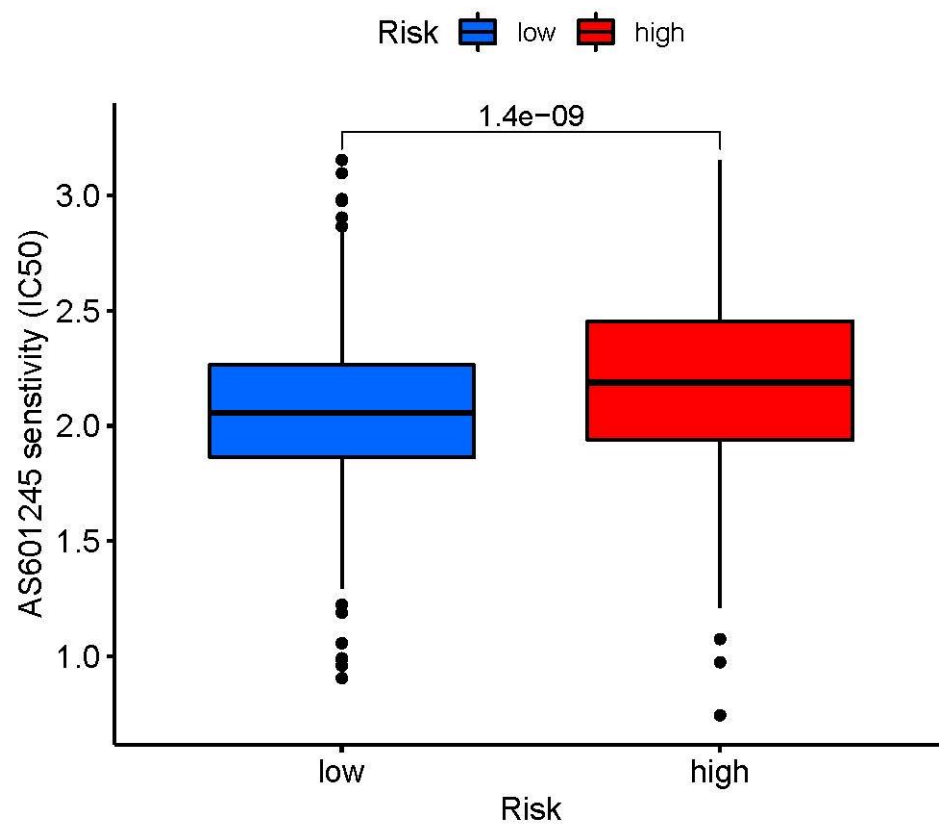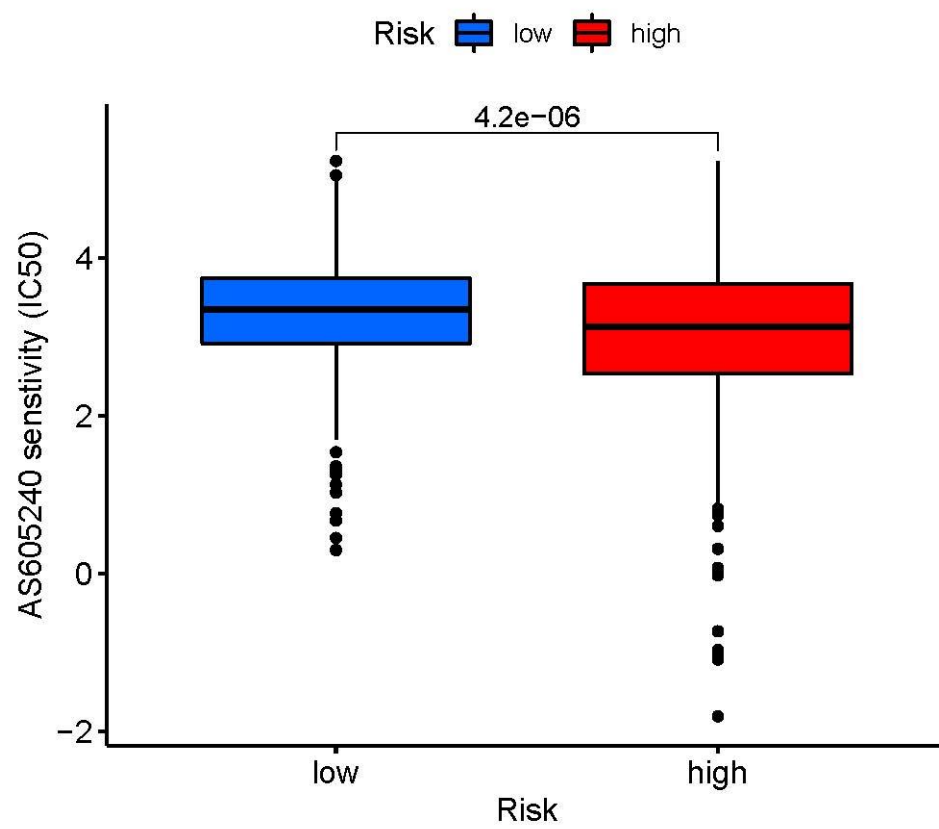

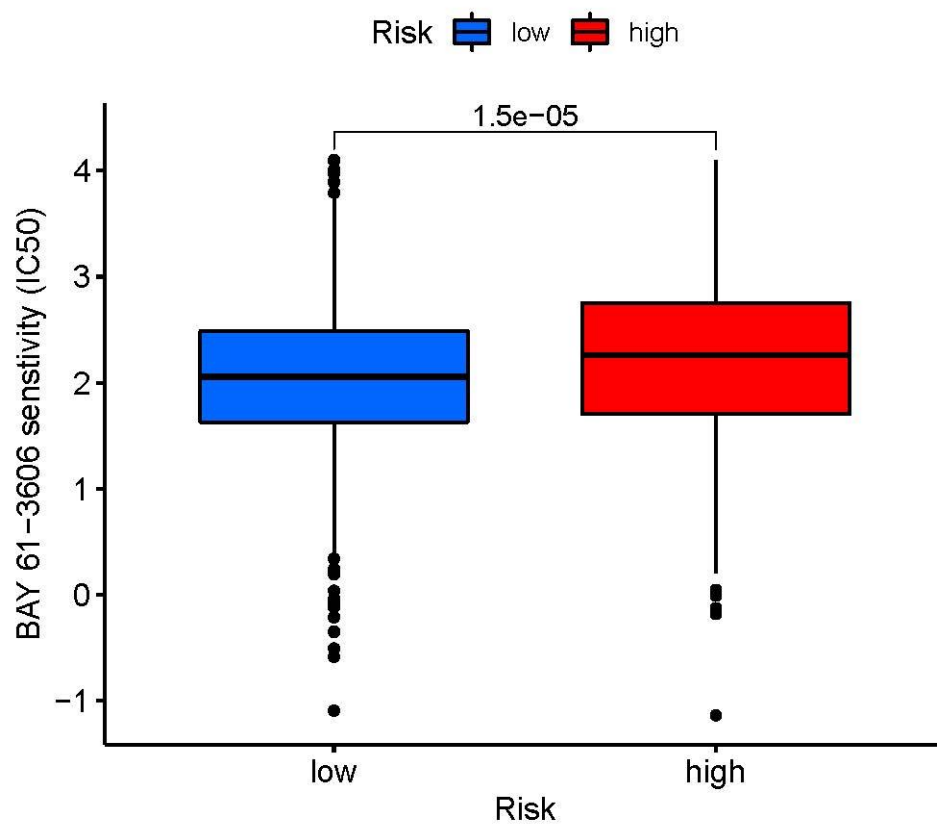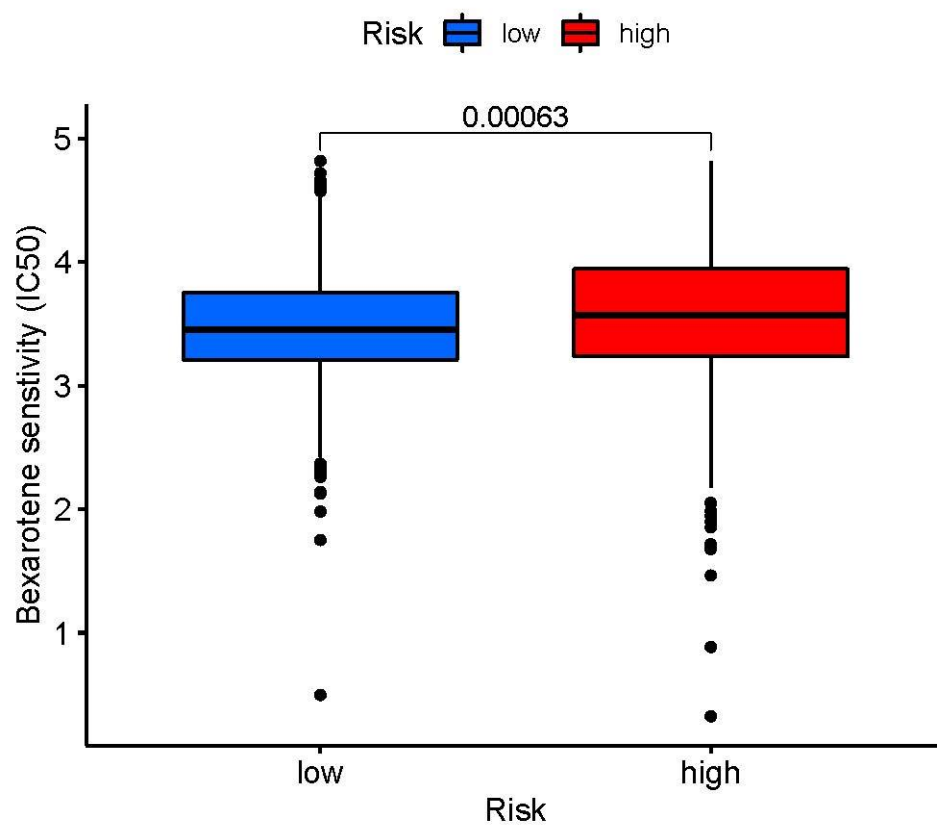

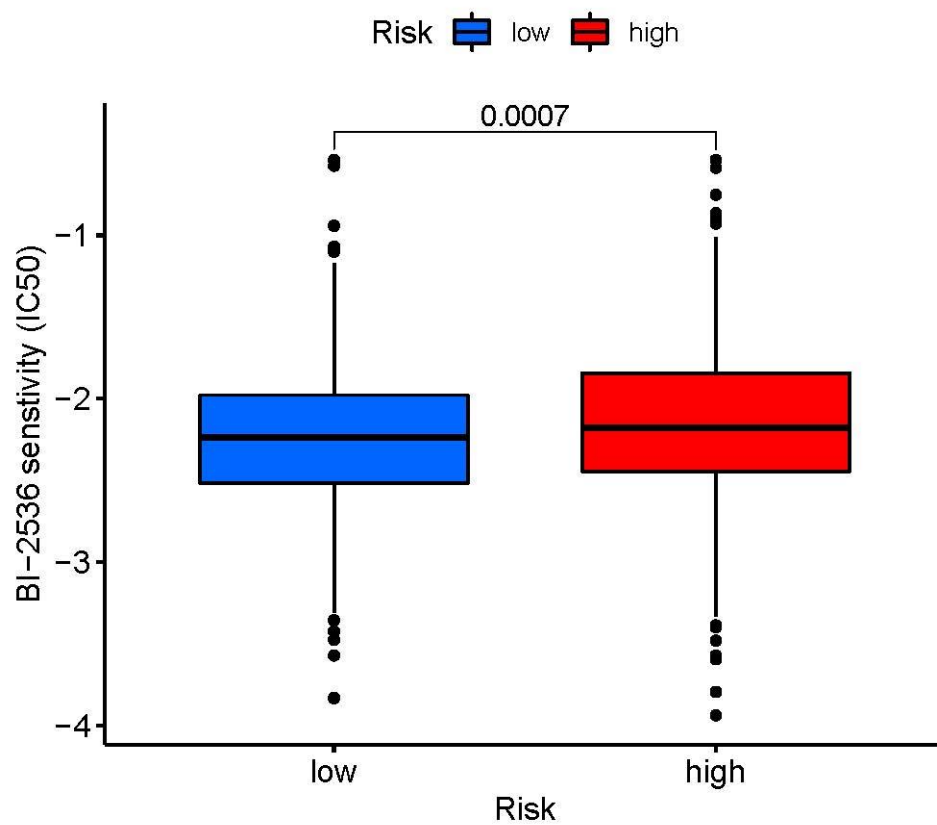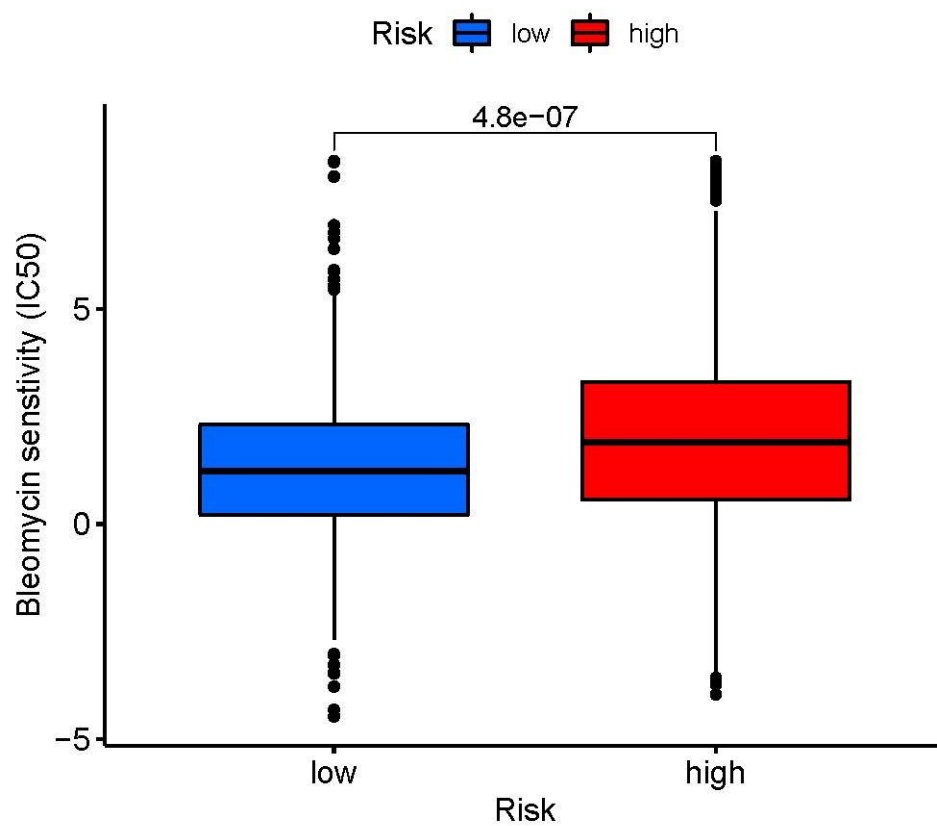

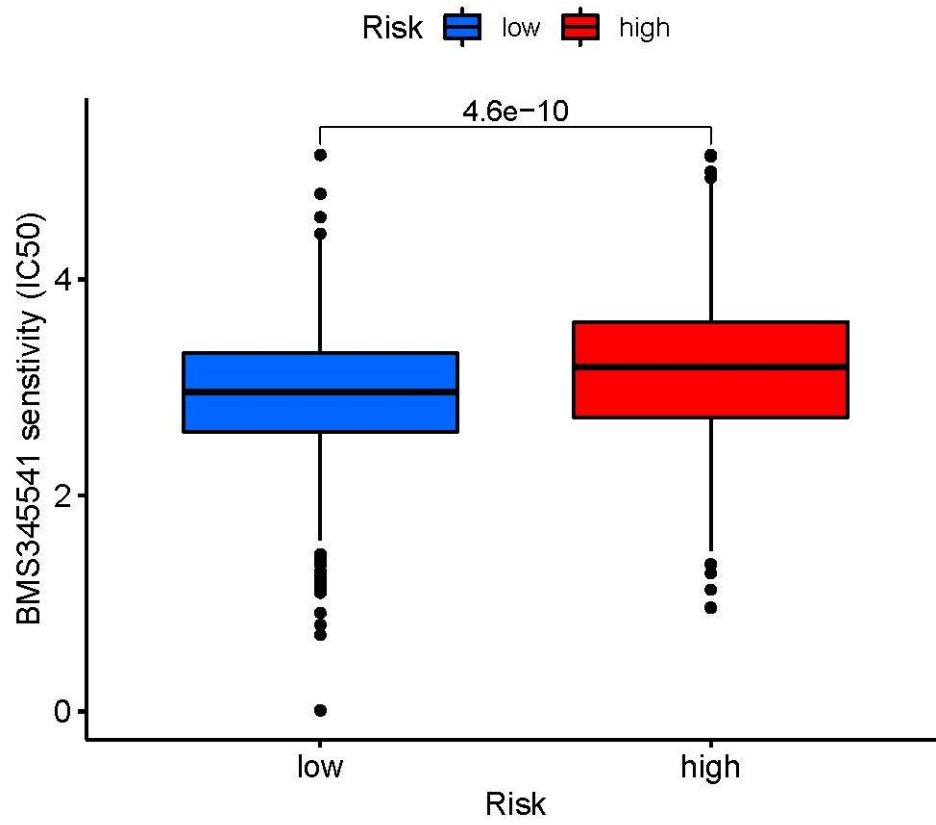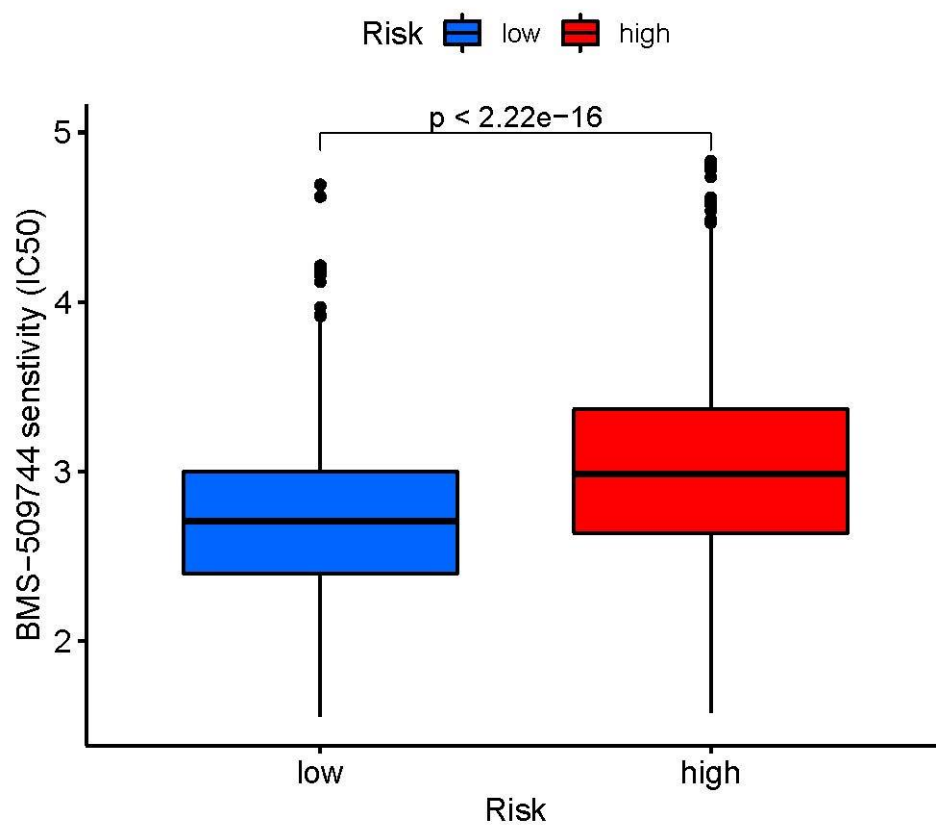

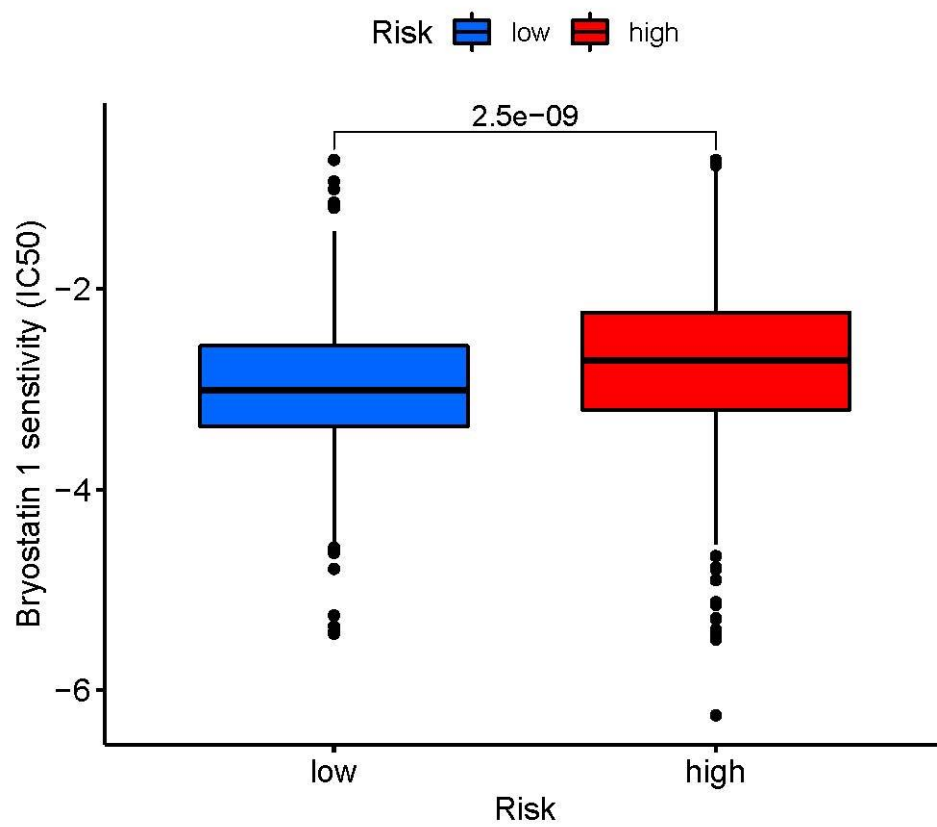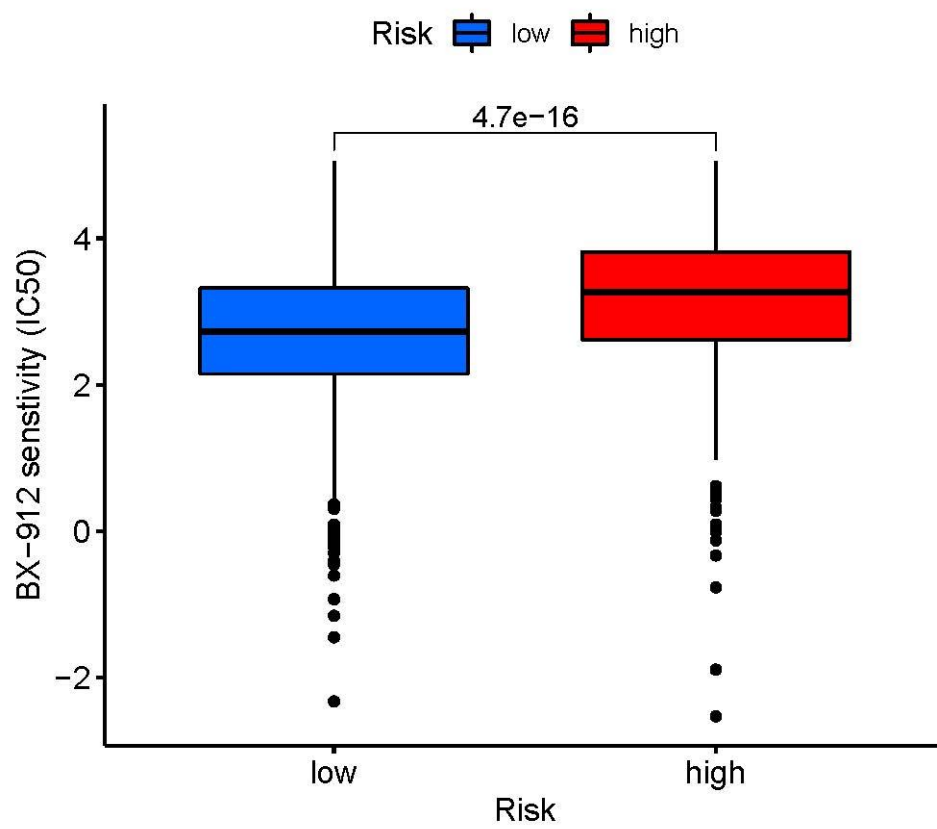

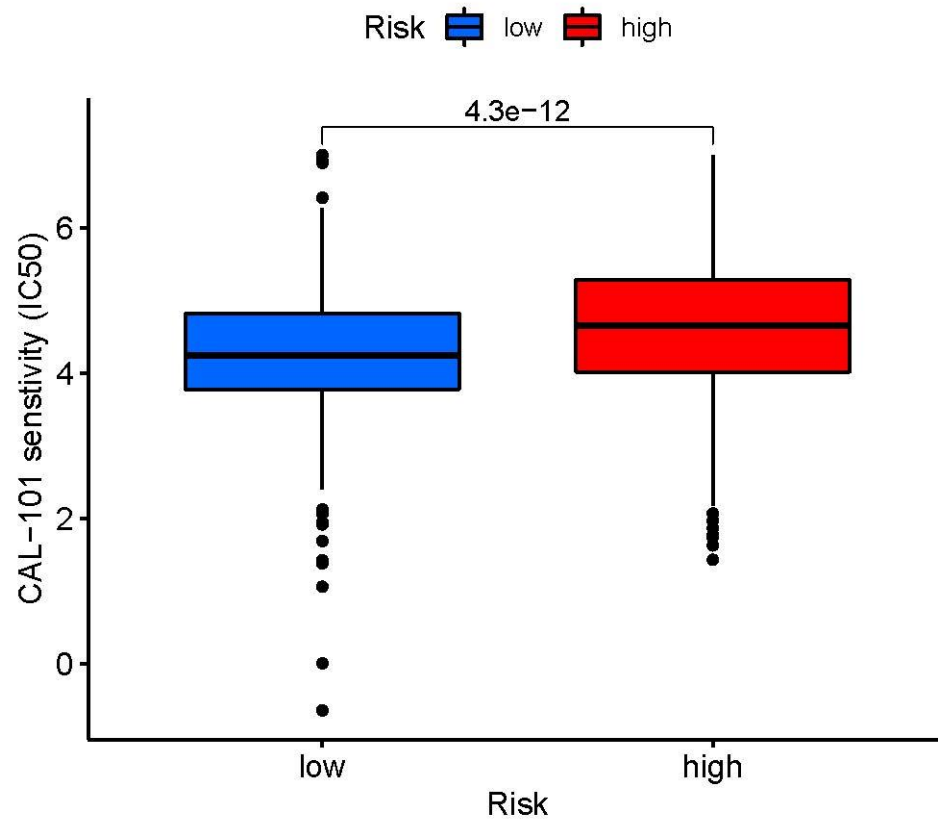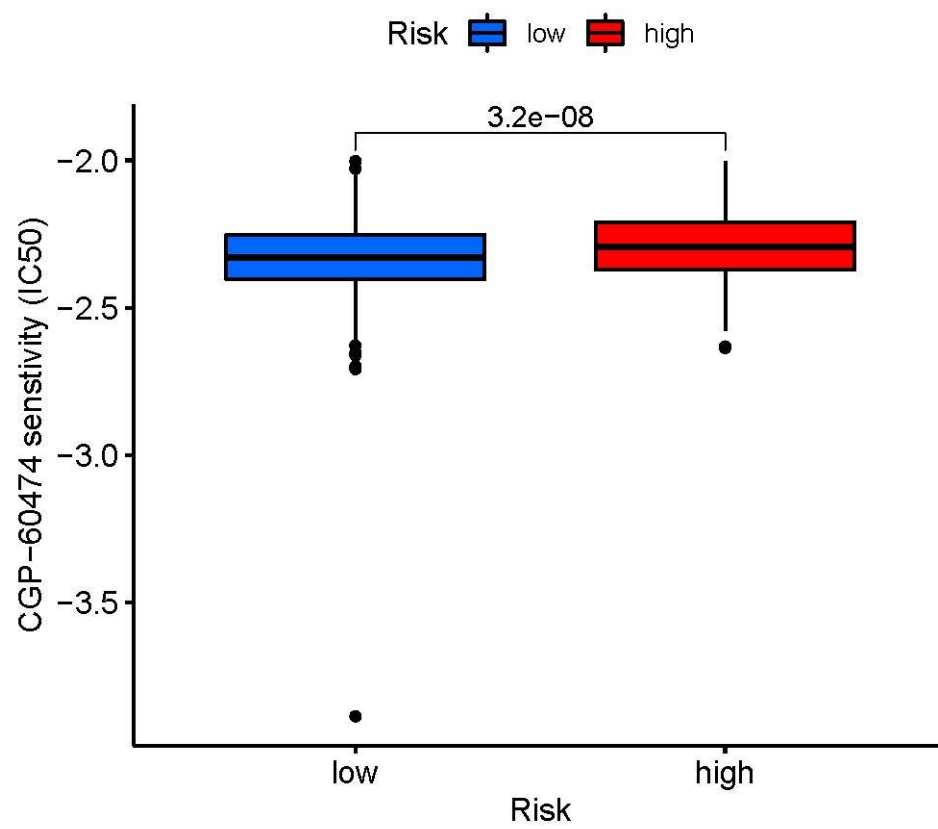

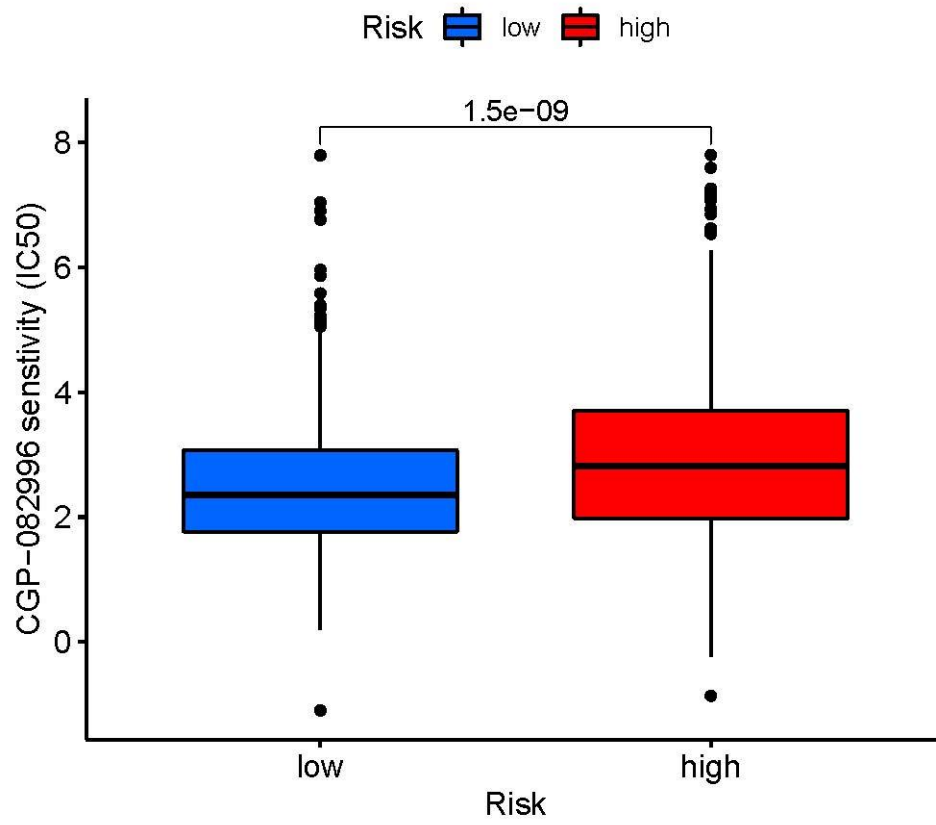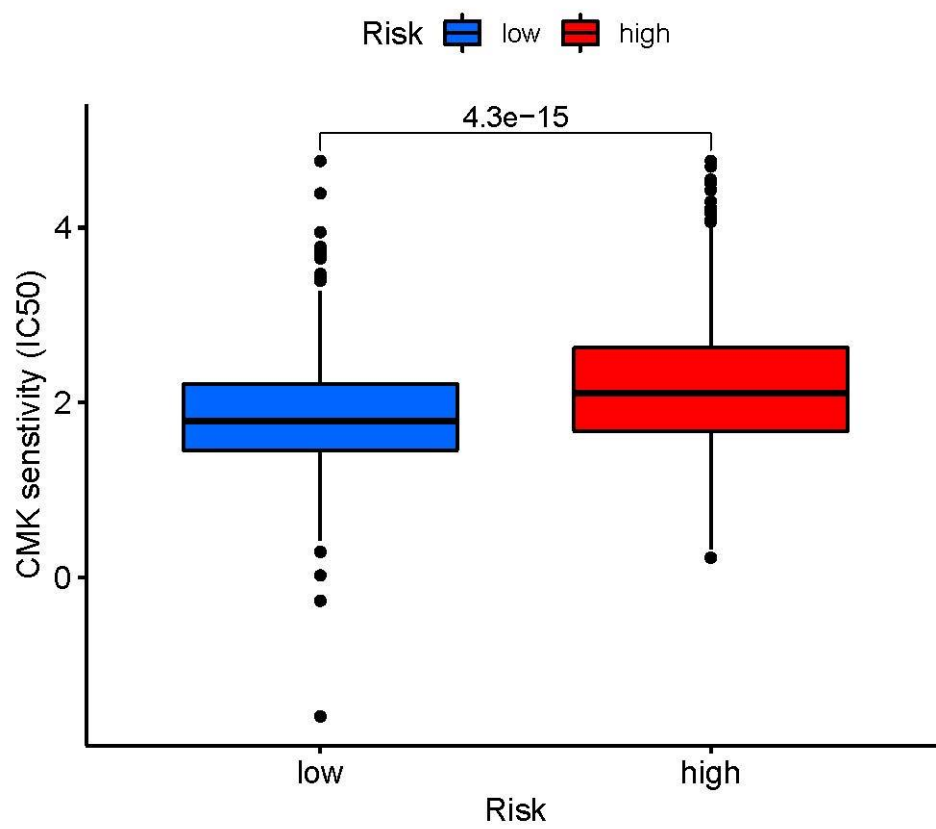

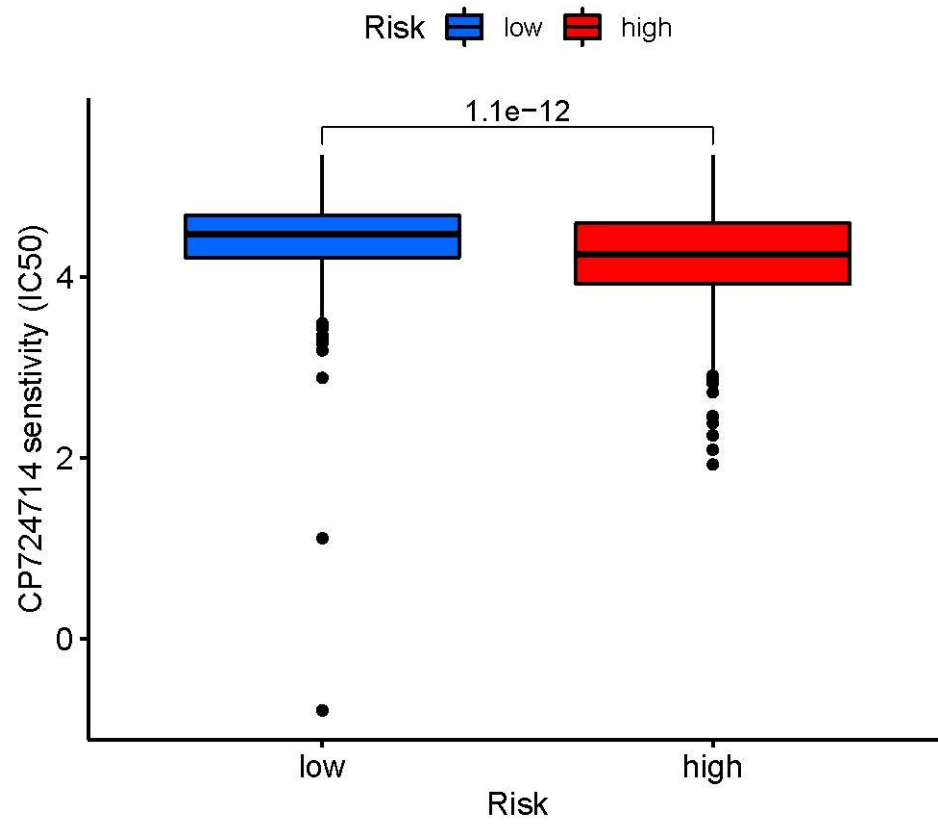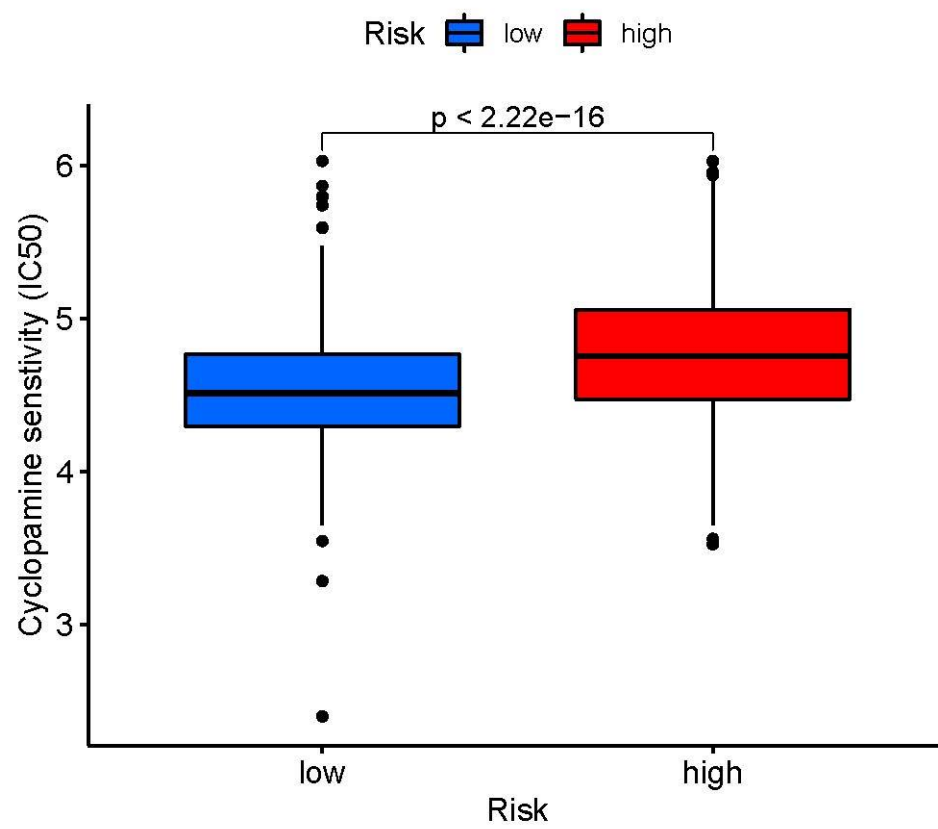

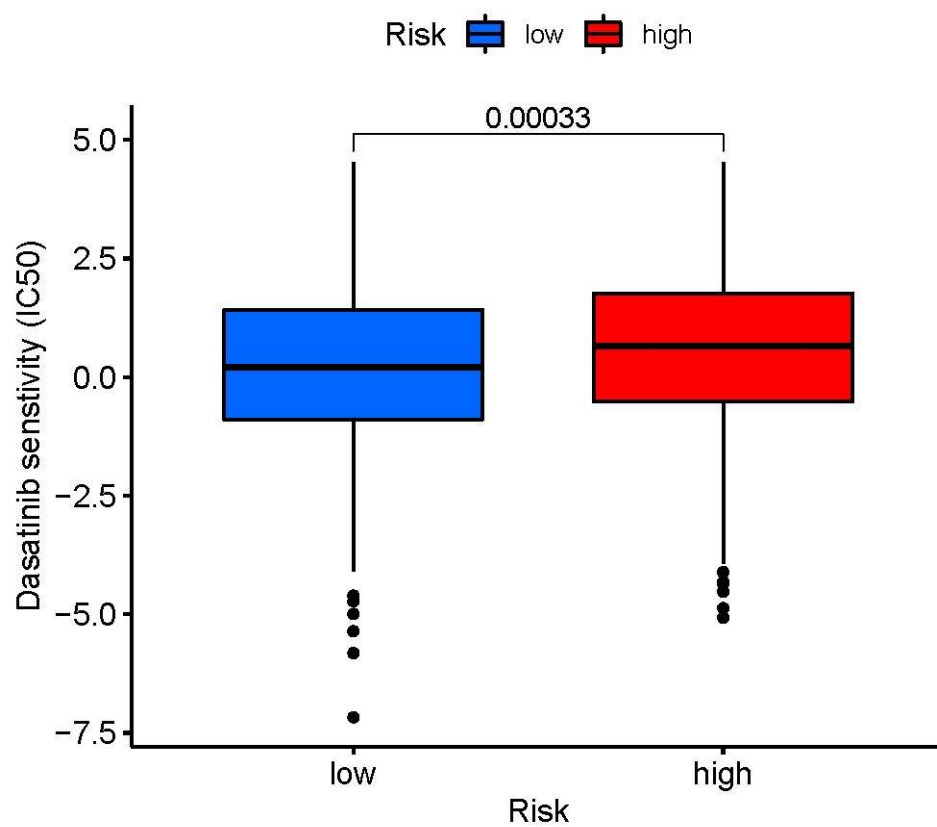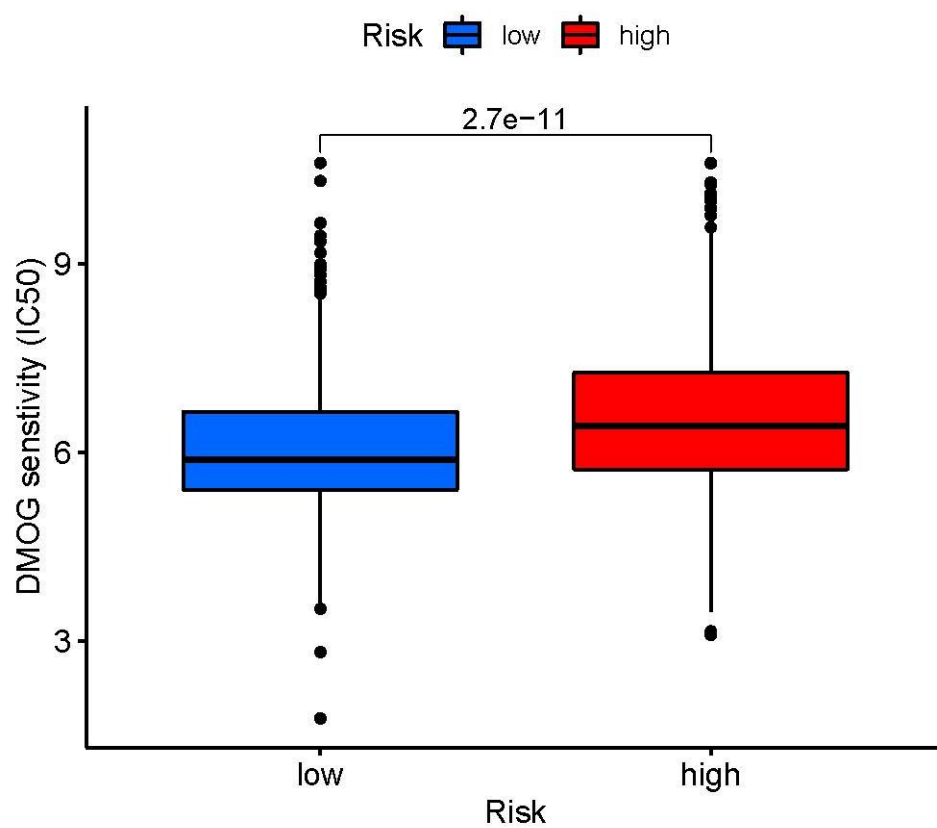

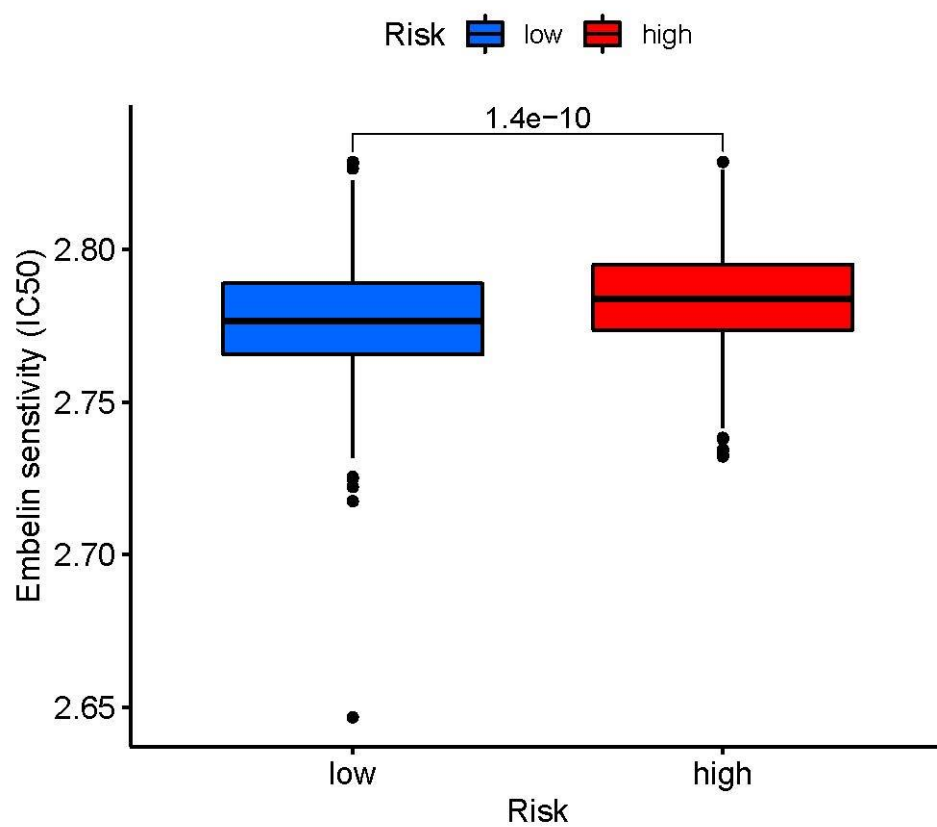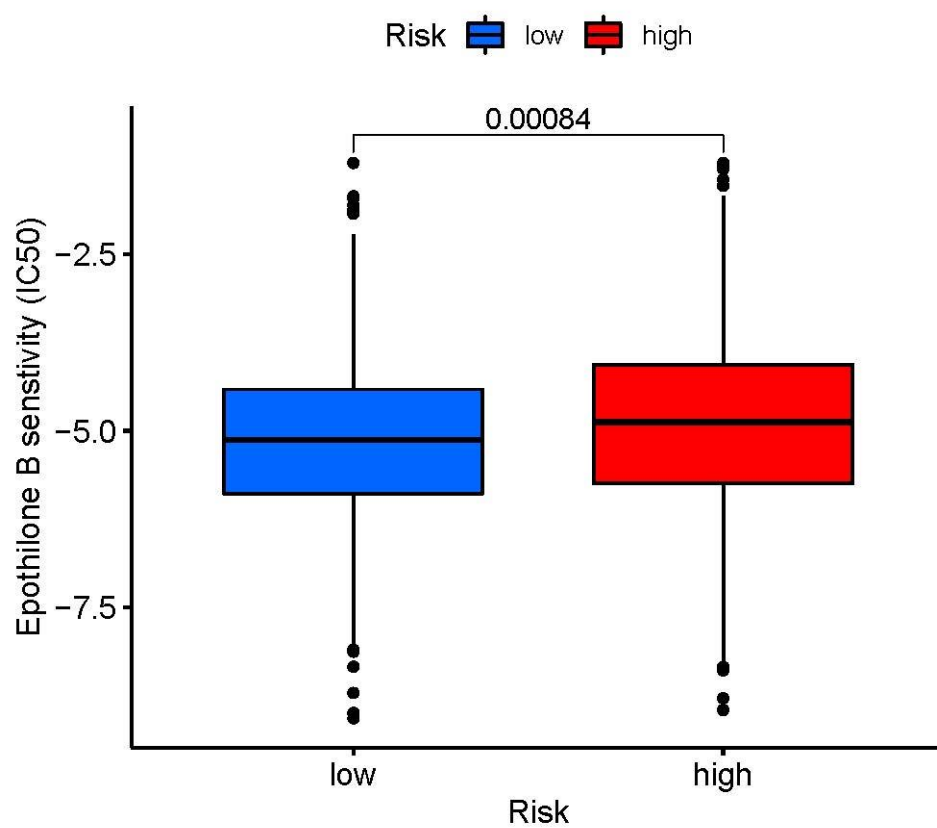

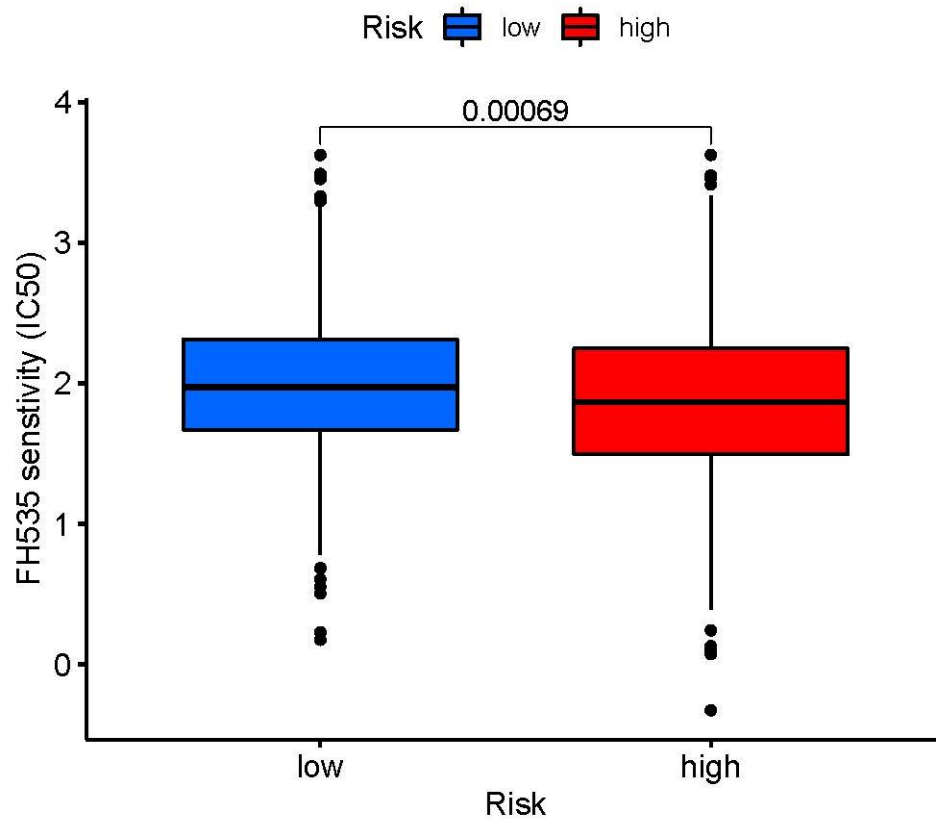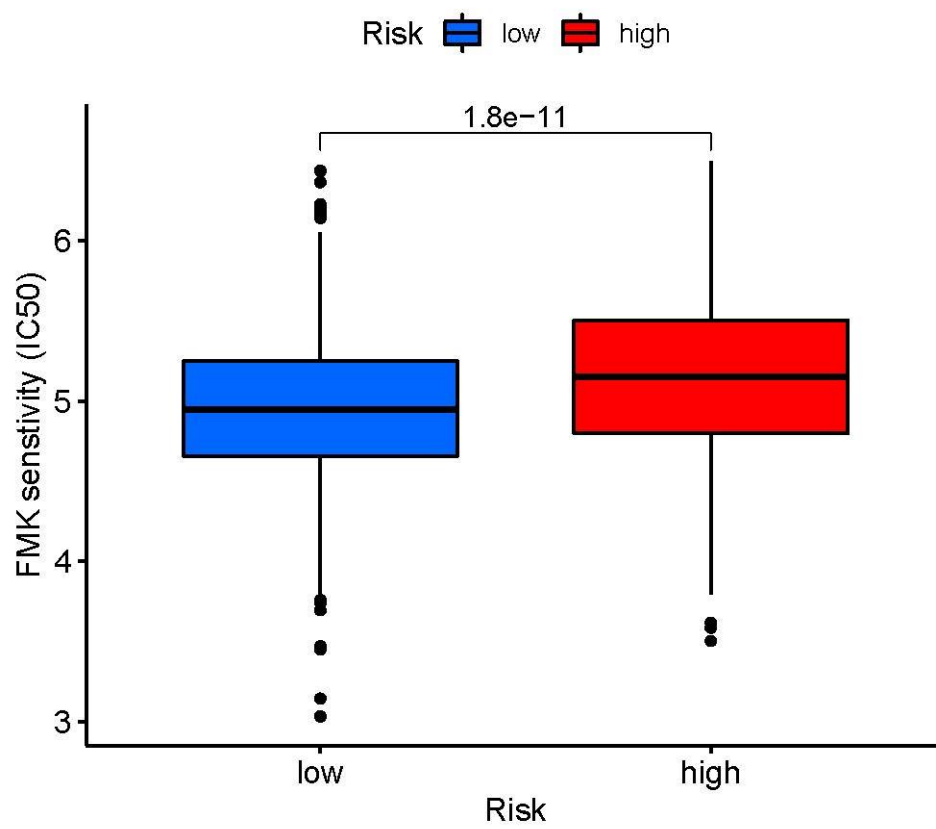

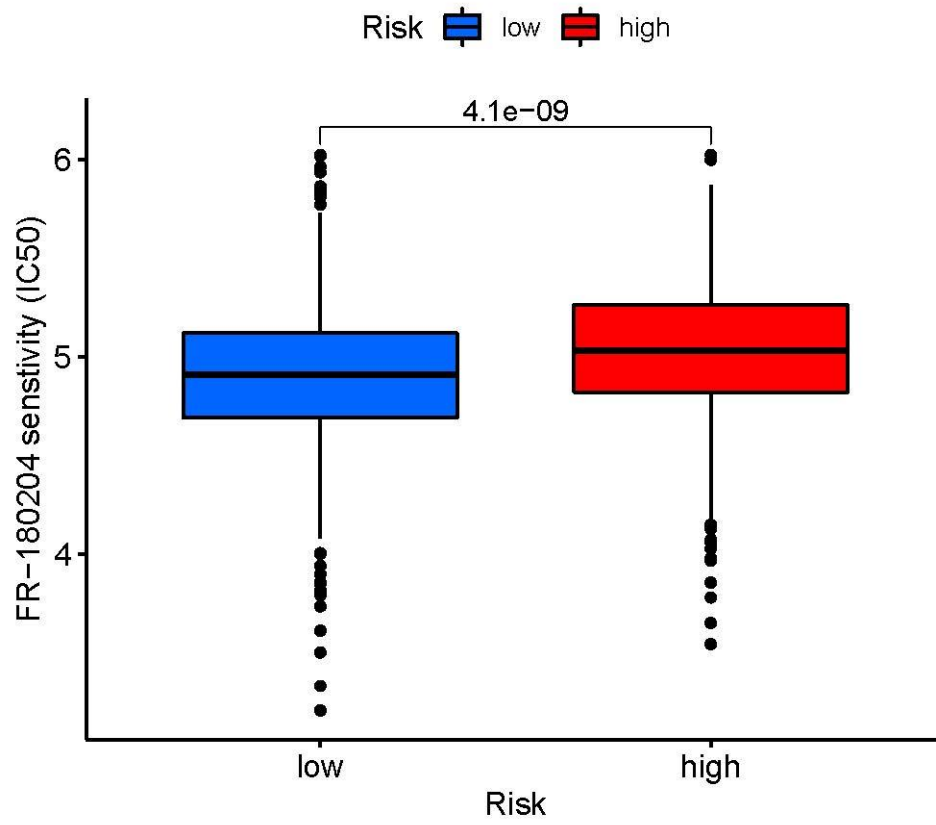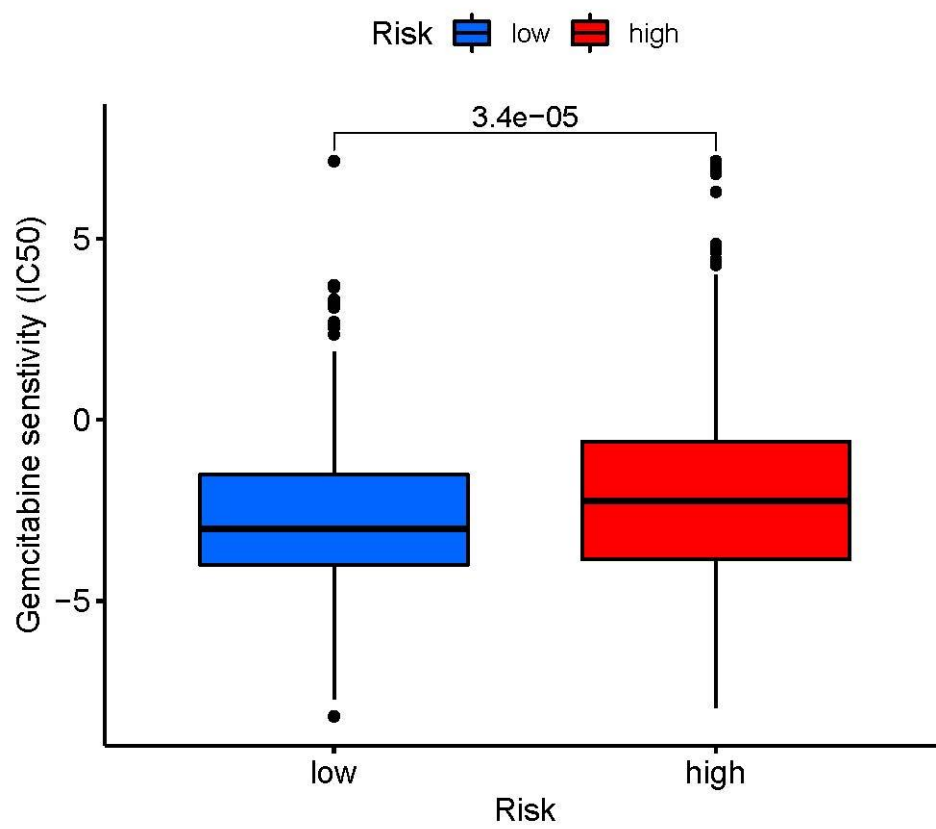

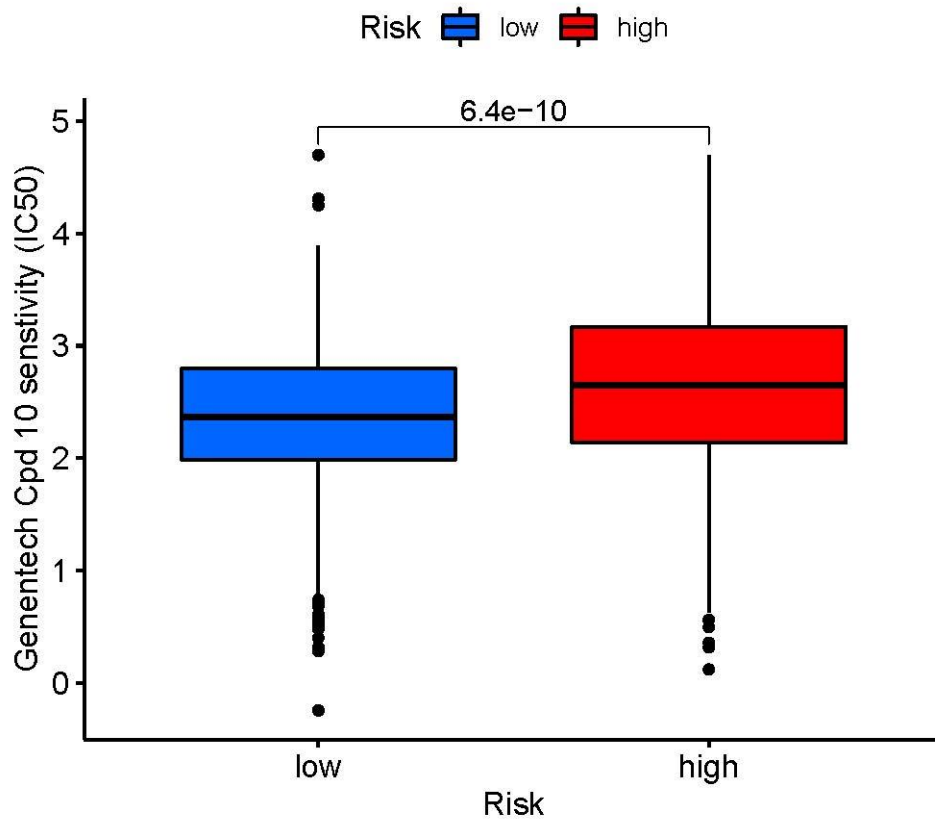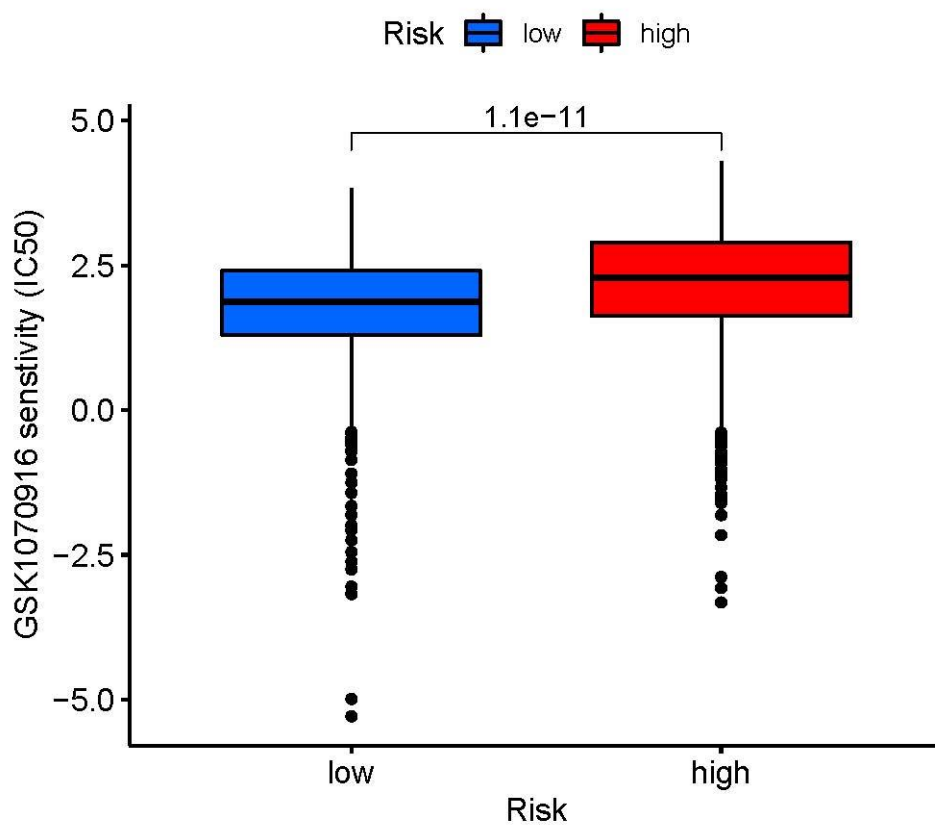

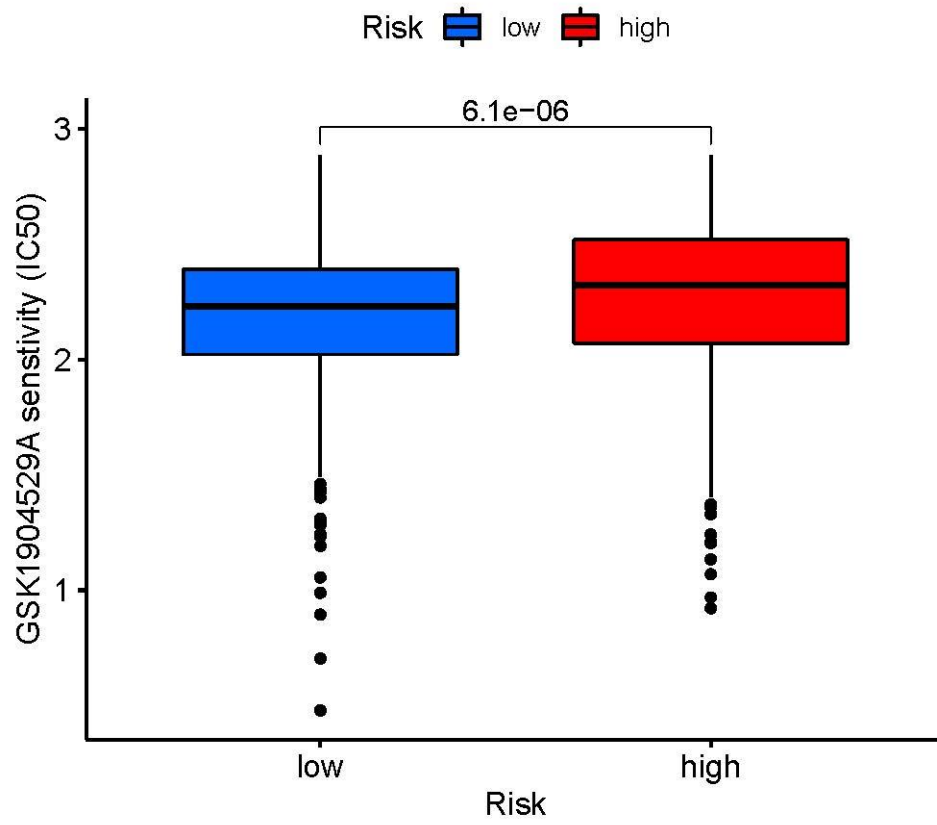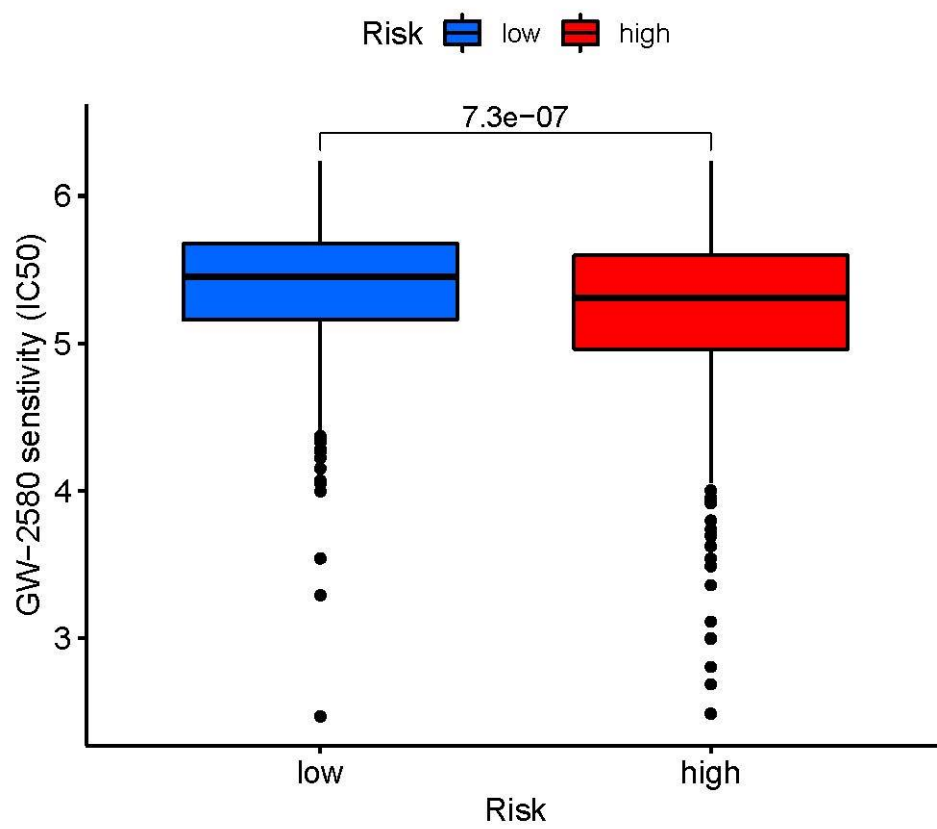

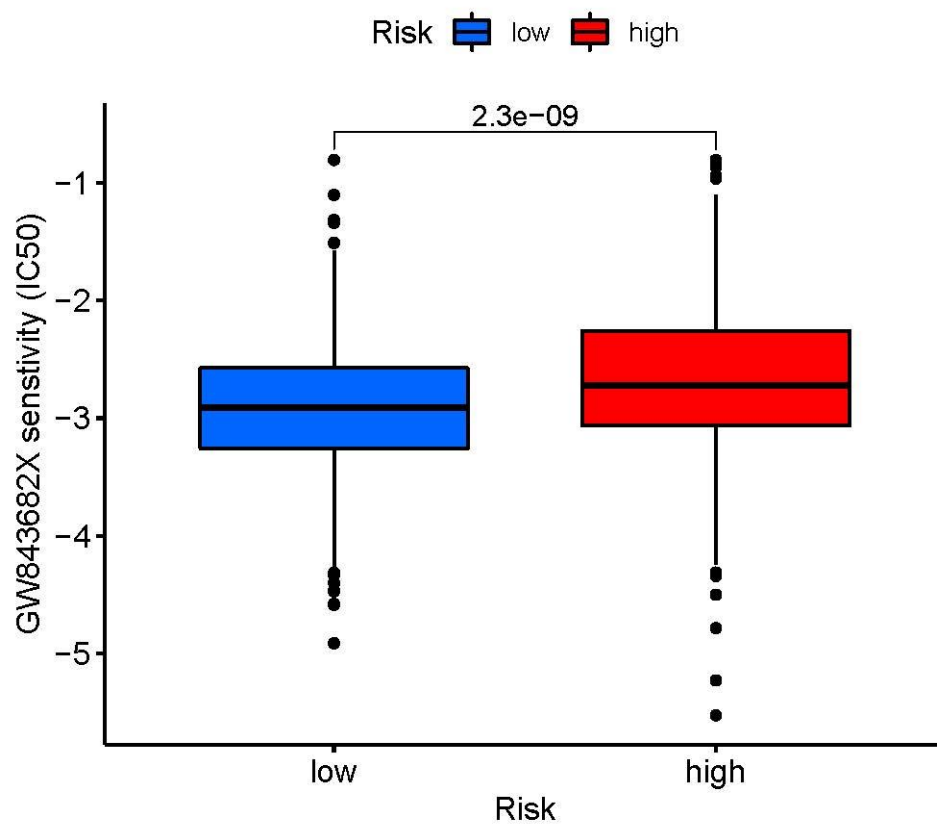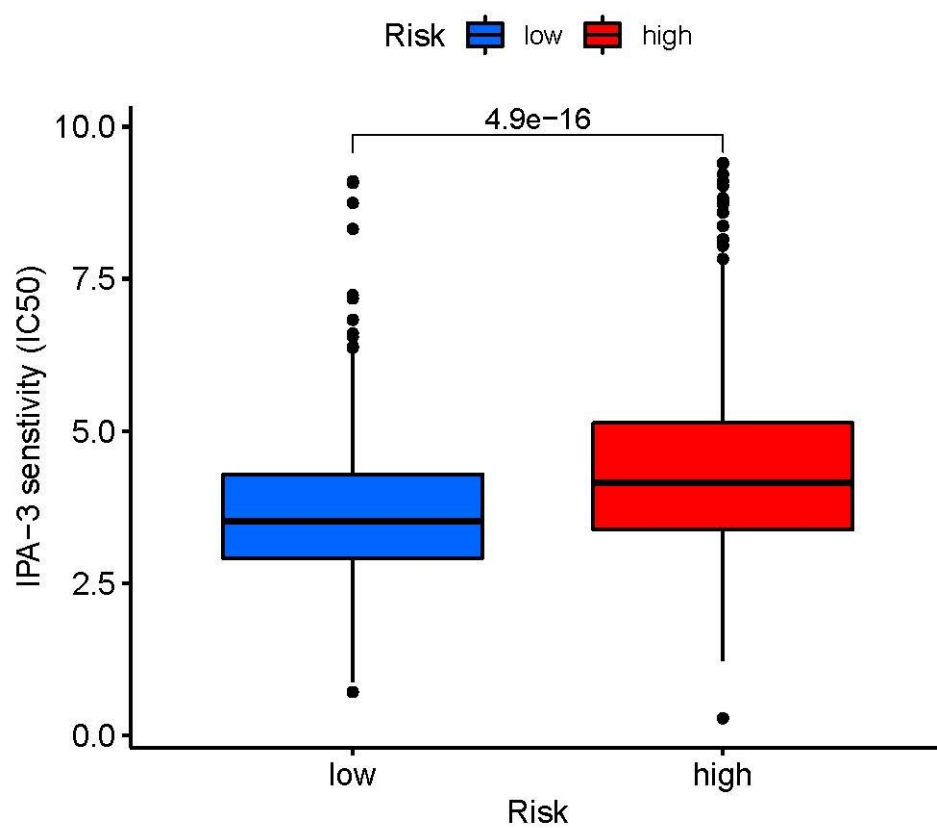

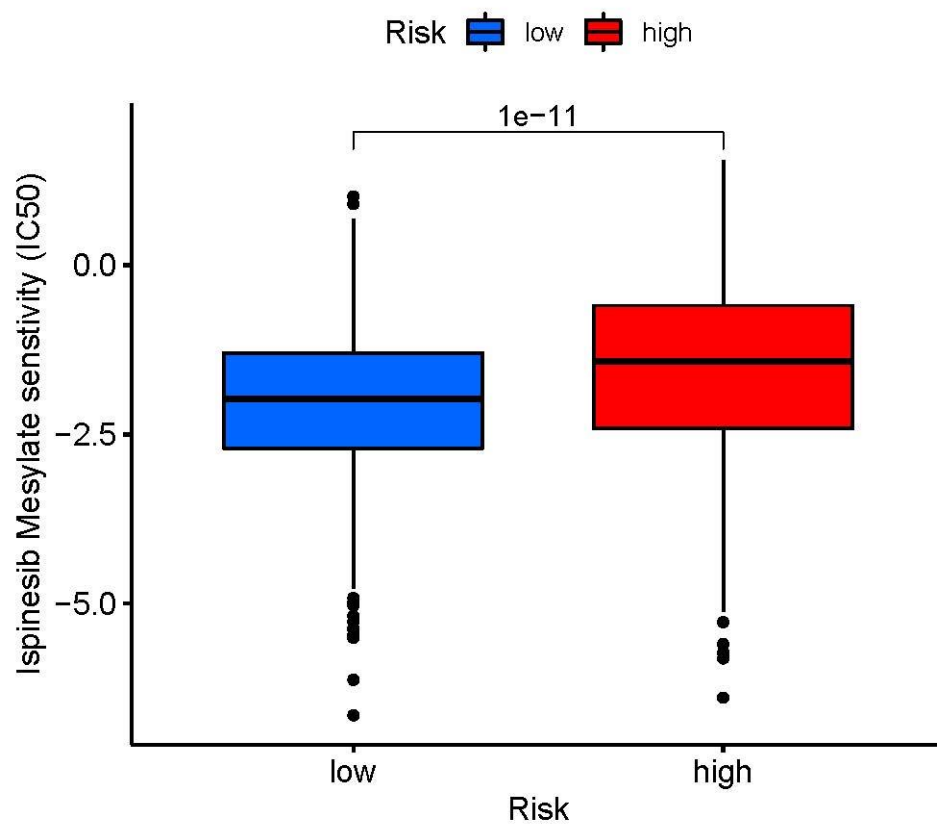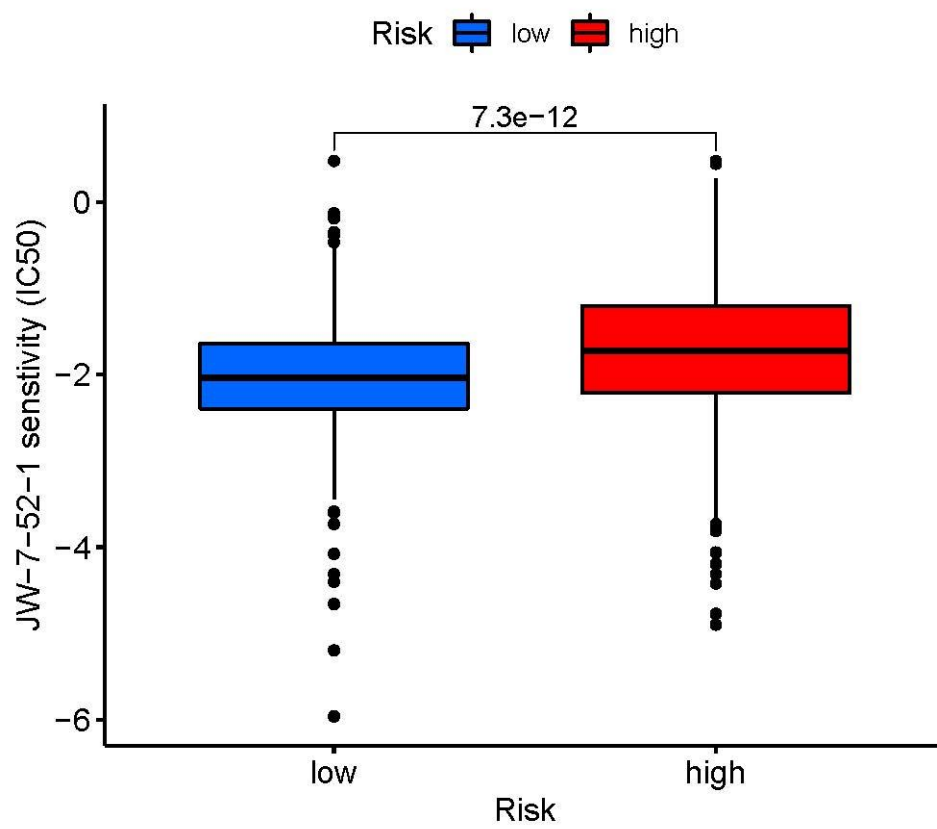

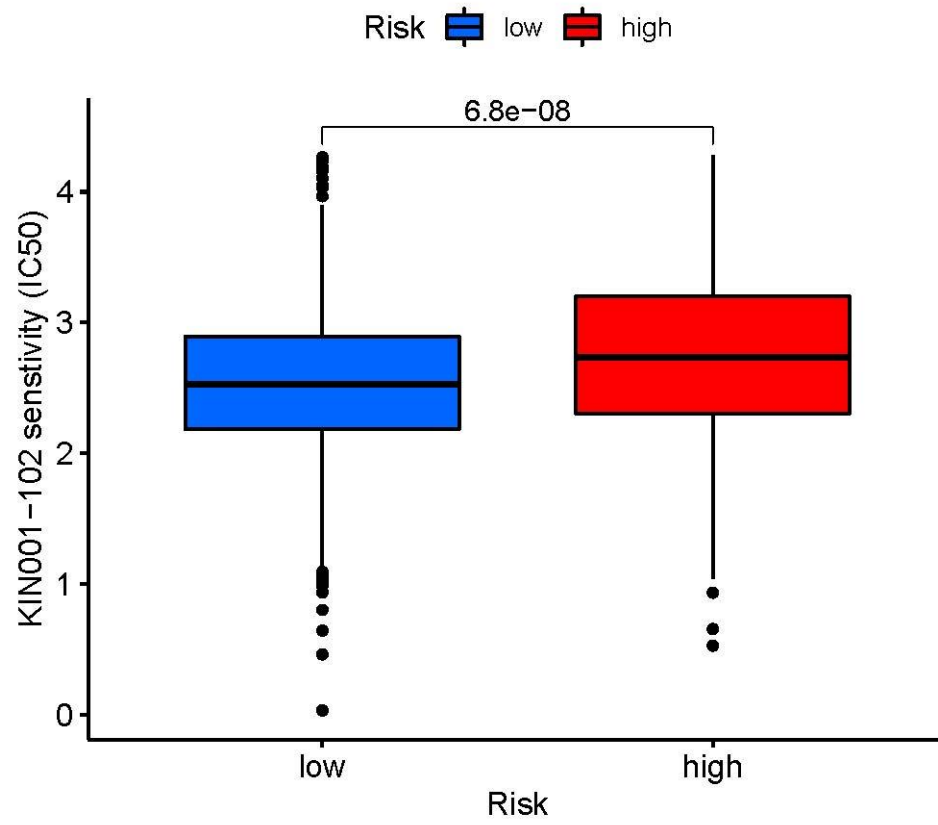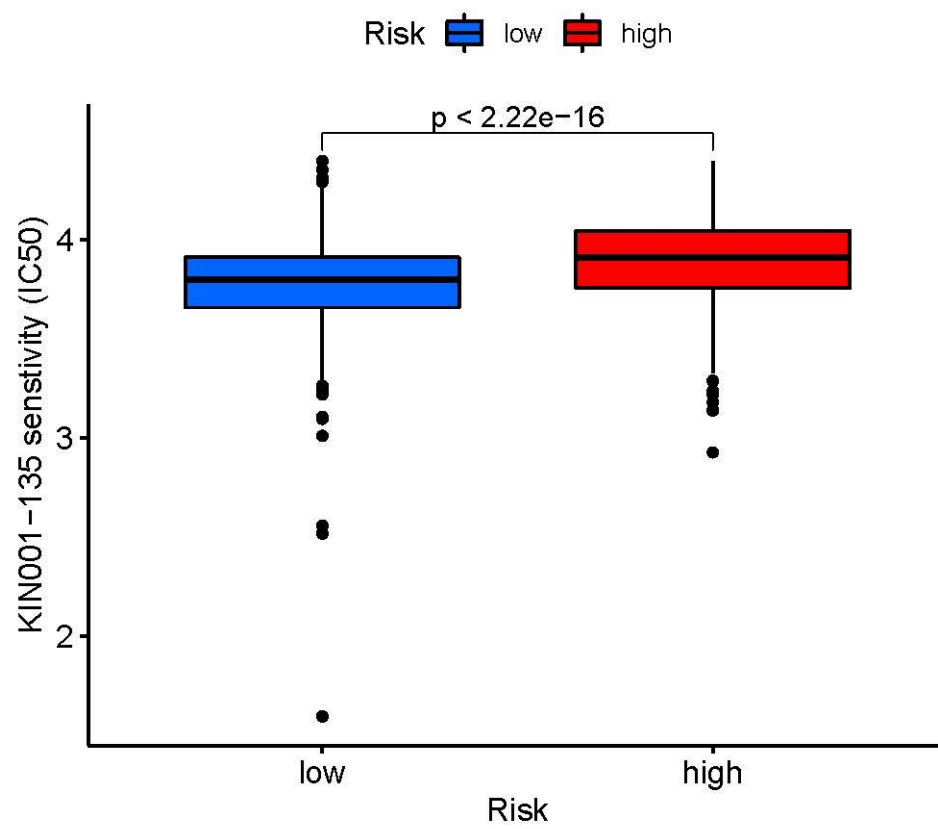

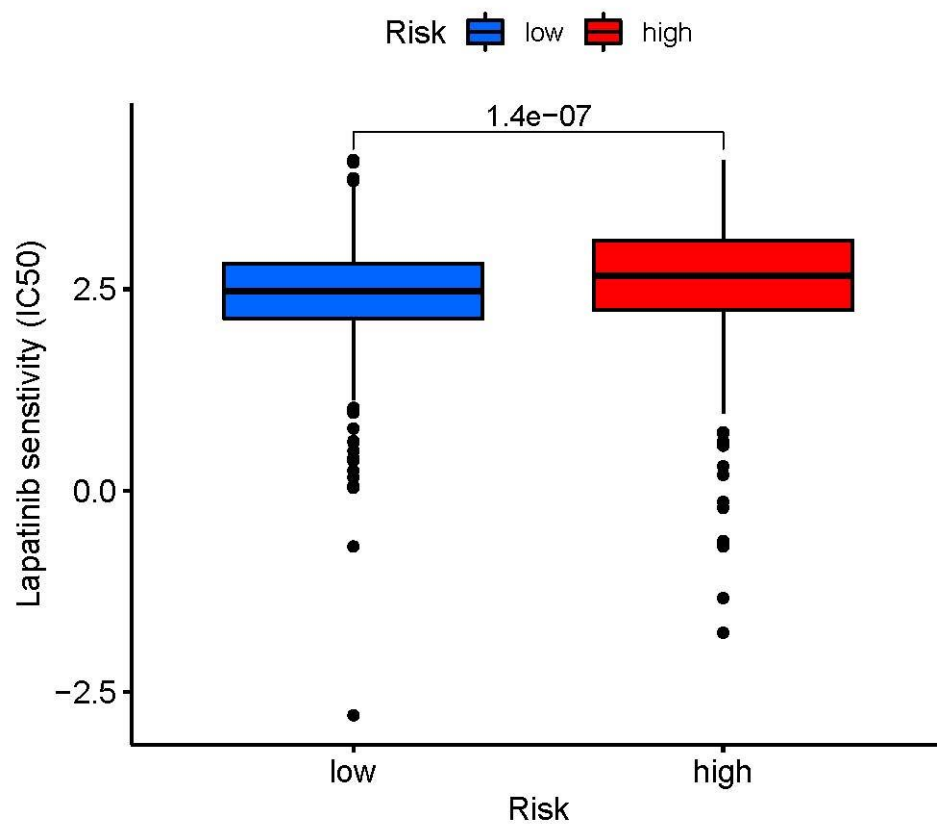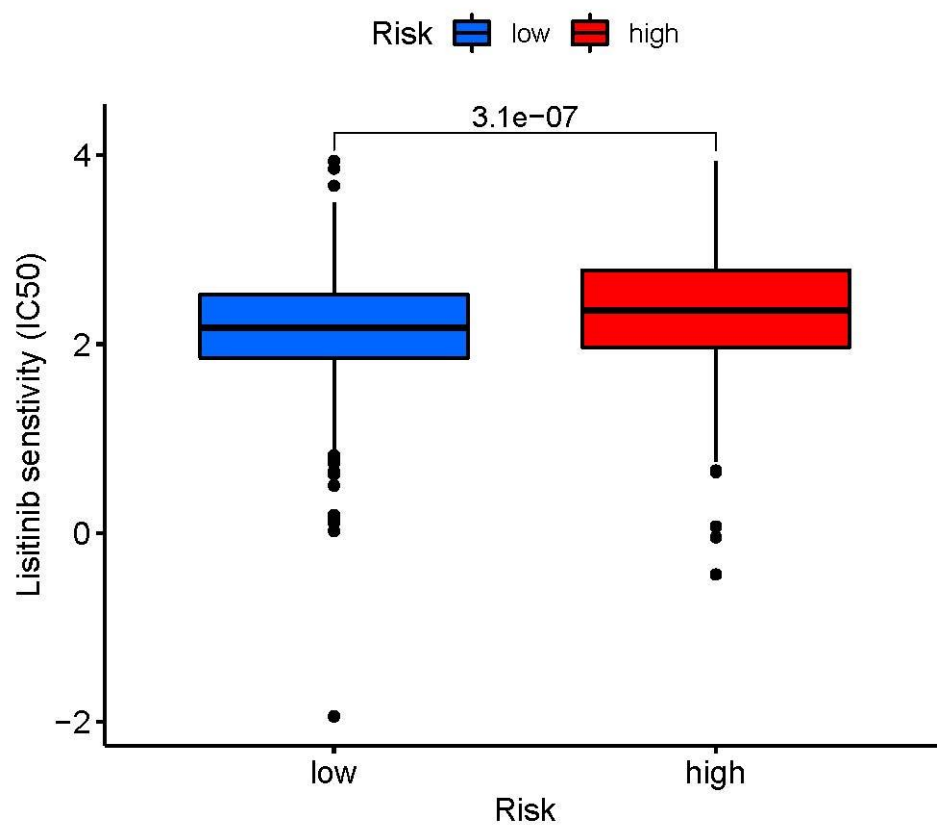

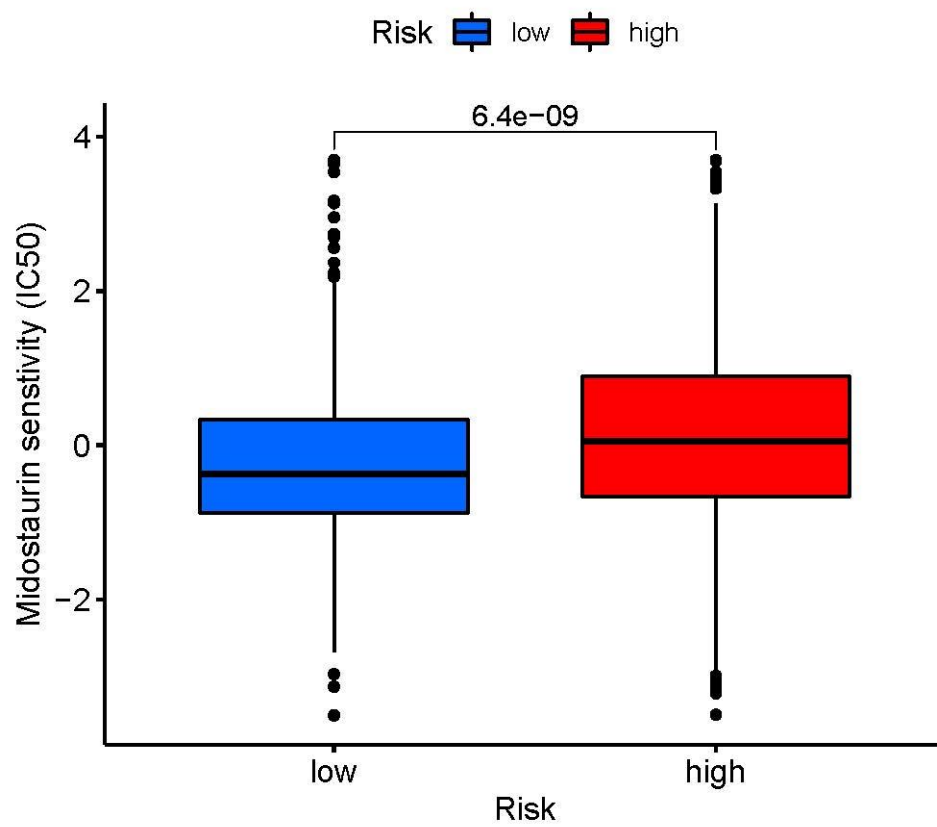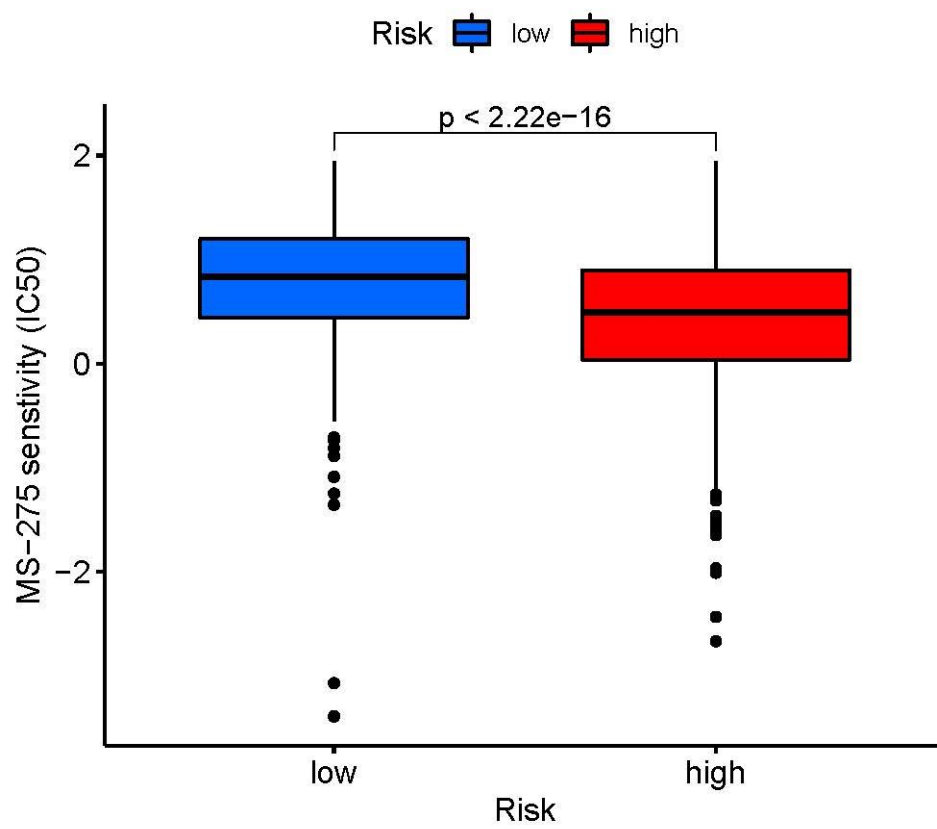

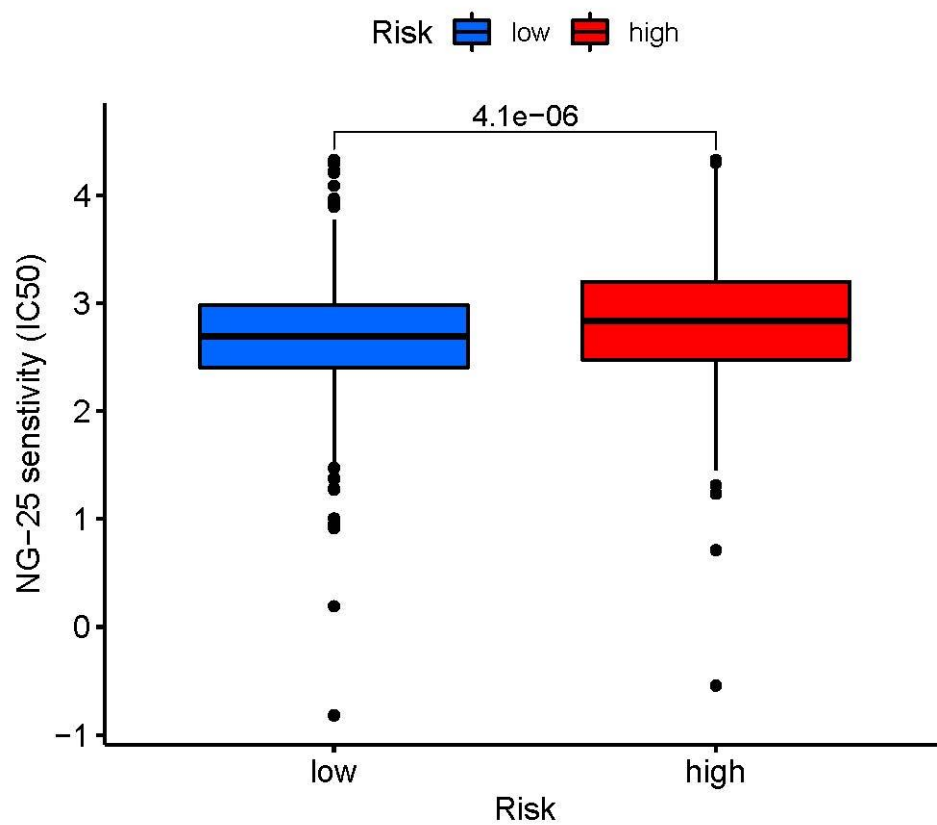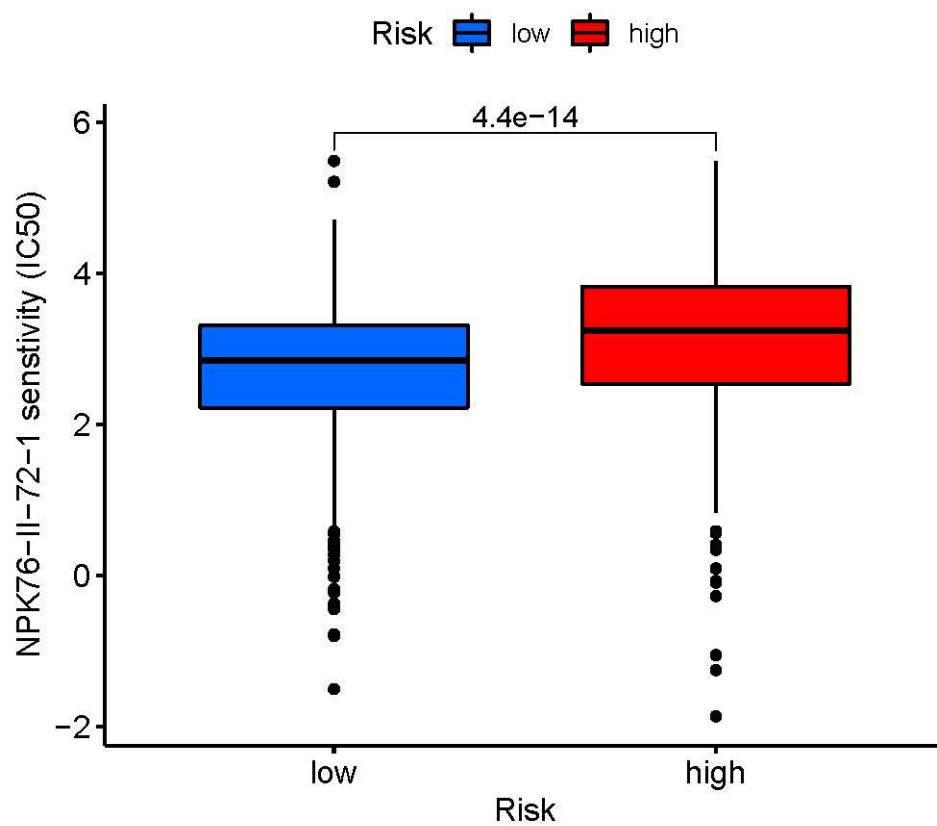

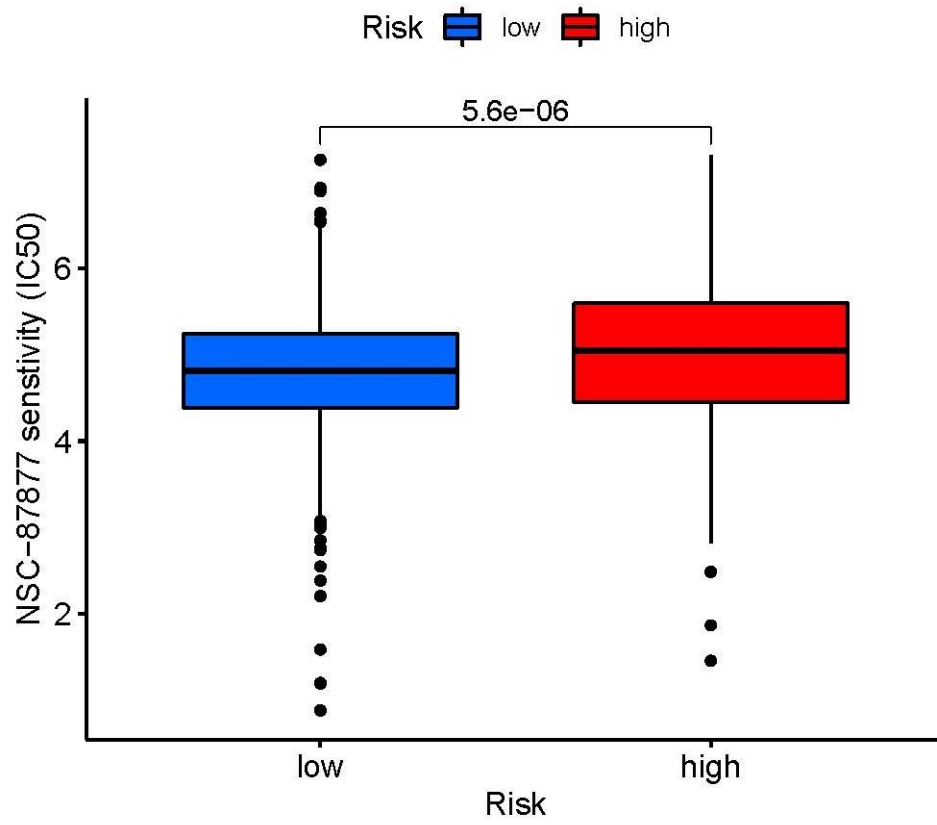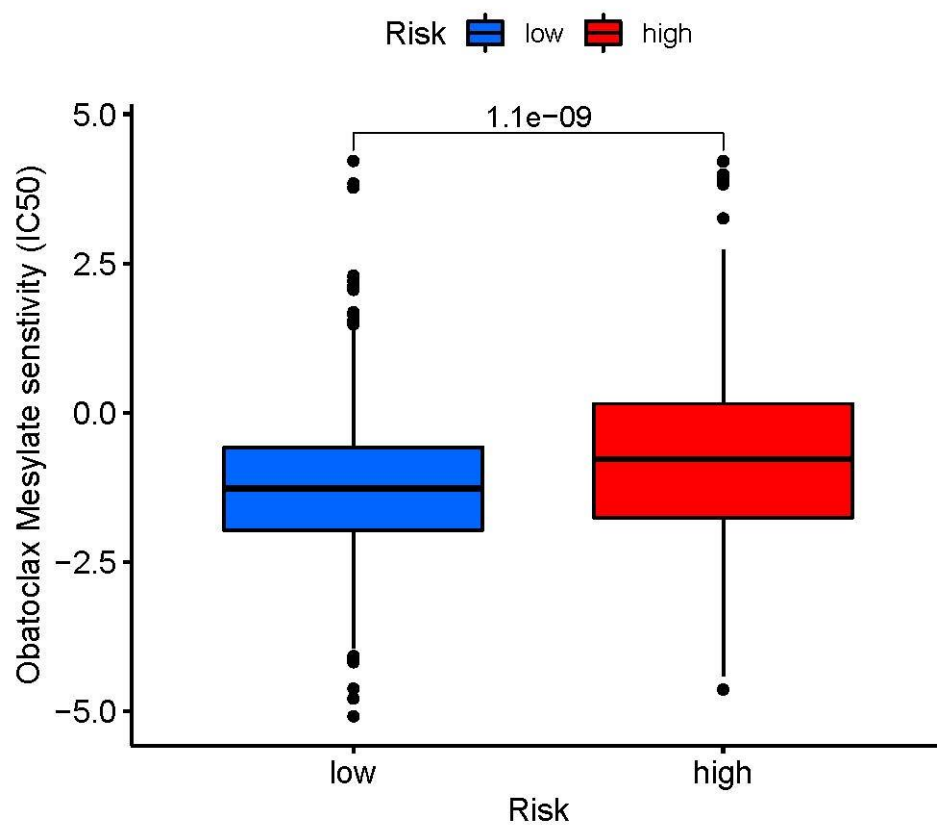

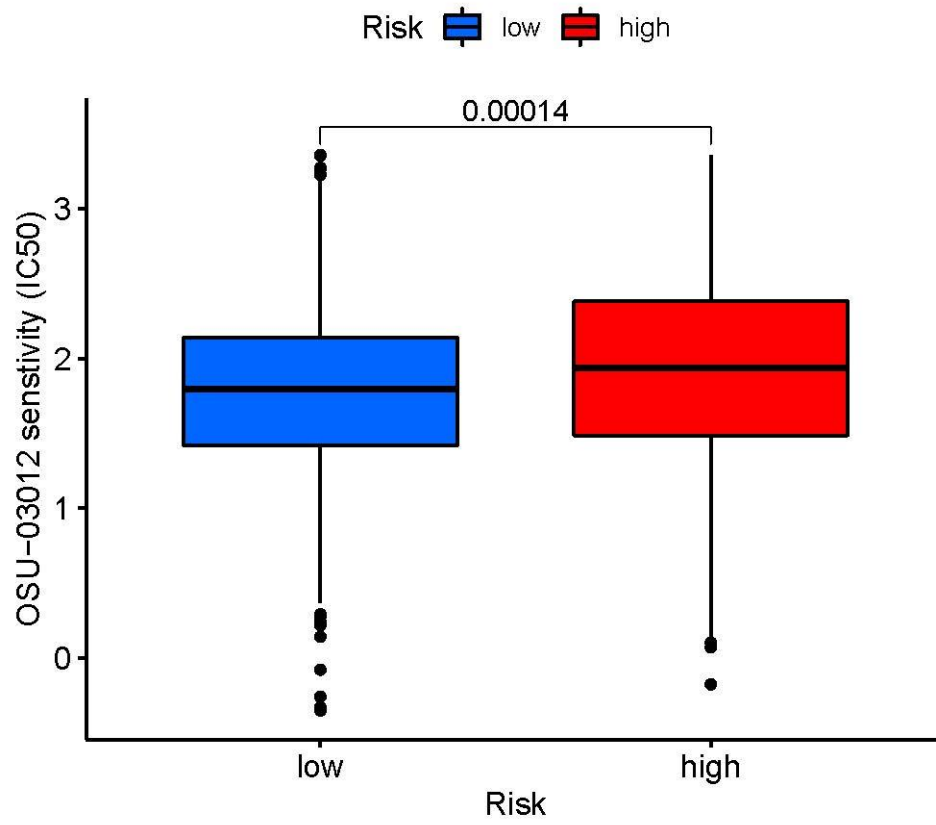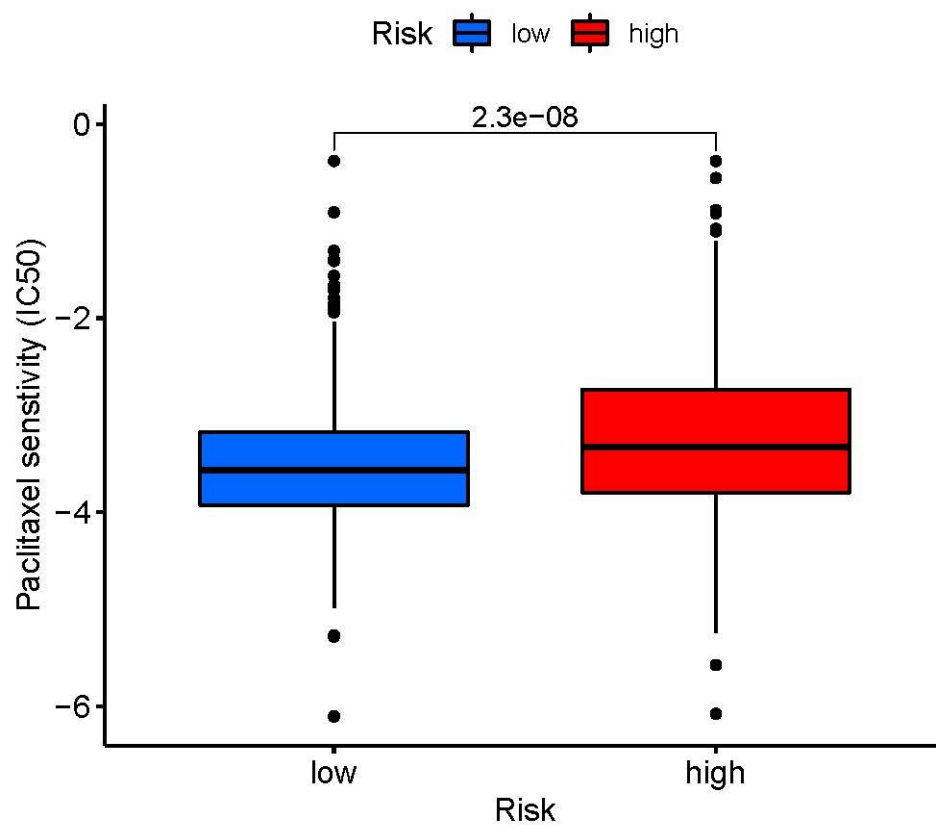

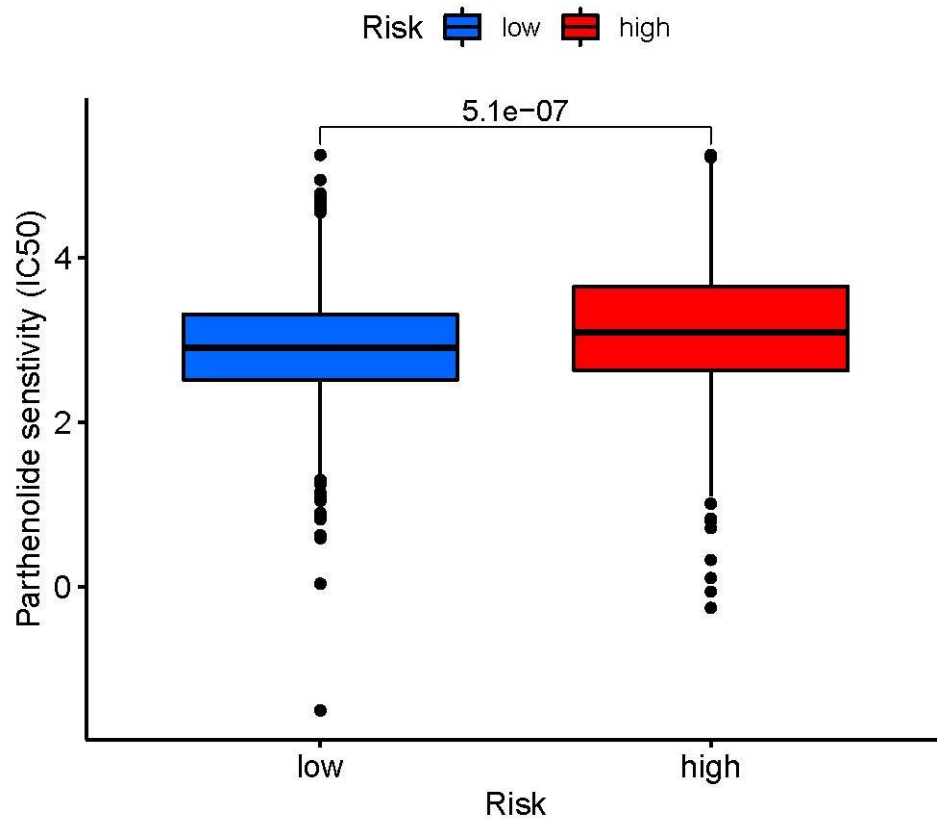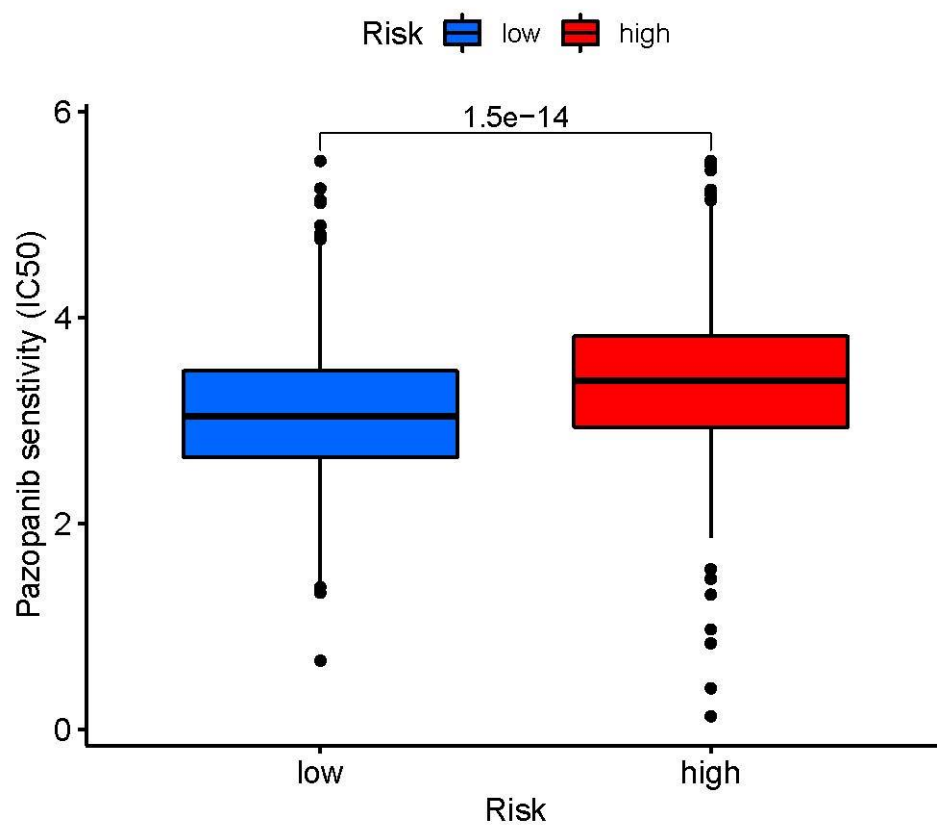

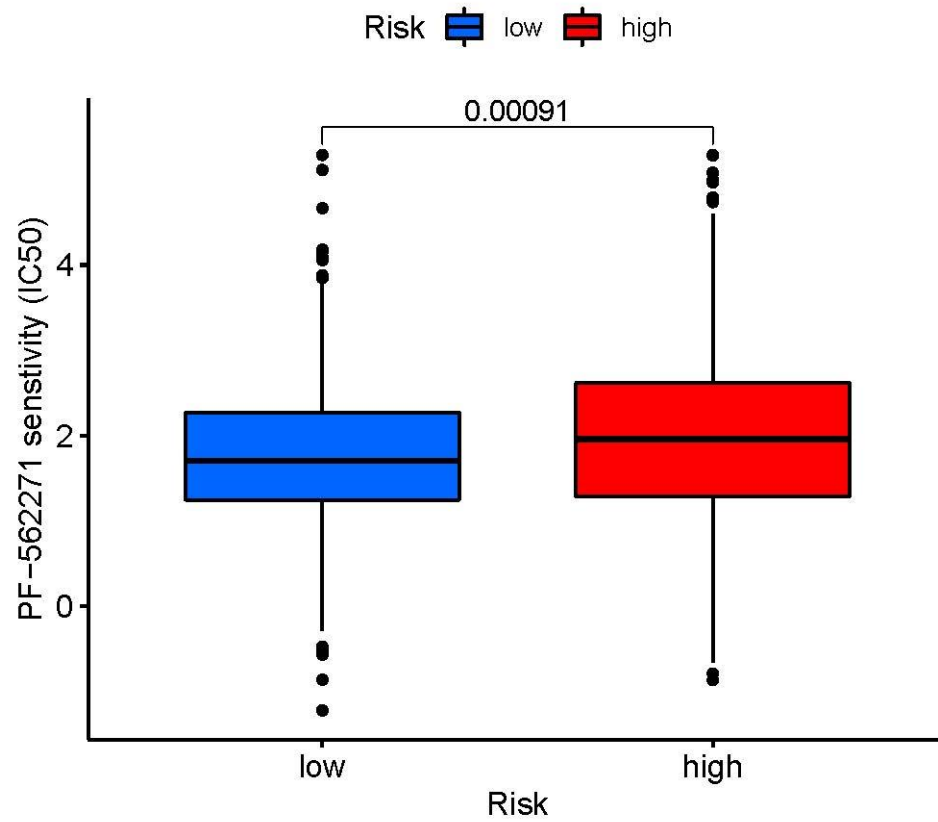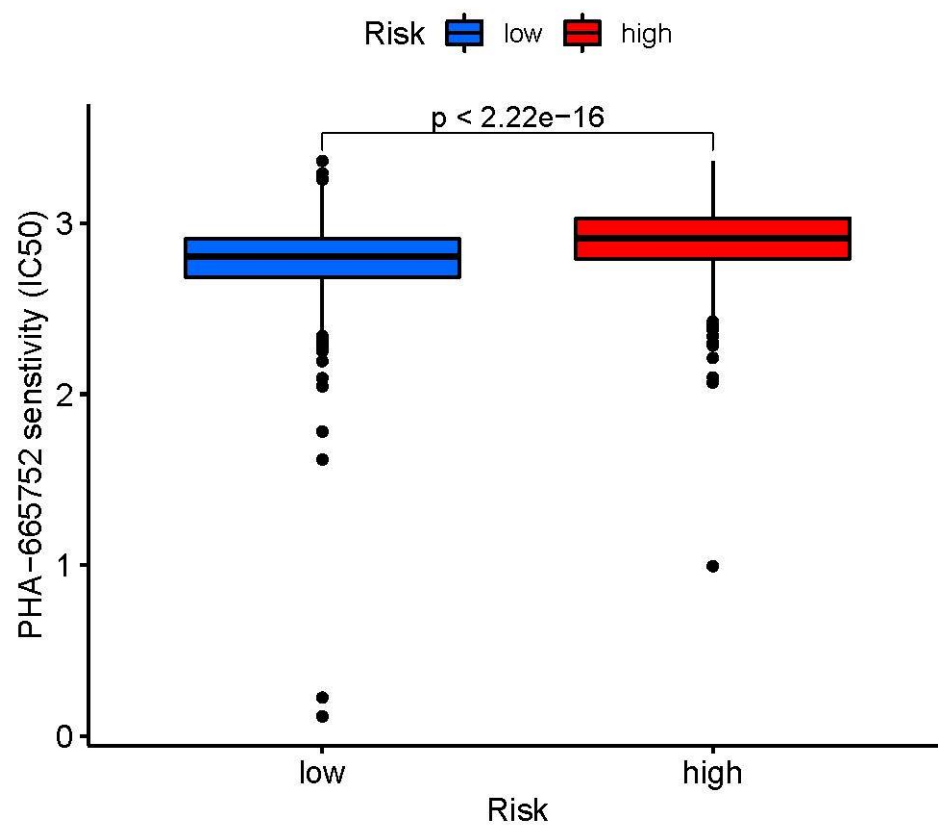

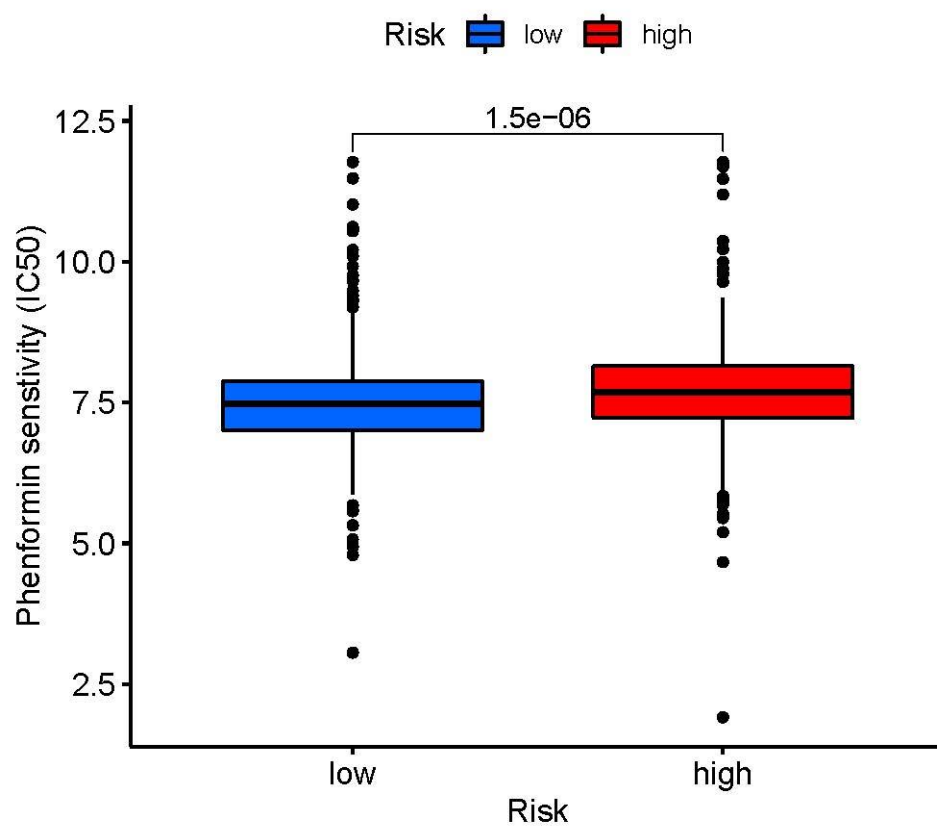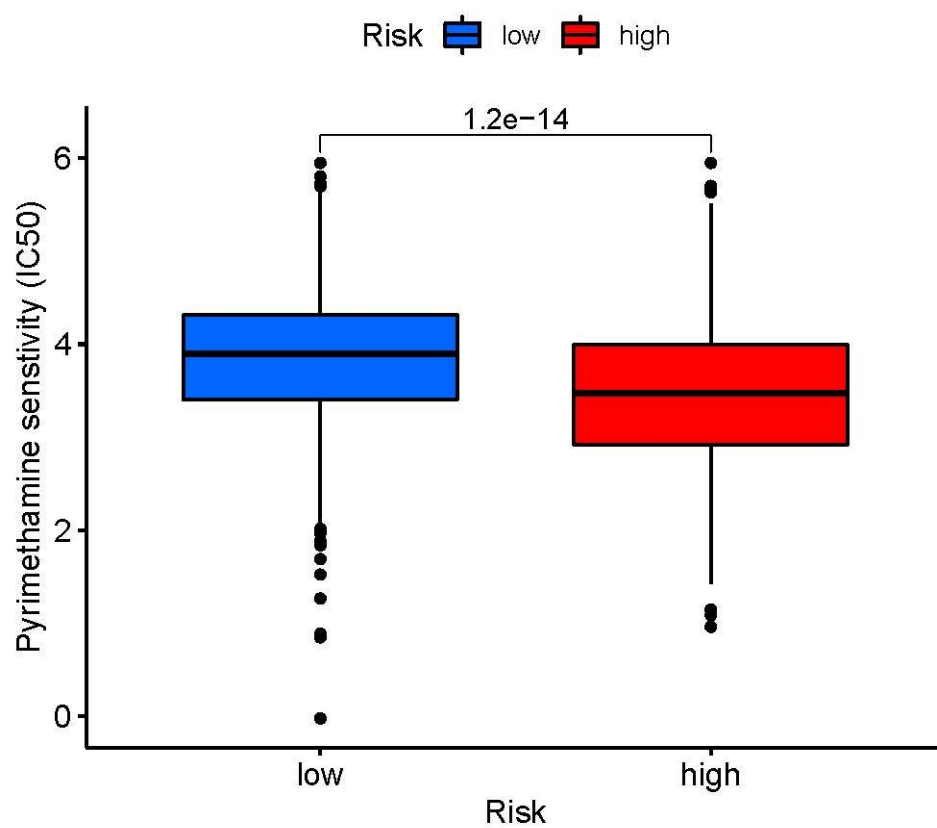

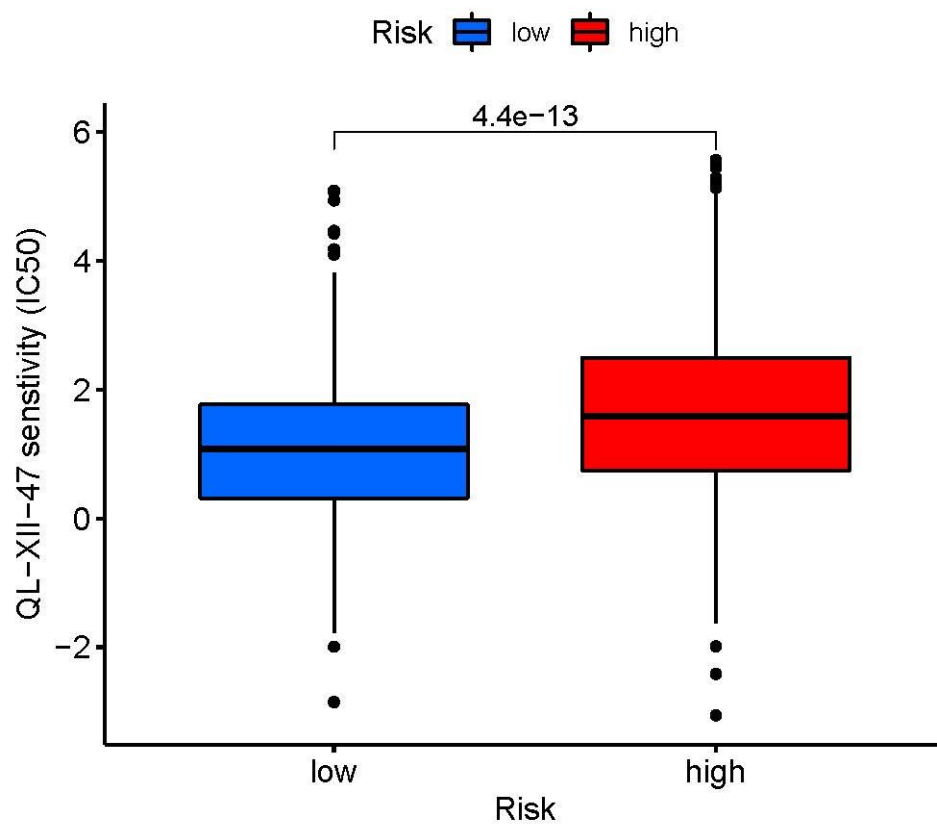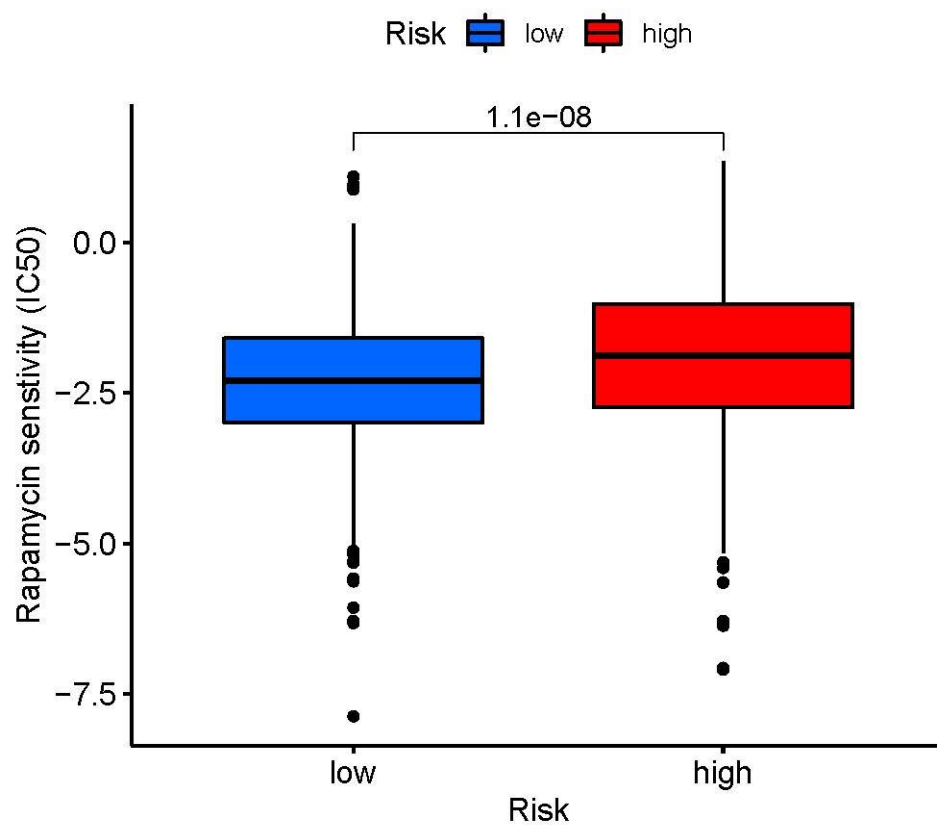

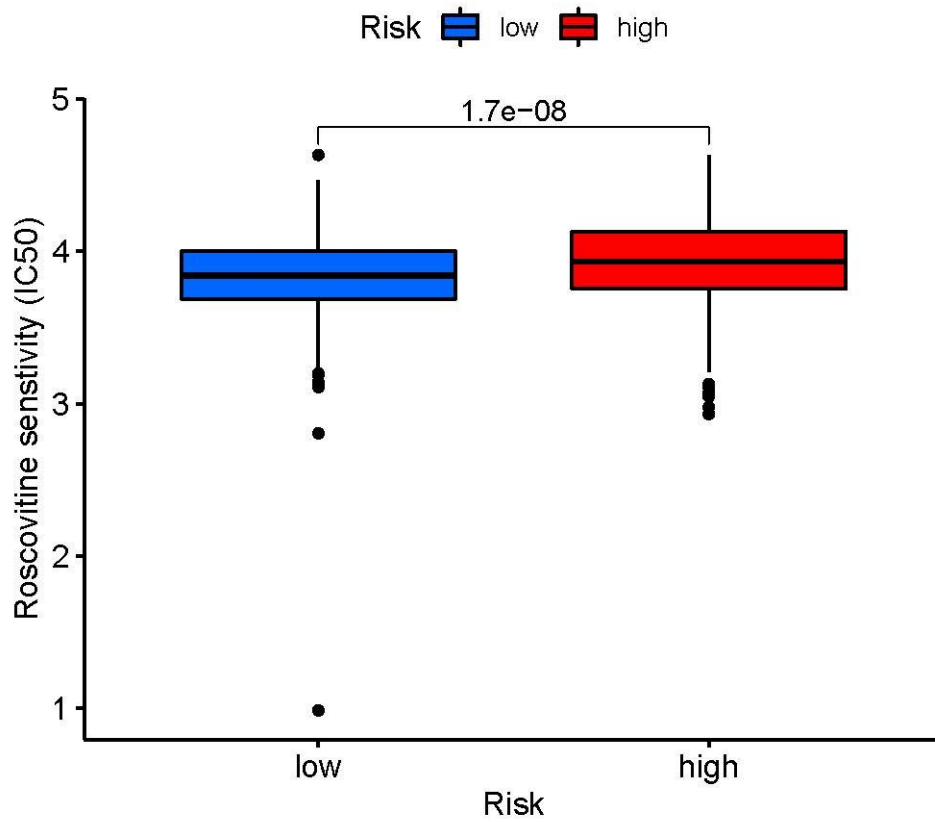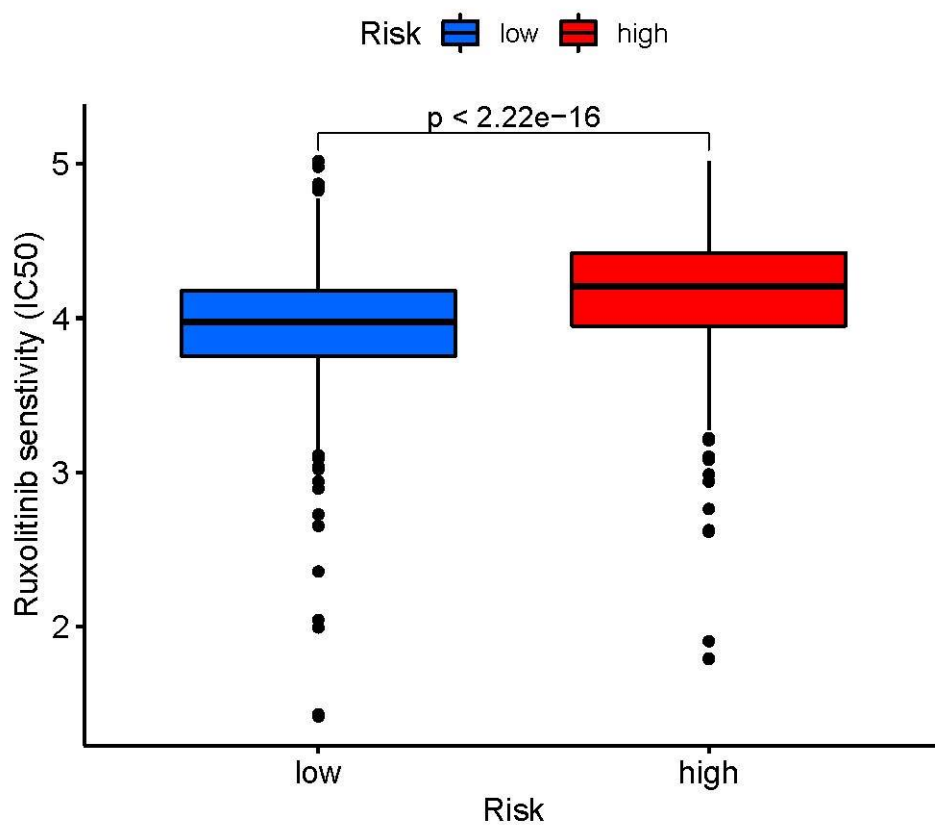

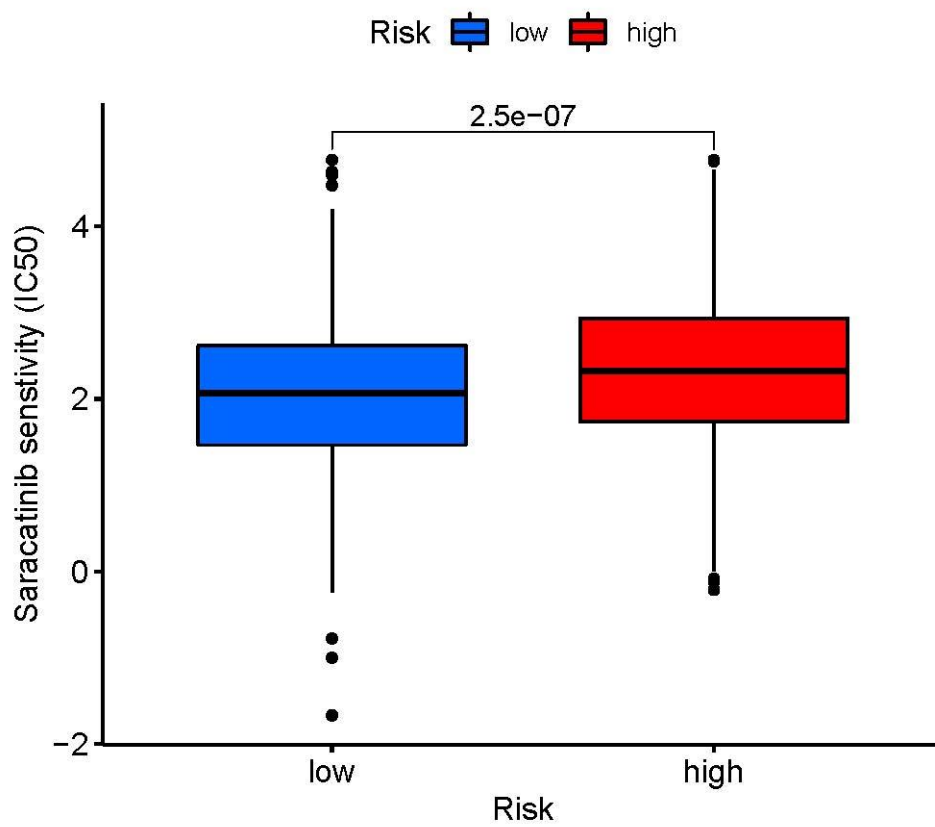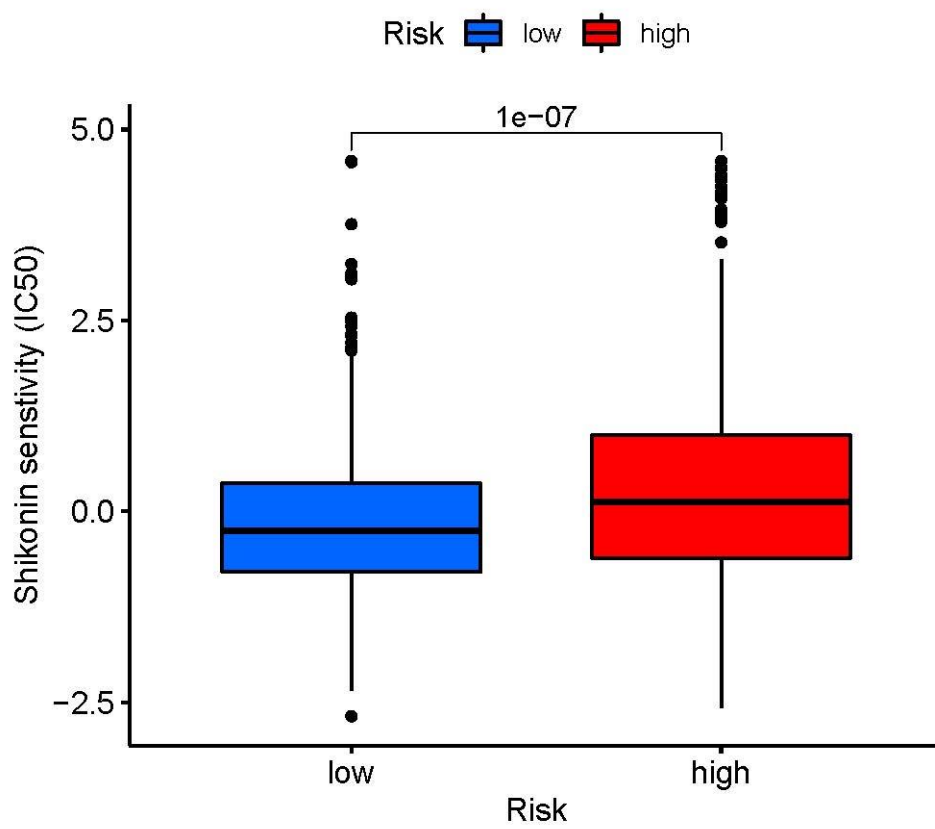

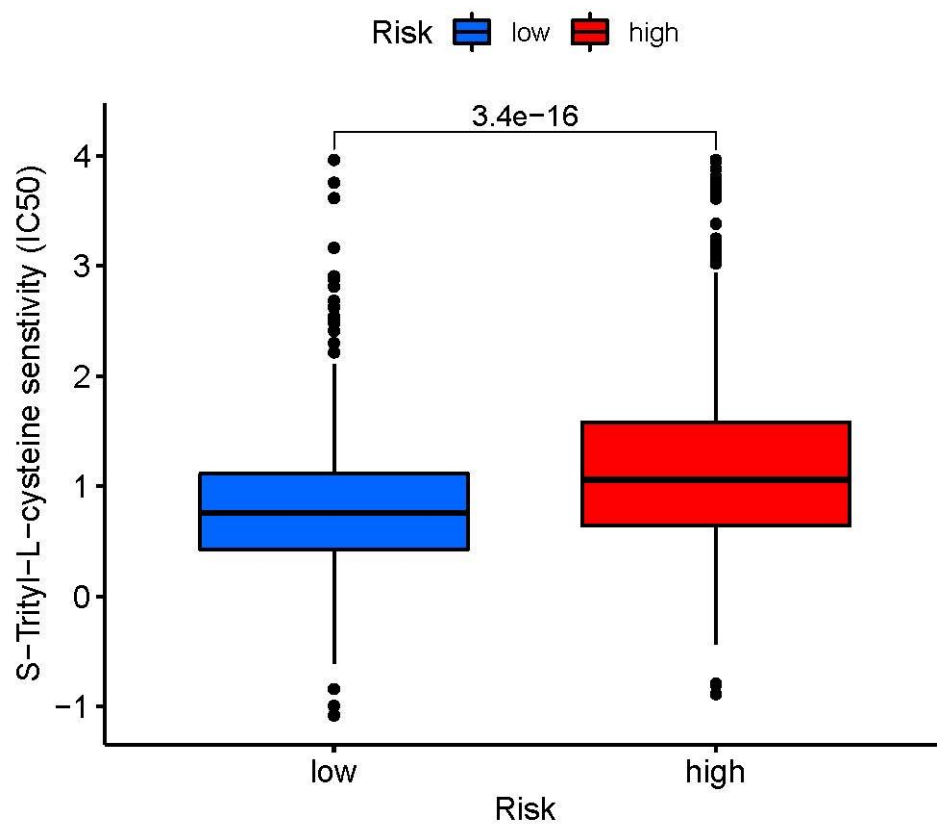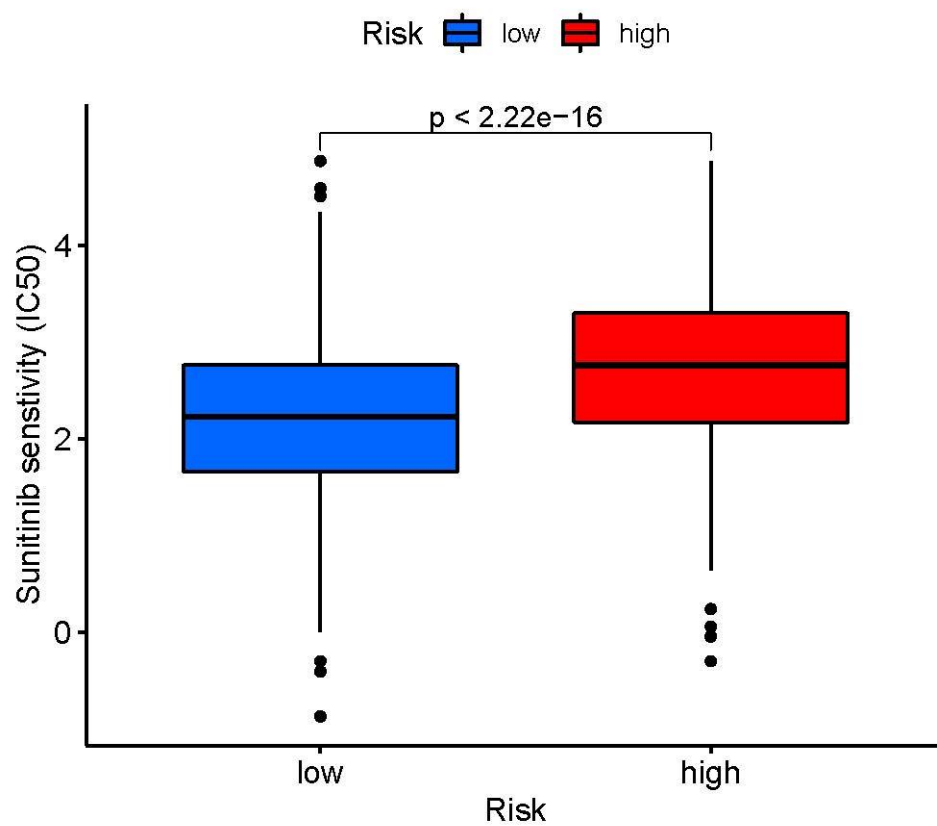

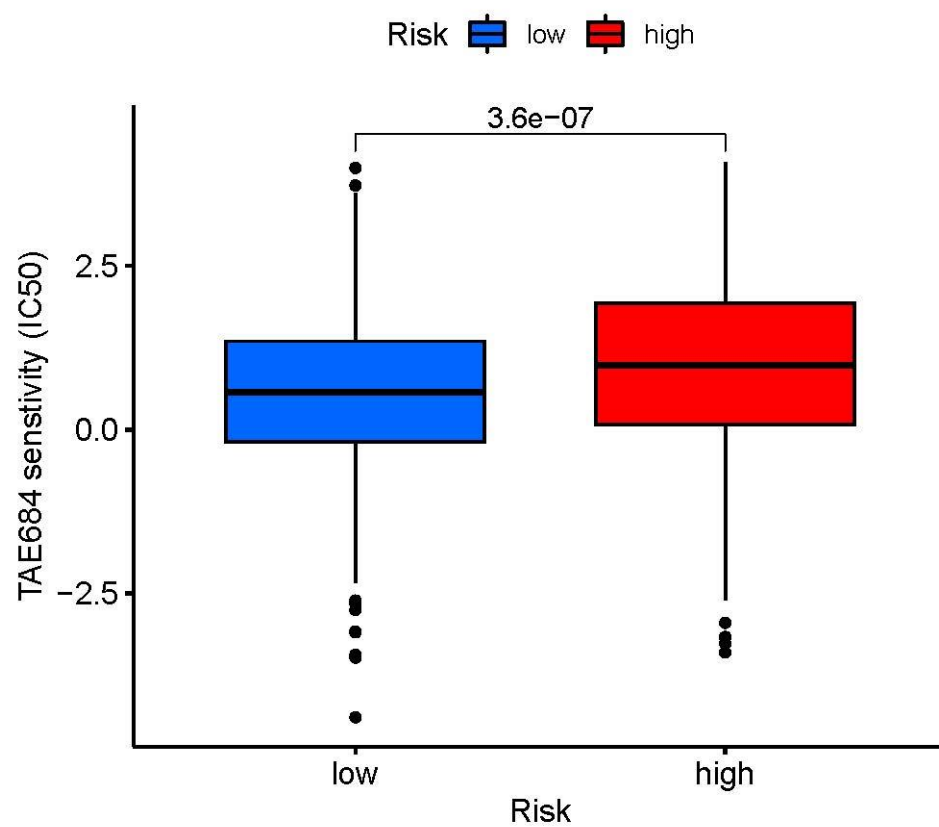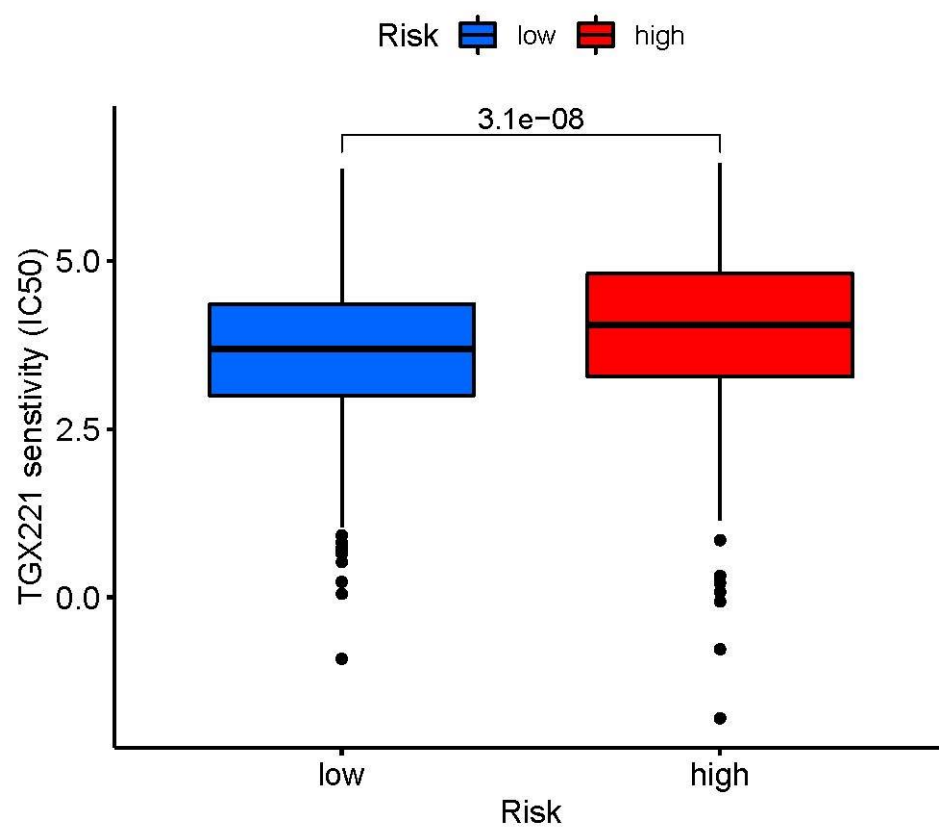

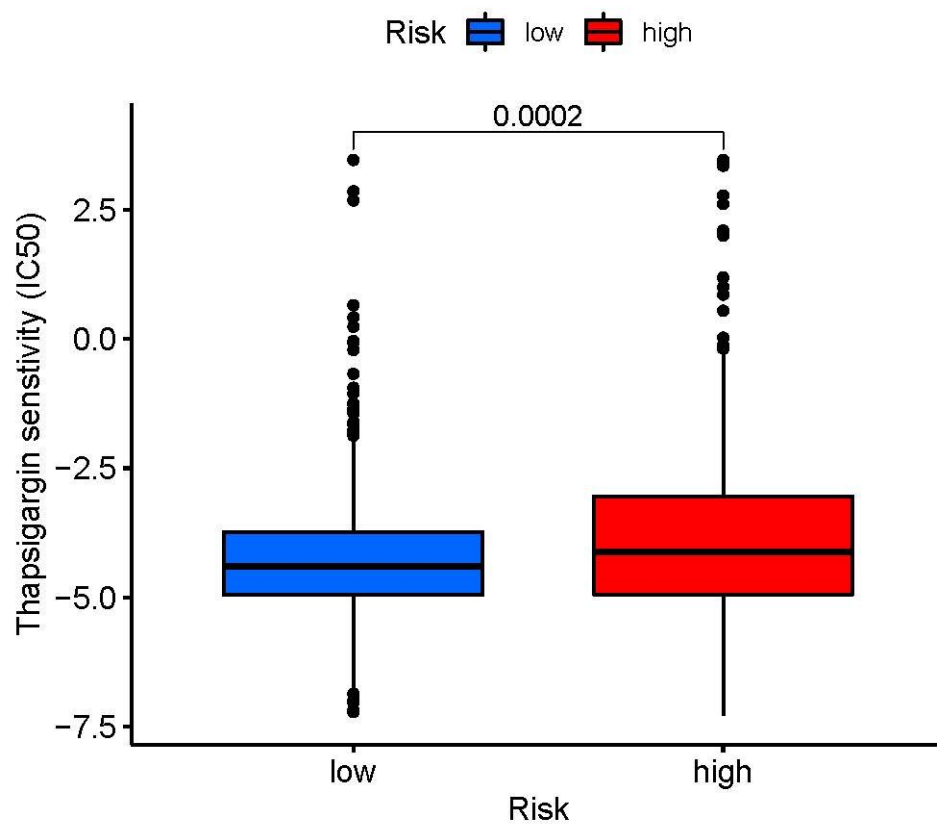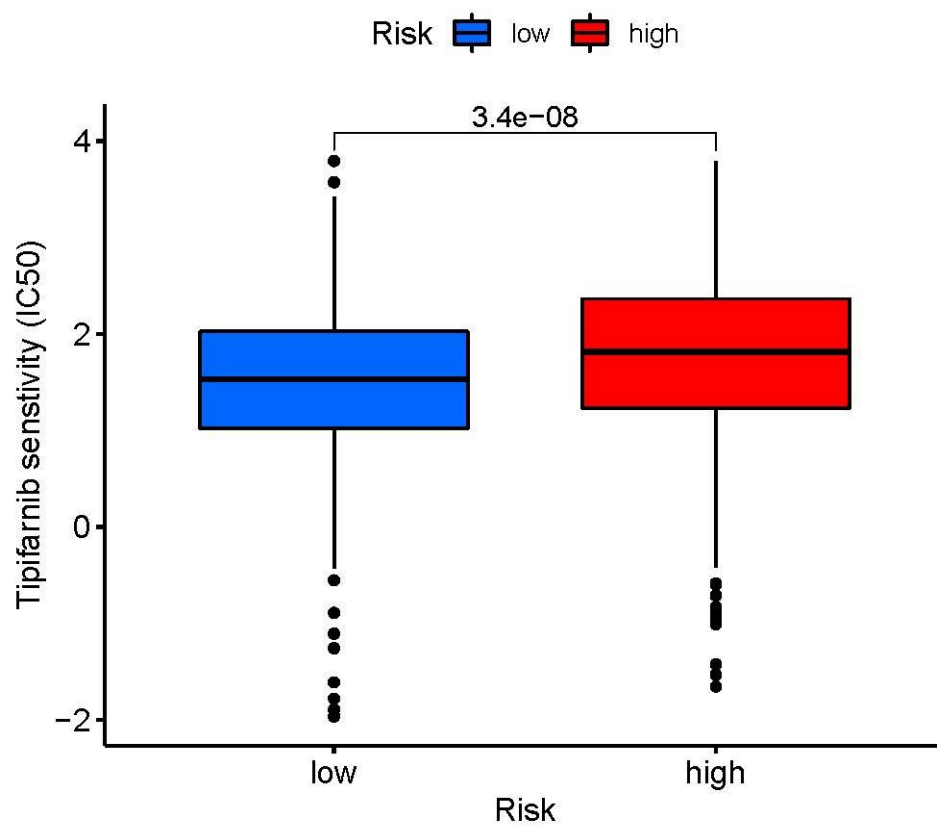

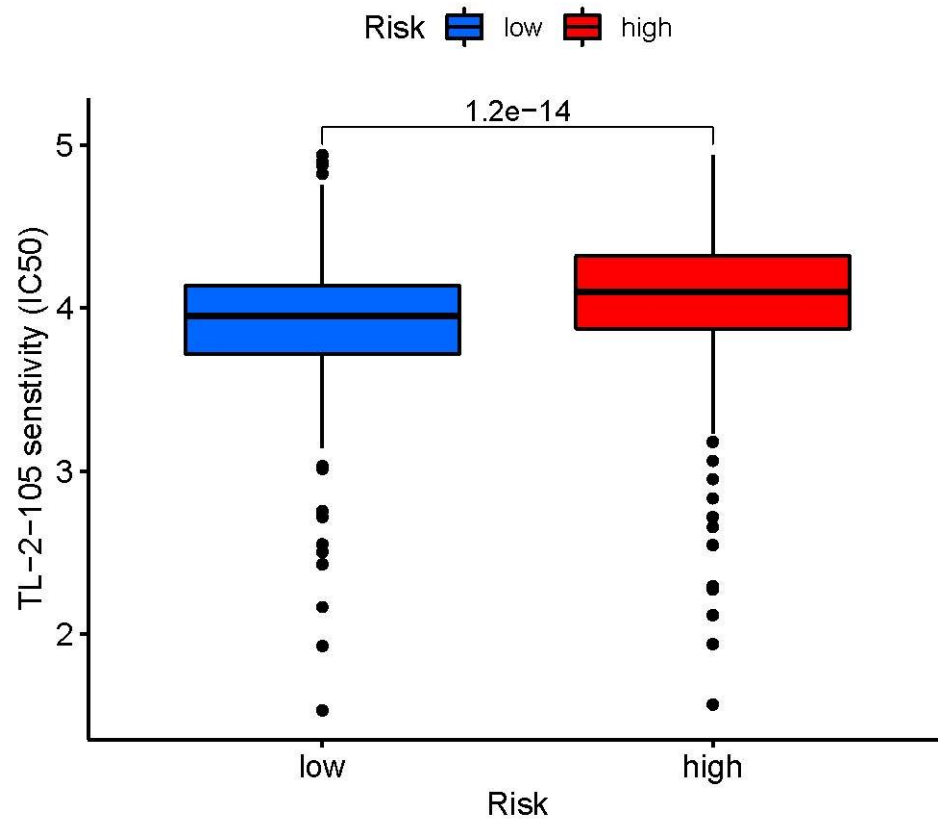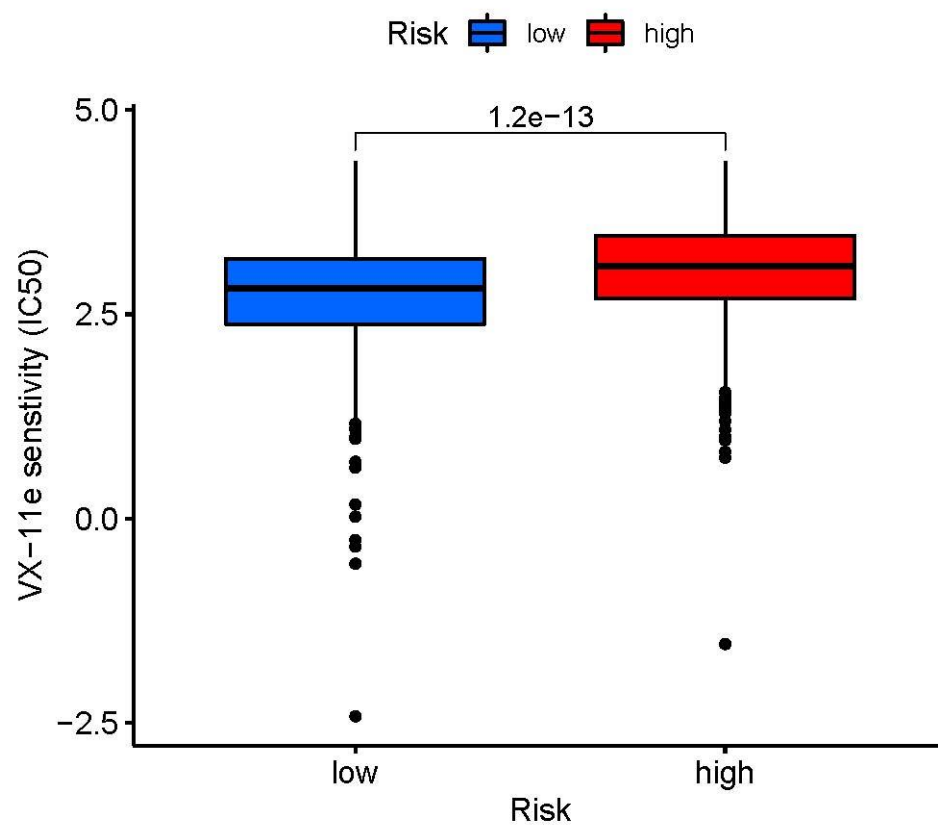

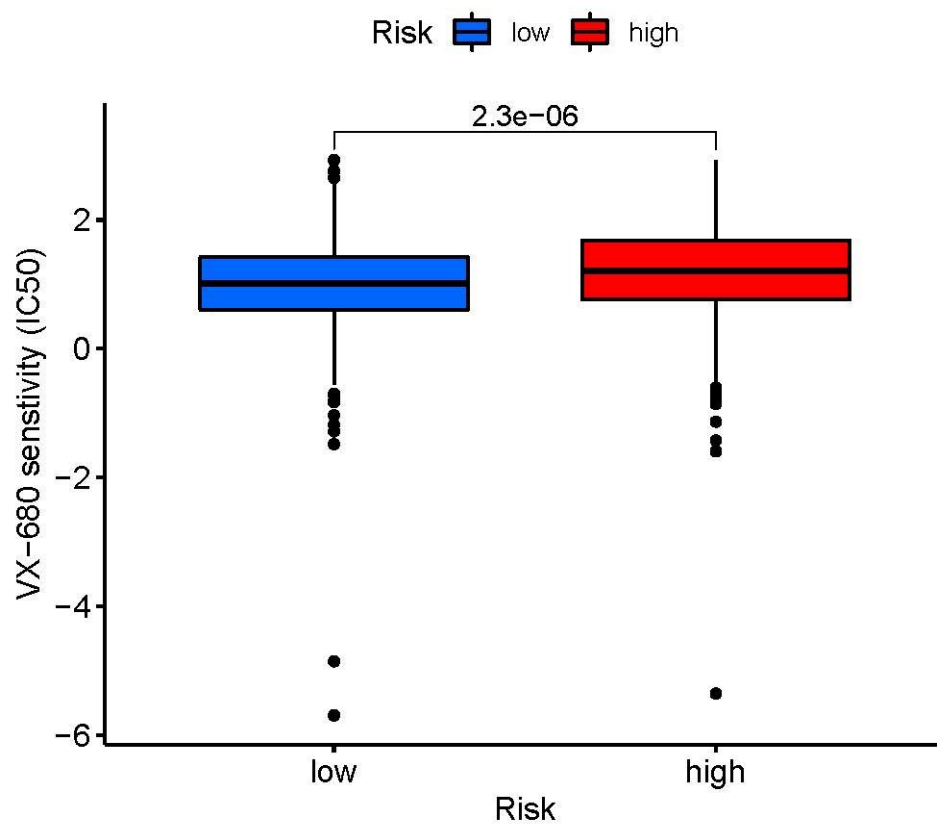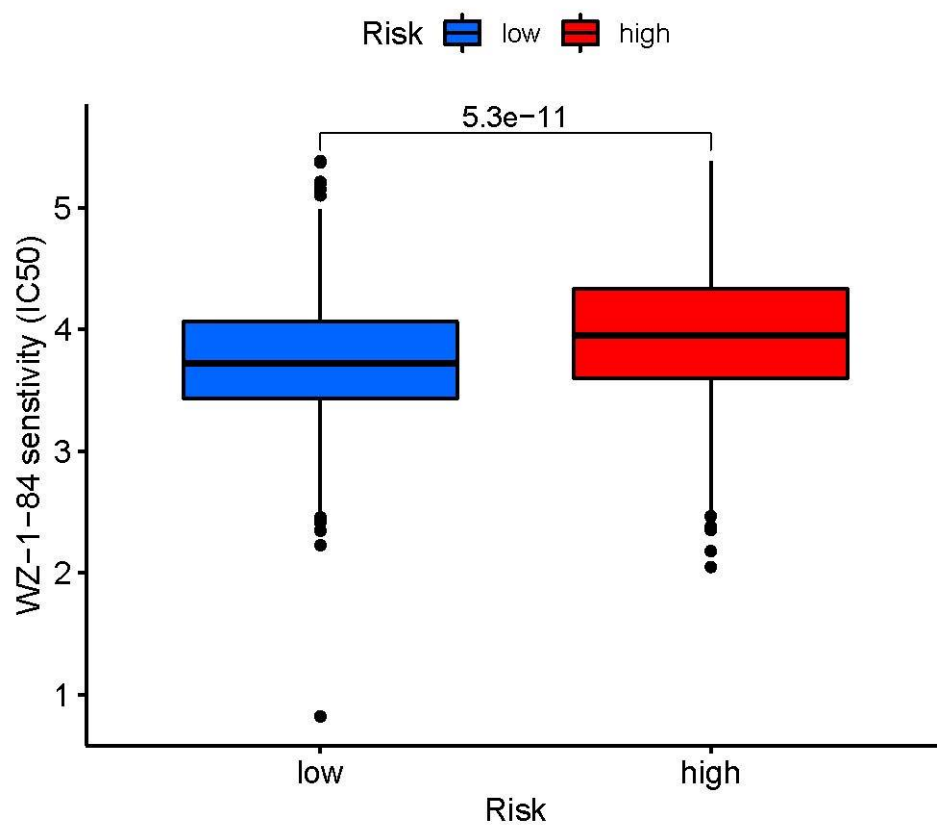

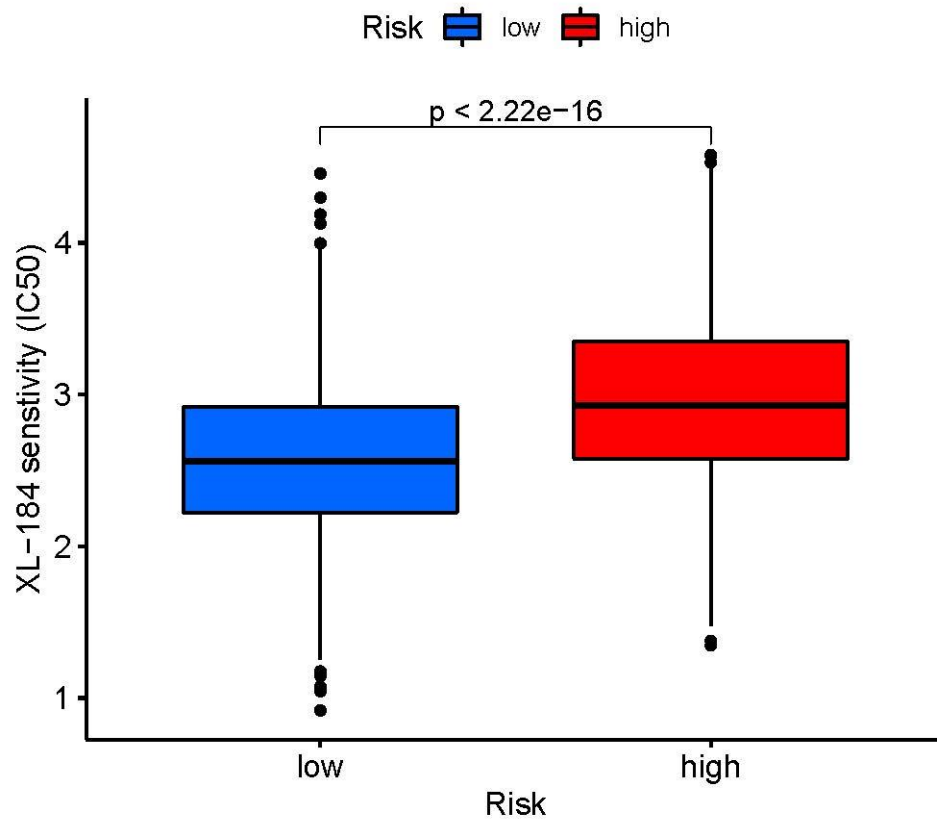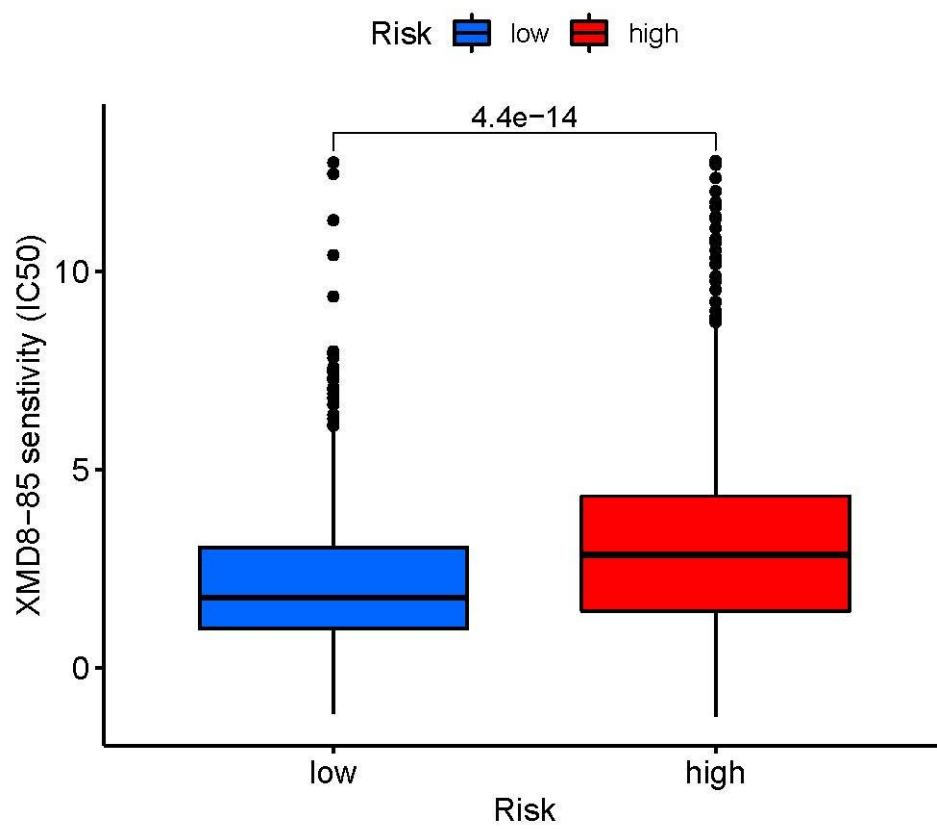

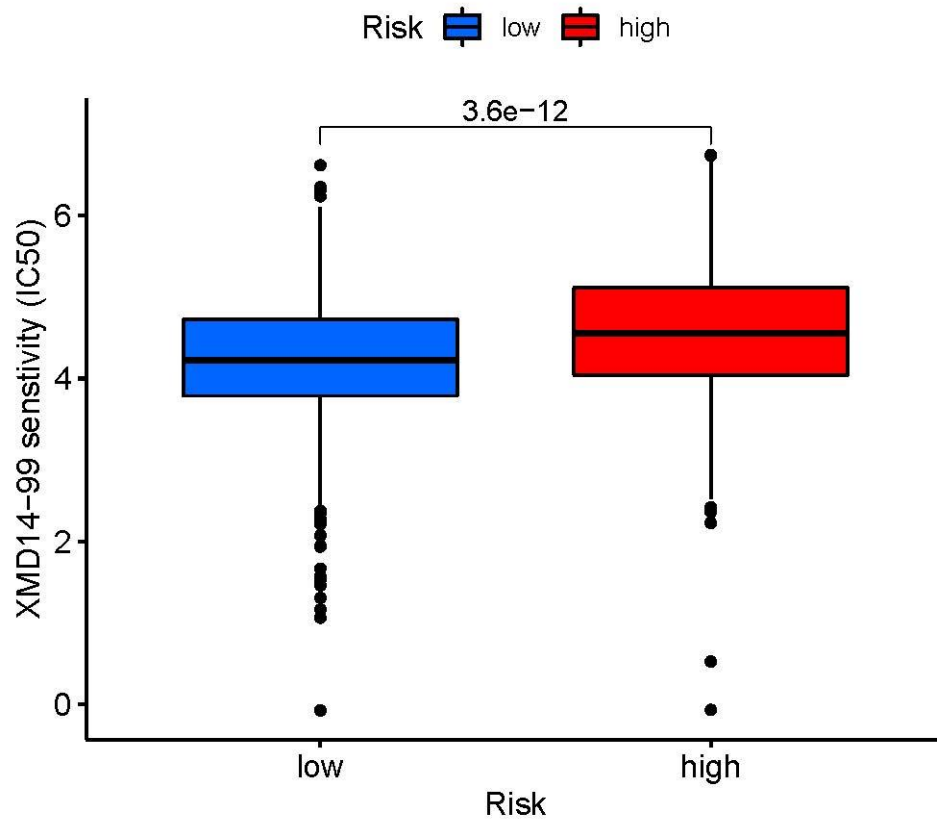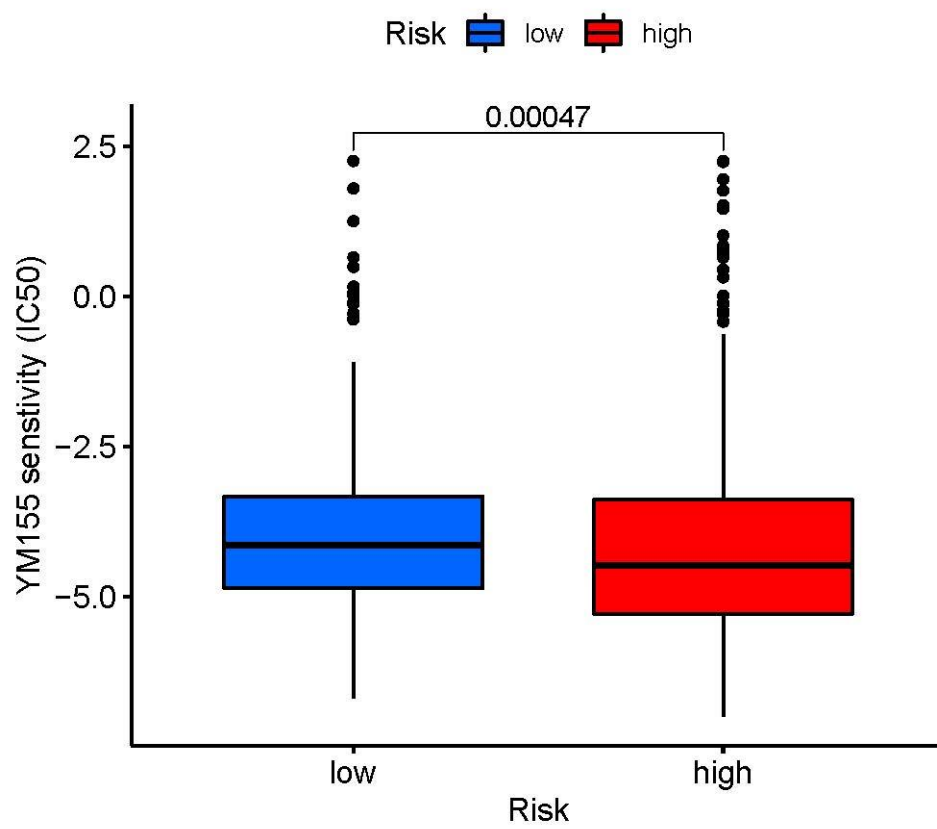

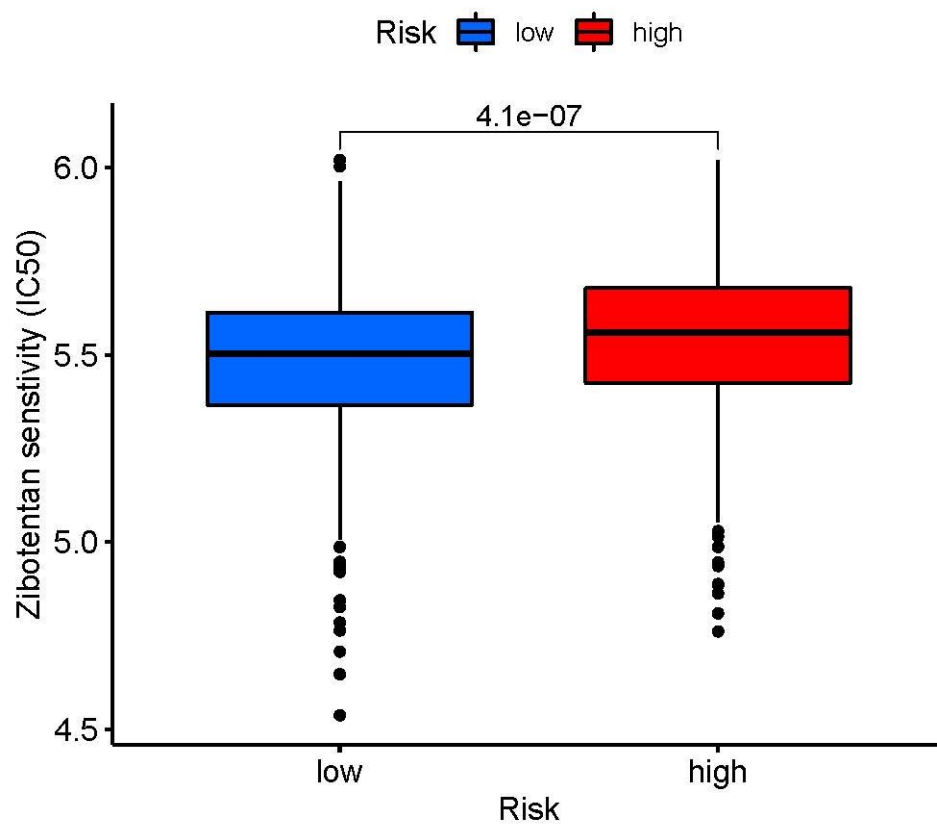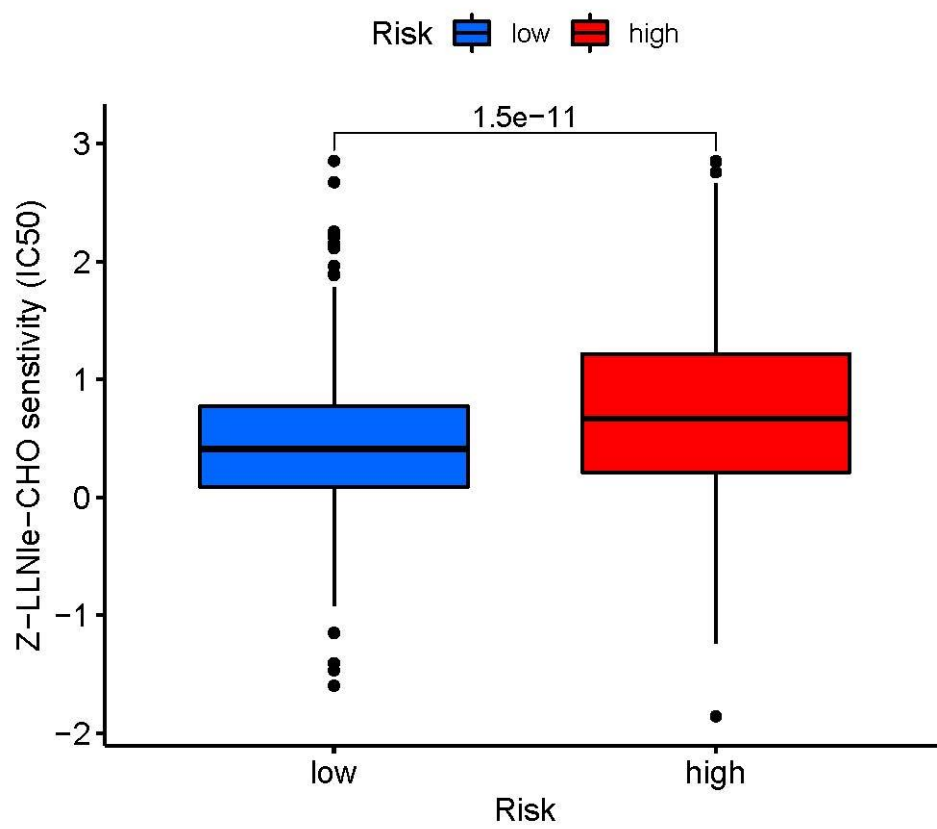

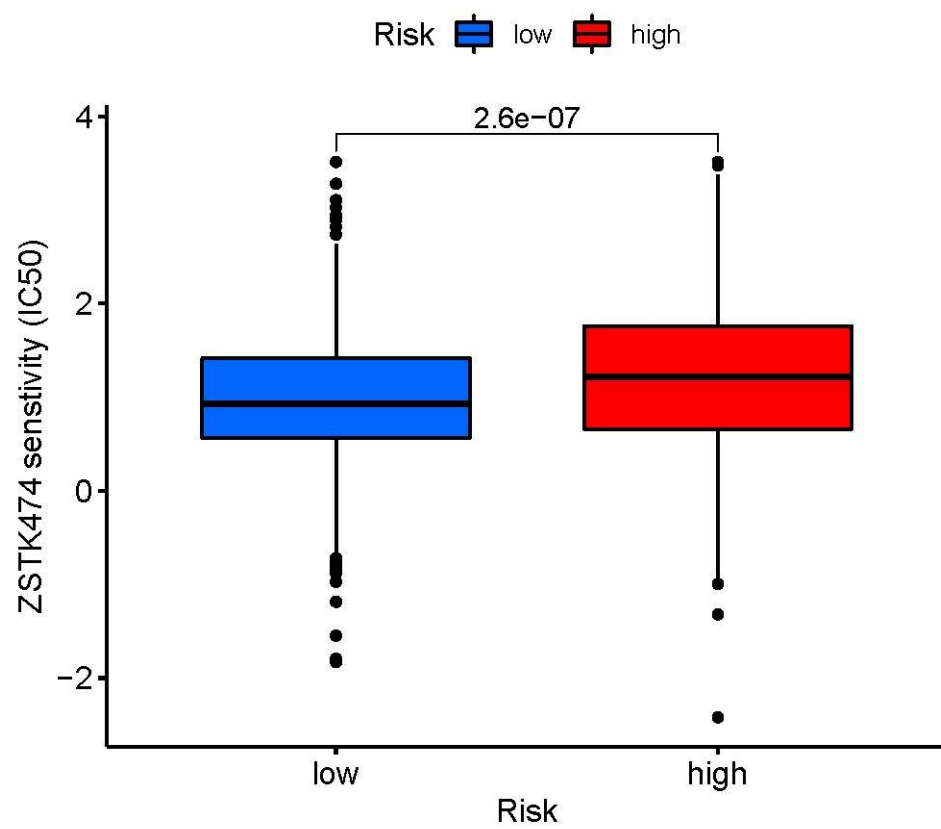

Supplement: Multimedia component 6 — Figure S6 Boxplot of the drugs with the statistical difference in drug sensitivity between the high- and low-risk groups. [file mmc6.pdf]

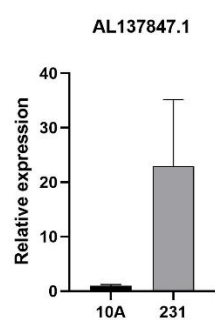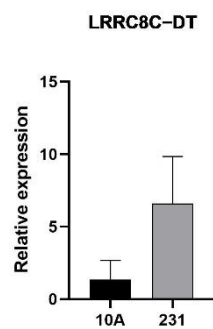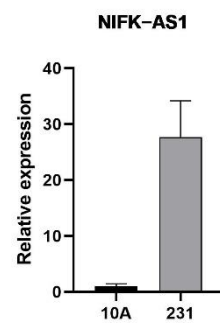

Supplement: Multimedia component 7 — Figure S7 The expression levels of AL137847.1, LRRC8C−DT, and NIFK−AS1. [file mmc7.pdf]
