# Supplementary material for: Prognosis prediction and immune microenvironment features of breast cancer indicated by a cuproptosis-associated long non-coding RNA signature
Source: Genes Dis. 2023 Sep 22;11(5):101110. doi: 10.1016/j.gendis.2023.101110 (PMC11177056; doi:10.1016/j.gendis.2023.101110)
Supplement: Multimedia component 8 — Table S1 Materials and methods. [file mmc8.docx]

**Materials and methods**

**Data collection**

The Cancer Genome Atlas (TCGA) database, now the greatest collection of genetic information on cancer, provided the RNA-seq data sets for all samples and their clinical information. Since the database is completely accessible to the public, this study doesn't need ethical clearance. The work complied with the TCGA database specified data access and publish policies and guidelines. The following are the selection criteria: (a) a histological diagnosis of breast cancer (BRCA); (b) searchable gene expression data; and (c) the ability to retrieve the data. Patients with repeated enrollments were not included in the data. By using the "limma" R program, the transcriptome gene expression profile was preconditioned. Gender, age, TNM staging, clinical stage, follow-up, and survival were all considered clinical data. Patients without a survival state or survival time were omitted from the analysis to lessen the possibility of statistical bias.

**Breast cancer (BRCA) cuproptosis-related lncRNAs characterized and assessed for prognostic significance**

19 potential cuproptosis-related genes (CRGs) had corresponding expression levels taken from the TCGA database. lncRNAs that co-expressed with CRGs were observed by "limma" R (the threshold of correlation coefficient was set at 0.4, p <0.001). Univariate Cox regression analysis was used to broaden the reveal for cuproptosis-related lncRNAs and their association with BRCA survival and prognosis. For variable selection and shrinkage during LASSO regression analysis to lessen the possibility of overfitting, the "glmnet" R package was utilized. The cuproptosis-related lncRNAs participating in multivariate Cox regression analysis were filtered, and the penalty parameter of the model was generated by ten-fold cross-validation pursuant to the minimum criterion (λ values corresponding to the minimum partial likelihood bias).

**Establishment and verification of prognostic risk scoring model of** **cuproptosis-related lncRNAs in breast cancer (BRCA)**

The sample risk index was calculated using the output formula from a multivariate Cox regression analysis that was built to uncover the prognostic value of cuproptosis-related lncRNAs. The principal component analysis (PCA) of all genes, cuproptosis-related lncRNAs, CRGs, and model-building lncRNAs was carried out using the R package "scatterplot3d". To display how high-risk and low-risk samples are distributed spatially, scatter plots are created. The survival curves for the respective survival metrics were obtained using the R packages "survival" and "surminer" for comparison of overall survival (OS) and progression-free survival (PFS) between the high and low risk samples. In accordance with gender, age, TNM staging, and clinical staging, a subgroup study of the risk prediction model was carried out. Receiver operating characteristic (ROC) curve analysis was implemented to gauge the model's prediction effectiveness. The independent risk factors influencing the survival prospect of BRCA were eliminated using univariate and multivariate Cox analysis, and a nomogram was created using the "rms" R package. The nomogram's prediction accuracy was expressed via 1-, 3-, and 5-year calibration curves.

**Functional enrichment analysis**

By applying correlation analysis, CRGs pertaining to the expression of copper death lncrnas involved in formulating models can be produced, and correlation heat maps may be created using the "limma," "reshape2," "tidyverse," and "ggplot2" R packs. The difference analysis is performed (|logFC|1), with the false detection rate (FDR) set to 0.05, to distinguish the gene function enrichment between two risk groups. The "clusterProfiler" R package was then used to evaluate the KEGG signaling pathway analysis and the GO functional enrichment study.

**Tumor mutation load (TMB) analysis and immune-related function analysis**

Tumor mutation load (TMB) was computed in high- and low-risk groups using data on mutations of cuproptosis-related lncRNAs that were retrieved from the TCGA database. The variations in TMB expression throughout the two groups were compared using the "ggpubr" and "limma" R programs. After determining the ideal TMB truncation value, the patients were split into groups with high- and low- TMB. The R packages "survival" and "survminer" were used to perform the survival analysis, and survival curves were shown. TMB and patient risk scores were combined for a survival study, and a survival curve was generated. For the purpose of to assess the enrichment of immune-related behaviors among high- and low-risk groups in accordance with the risk prediction model, single sample gene set enrichment analysis (ssGSEA) was carried out on individuals collected from the TCGA database using the "GSVA" R package.

**Drug sensitivity analysis**

The R packages "BiocManager" and "pRRophetic" were used to determine medication sensitivity and choose viable treatments for BRCA.

**Real time-quantitative PCR** **(RT-qPCR)**

In DMEM, which stands for Dulbecco's Modified Eagle Medium, normal mammary epithelial cells and MDA-MB-231 cell lines were cultivated. DMEM contains 10% fetal bovine serum and 1% penicillin/streptomycin. The cells expanded at 5% CO2 and 37 °C. lncRNA was extracted from each batch of cells by a TRIzol kit. lncRNA was reverse transcribed into cDNA adopting a immense capacity cDNA reverse transcription kit. Real-time quantitative PCR (RT-qPCR) was used to find alterations in the cDNA. Shanghai Sangong Bioengineering Co., LTD. developed and manufactured the primers. The PCR amplification process involved predenaturation at 95 °C for 3 min, 40 cycles of denaturation at 95 °C for 3 s and annealing and extension at 60 °C for 30 s. Table 1 contains a list of primer sequences. 2-ΔΔCT was the technique employed to examine the data.

**Statistical Analysis**

R software (version 4.2.2) served to execute all statistical analyses. To differentiate the expression of lncRNAs in tumor and non-tumor tissues, the student-t test was adopted. Descriptive statistics have been implemented to assess the patients' clinical data. For continuous variables, the distribution was shown as mean standard deviation; for categorical variables, it was shown as frequency and ratio. To formulate the model, patients were allocated at random to training and test groups, and the chi-square test (χ2) served to examine the differences in clinical traits. In accordance with the median risk score, patients were divided into two risk groups. The OS between two risk groups has been contrasted utilizing the Kaplan-Meier analysis and the logarithmic rank test. For the purpose of determining independent determinants of OS, univariate and multivariate Cox regression analyses were conducted. The Mann-Whitney test for p-values corrected by Benjamini-Hochberg (BH) was used to discriminate between two groups based on the ssGSEA scores of immune system. All p <0.05 were regard as statistic significant.
