# Supplementary material for: Prognosis prediction and immune microenvironment features of breast cancer indicated by a cuproptosis-associated long non-coding RNA signature
Source: Genes Dis. 2023 Sep 22;11(5):101110. doi: 10.1016/j.gendis.2023.101110 (PMC11177056; doi:10.1016/j.gendis.2023.101110)
Supplement: Multimedia component 9 — Table S2 Forward and reverse sequences of primers. [file mmc9.docx]

Supple Table 2. Forward and reverse sequences of primers

| Primer | Sequence (5′ to 3′) |
| --- | --- |
| AL137847.1-F | AGGCTGCGGGAATACAAAGTGAC |
| AL137847.1-R | CCTCCTCTTAGCAACAGGCACATC |
| NIFK-AS1-F | CCCGAAGGACCCACAGATTTGC |
| NIFK-AS1-R | CTCCCATCCCAACACATACACTAGC |
| LRRC8C-DT-F | CCCGAAGGACCCACAGATTTGC |
| LRRC8C-DT-R | AACGGCTCTCTCAGTGGGATGG |
| GAPDH-F | CAGGAGGCATTGCTGATGAT |
| GAPDH-R | GAAGGCTGGGGCTCATTT |
