# Supplementary material for: Prognosis prediction and immune microenvironment features of breast cancer indicated by a cuproptosis-associated long non-coding RNA signature
Source: Genes Dis. 2023 Sep 22;11(5):101110. doi: 10.1016/j.gendis.2023.101110 (PMC11177056; doi:10.1016/j.gendis.2023.101110)
Supplement: Multimedia component 10 — Table S3 The baseline clinical characteristics of included patients. [file mmc10.docx]

Supple Table 3. The baseline clinical characteristics of included patients

| Covariates | Type | Total | Test | Train | Pvalue |
| --- | --- | --- | --- | --- | --- |
| Age | <=65 | 773(70.72%) | 378(69.23%) | 395(72.21%) | 0.3094 |
|  | >65 | 320(29.28%) | 168(30.77%) | 152(27.79%) |  |
| Gender | FEMALE | 1081(98.9%) | 544(99.63%) | 537(98.17%) | 0.0425 |
|  | MALE | 12(1.1%) | 2(0.37%) | 10(1.83%) |  |
| Stage | Stage I | 182(16.65%) | 90(16.48%) | 92(16.82%) | 0.8465 |
|  | Stage II | 619(56.63%) | 315(57.69%) | 304(55.58%) |  |
|  | Stage III | 248(22.69%) | 119(21.79%) | 129(23.58%) |  |
|  | Stage IV | 20(1.83%) | 9(1.65%) | 11(2.01%) |  |
|  | unknow | 24(2.2%) | 13(2.38%) | 11(2.01%) |  |
| T | T1 | 280(25.62%) | 134(24.54%) | 146(26.69%) | 0.5415 |
|  | T2 | 633(57.91%) | 327(59.89%) | 306(55.94%) |  |
|  | T3 | 138(12.63%) | 64(11.72%) | 74(13.53%) |  |
|  | T4 | 39(3.57%) | 18(3.3%) | 21(3.84%) |  |
|  | unknow | 3(0.27%) | 3(0.55%) | 0(0%) |  |
| M | M0 | 909(83.17%) | 456(83.52%) | 453(82.82%) | 0.8252 |
|  | M1 | 22(2.01%) | 10(1.83%) | 12(2.19%) |  |
|  | unknow | 162(14.82%) | 80(14.65%) | 82(14.99%) |  |
| N | N0 | 515(47.12%) | 272(49.82%) | 243(44.42%) | 0.2827 |
|  | N1 | 361(33.03%) | 171(31.32%) | 190(34.73%) |  |
|  | N2 | 120(10.98%) | 58(10.62%) | 62(11.33%) |  |
|  | N3 | 77(7.04%) | 34(6.23%) | 43(7.86%) |  |
|  | unknow | 20(1.83%) | 11(2.01%) | 9(1.65%) |  |
