# Supplementary material for: Prognosis prediction and immune microenvironment features of breast cancer indicated by a cuproptosis-associated long non-coding RNA signature
Source: Genes Dis. 2023 Sep 22;11(5):101110. doi: 10.1016/j.gendis.2023.101110 (PMC11177056; doi:10.1016/j.gendis.2023.101110)
Supplement: Multimedia component 11 [file mmc11.docx]

In 95% of the world's nations, breast cancer (BRCA) ranks either first or second among the causes of cancer death in women, which claims more disability-adjusted life years (DALYs) for women globally than any other malignancy. Cuproptosis, independently induced by copper ions and unaffected by other inhibitors of programmed death known, was found by Tsvetkov et al. last year. The buildup of copper in cells is the primary factor in this mechanism of programmed cellular death. Cuproptosis is brought on by an excess of intracellular copper's direct binding to the TCA cycle components in mitochondrial respiration, which results in the buildup of lipid acylation proteins and the loss of iron-sulfur cluster proteins. Additionally, FDX1 controls the upstream process of protein lipid acylation. Many lncRNAs with high stability, including PCA3, PCGEM1, and PCAT-1, can be found in the plasma or urine of tumor patients, and their levels can be utilized as indicators of disease severity. Cuproptosis-related lncRNAs have thus been researched and used to forecast the prognosis of cancers including osteosarcoma, gastric cancer, kidney cancer, liver cancer, lung cancer, and others. This paper is shooting to create the corresponding predictive risk score model, thoroughly investigate the molecular signature and clinical connection of cuproptosis-related lncRNAs in breast cancer (BRCA), and identify the patients sensitive to immune checkpoint drugs, in order to advance early identification and intervention, which is the therapeutic goal.

In this case study, 7 cuproptosis-related lncRNAs were sniffed out by association analysis, and the lncRNAs and clinical data of 1113 patients of breast cancer (BRCA) were retrieved from the TCGA database. LASSO regression was used to perform a second screening, leaving 3 lncRNAs behind (AL137847.1, LRRC8CDT, and NIFKAS1), of which, AL137847.1 has a positive regulatory interaction with LIAS, while PDHA1 has a negative regulatory link as following **Figure 1**.


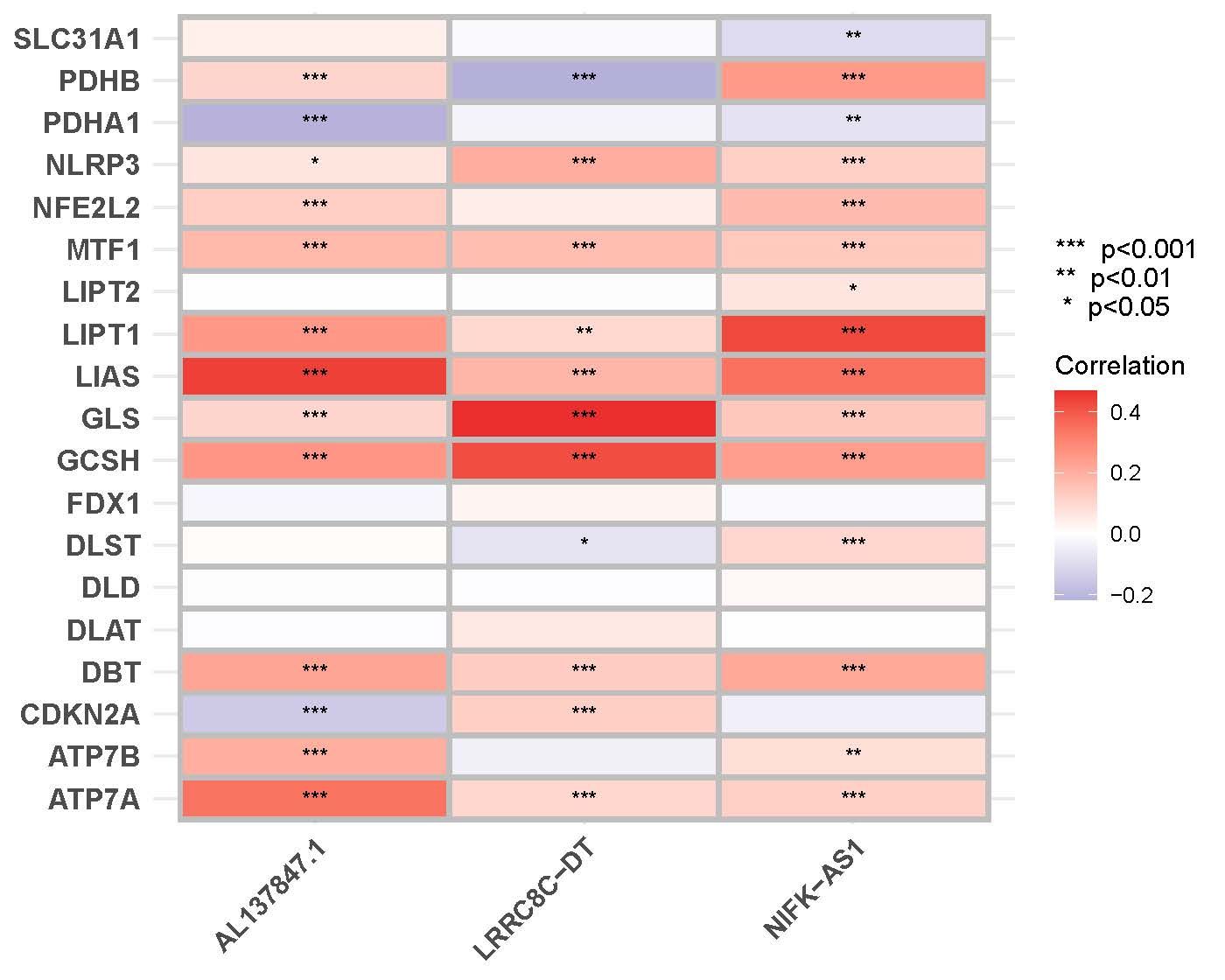


**Figure 1.** Heat map of multivariate analysis of correlation between lncRNAs and copper poisoning related genes involved in model construction.

Multivariate Cox regression analysis was adopted to formulate the prophetic risk model, which gave rise to the subsequent formula for risk scoring: risk score =∑(Coefi * Expi). With the median risk score as the border, we separated the samples into high- and low-risk groups. The groups with high and low risk were constrasted in the light of overall survival (OS) and progression-free survival (PFS), the results showed that PFS is distinct because it was higher in the low-risk group's first 10 years and tends to be the same or lower after (p <0.05), but OS in the high-risk population is obviously lower than that of the low-risk group in the overall, training, and experimental groups as following **Figure 2 and Figure 3**.


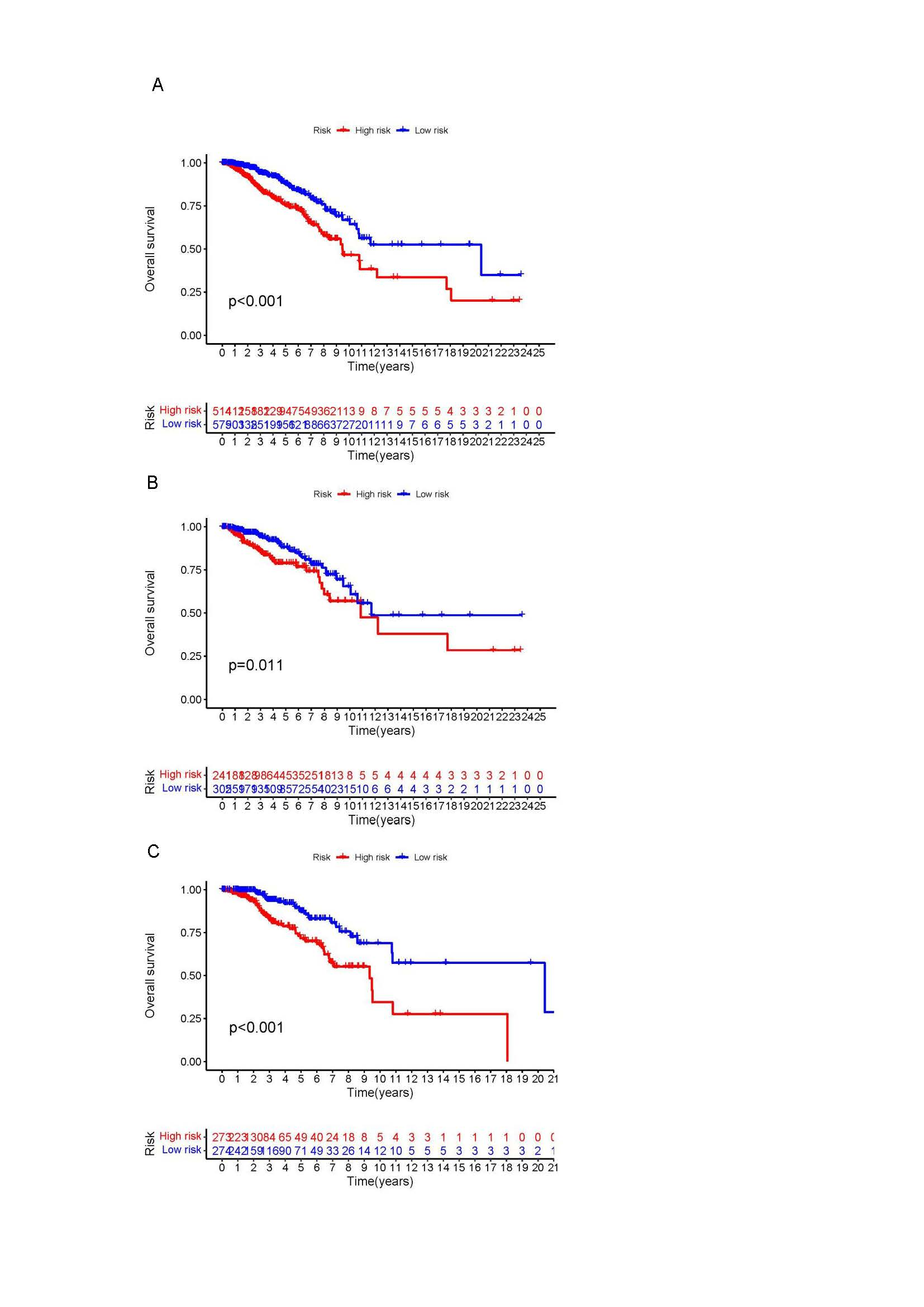


**Figure 2.** The survival curve of (A) overall, (B) the text group, (C) the train group.


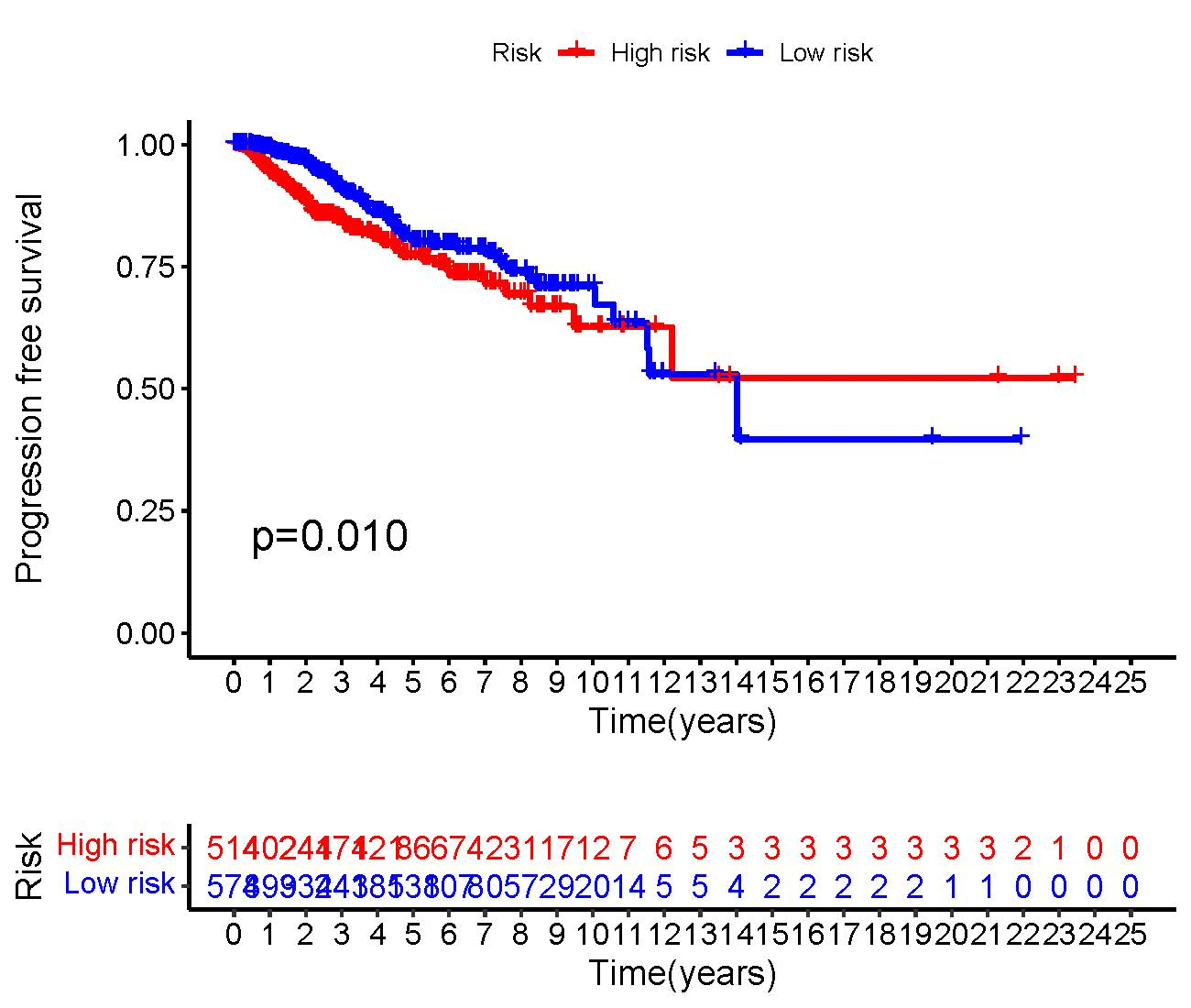


**Figure 3.** Overall progression free survival (PFS).

The AUC value, assessed by the Receiver Operator Characteristic Curve (ROC), turned out 0.763 in 1 year, 0.639 in 3 years and 0.608 in 5 years (**Figure 4**).


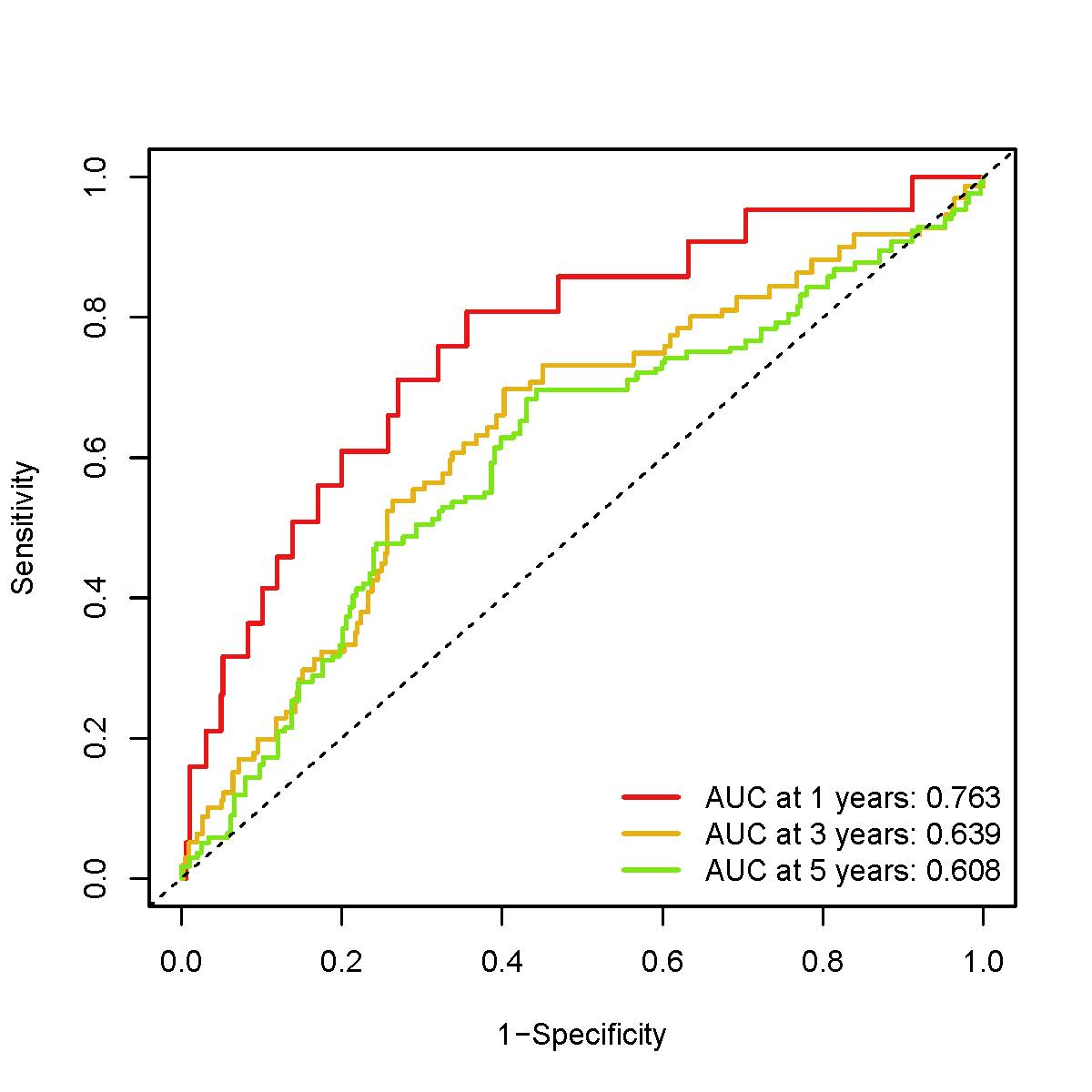


**Figure 4.** Receiver operator characteristic (ROC) curve of prognostic model.

According to PCA analysis, as illustrated in Figure 5, the risk prognostic model can effectively distinguish between groups with high and low risk.


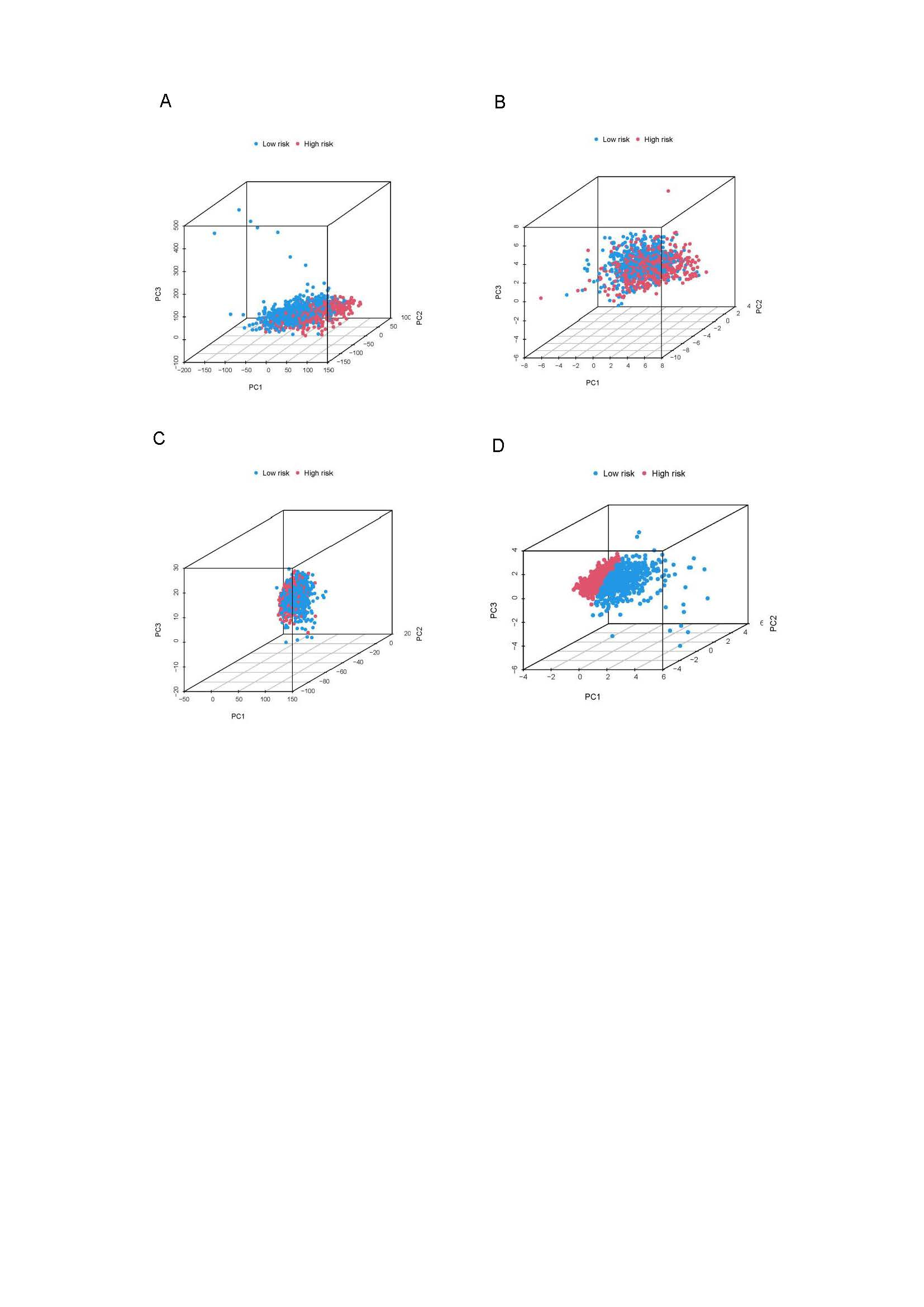


**Figure 5.** Principal component analysis (PCA) analysis of (A) overall genes in TCGA cohort, (B) the cuproptosis-related genes, (C) the cuproptosis-related lncRNAs, (D) the lncRNAs included in prognostic model.

**Figure 6** displays the outcomes of a univariate Cox regression analysis, which revealed that the risk score (HR=1.558, 95%CI 1.289 to 1.882, P<0.001), age (HR=1.033, 95%CI 1.019 to 1.048, P<0.001), clinical stage (HR=2.141, 95%CI 1.695 to 2.705, P<0.001) and TNM staging (HR=1.546, 95%CI 1.248 to 1.916, P<0.001; HR=1.668, 95%CI 1.391 to 2.001, P<0.001; HR=6.567, 95%CI 2.687 to 11.694, P<0.001) were risk factors for prognosis of BRCA. After controlling for other confounding variables, multivariate Cox analysis revealed that risk scores (HR=1.523, 95% CI 1.249 to 1.857, P<0.001) continued to be an independent predictive predictor for the prognosis of BRCA.


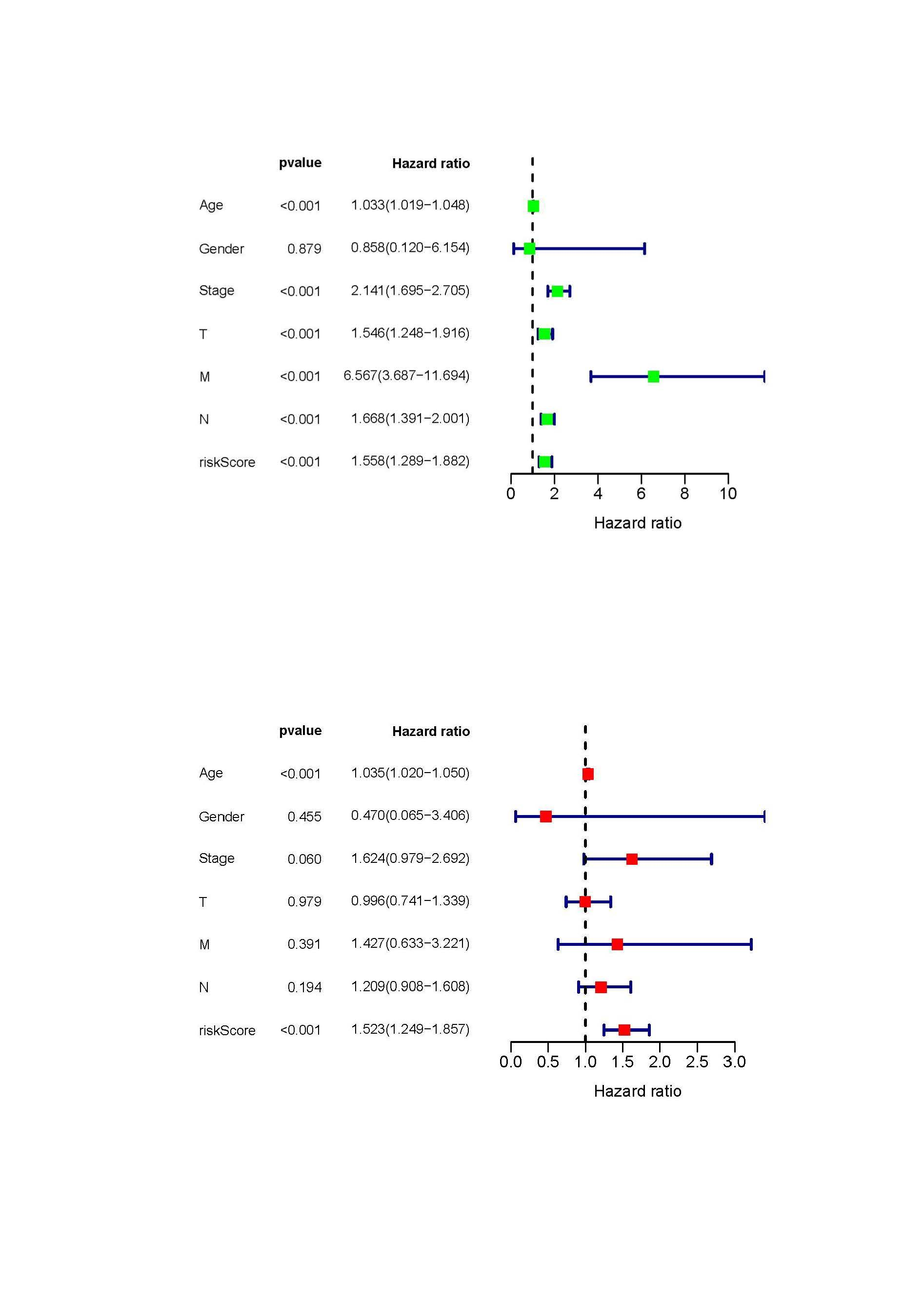


**Figure 6.** Univariate independent prognostic analysis.

According to **Figure 7**, cuproptosis-related lncRNAs were accurate at predicting the prognostic risk model of BRCA, as evidenced by the risk score model's AUC value of 0.763, which was greater than any other clinical risk factors and second only to age (0.823).


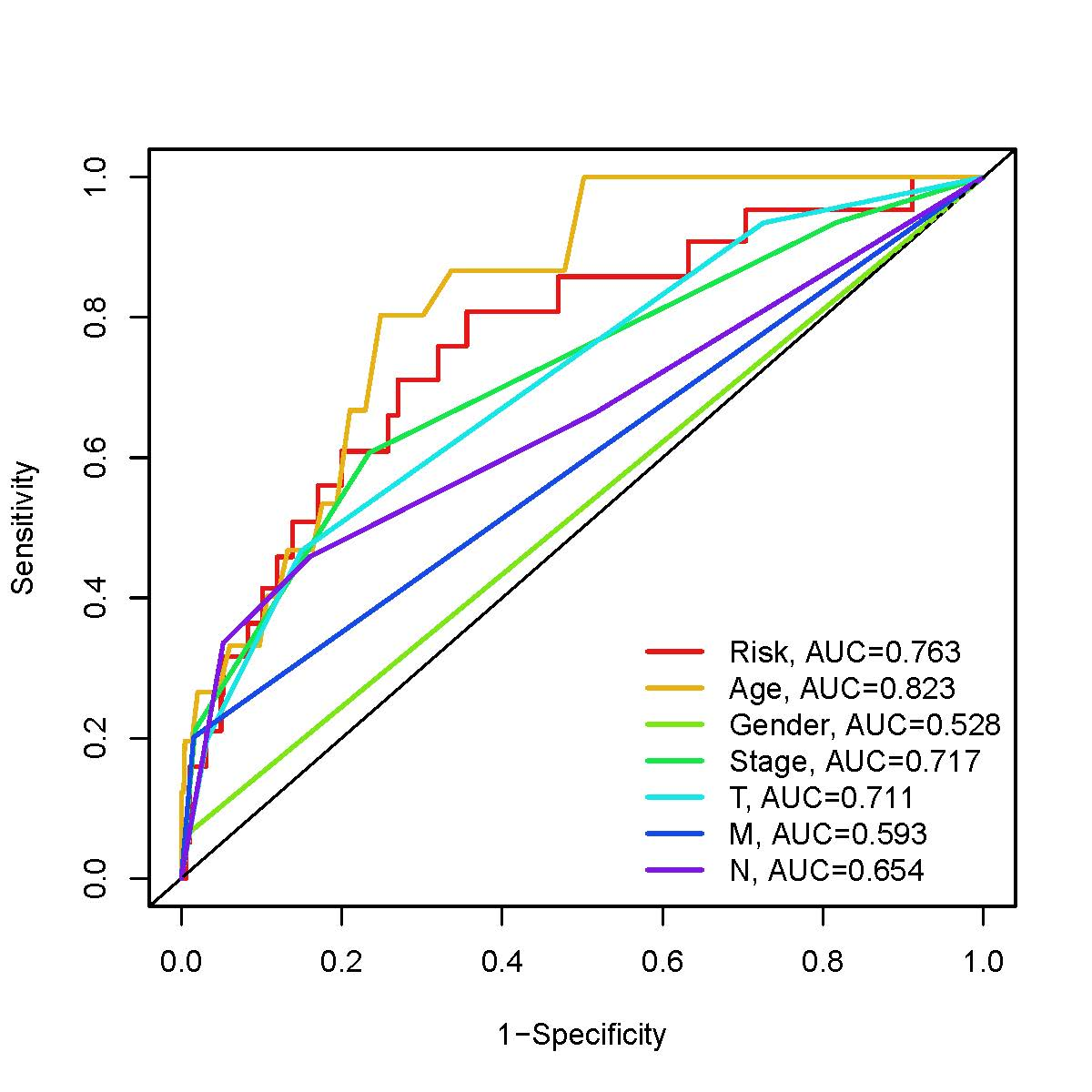


**Figure 7.** Receiver operator characteristic (ROC) curve of all risk factors.

When applied to female patients over the age of 40 with any clinical stage and any TNM stage, the risk score model performed well (p<0.05), according to the subgroup analysis of the prediction model.

A nomogram was created, as seen in **Figure 8**, on the basis of the risk scores model and clinical traits to forecast the likelihood of 1-year, 3-year, and 5-year OS for the TCGA training dataset. According to calibration curves of 1-, 3-, and 5-year OS probability outcomes, predicted and actual survival are highly correlated. These findings imply that the expression of these three lncRNAs, AL137847.1, LRRC8C−DT, and NIFK−AS1, is a valid predictor of prognosis in BRCA patients.


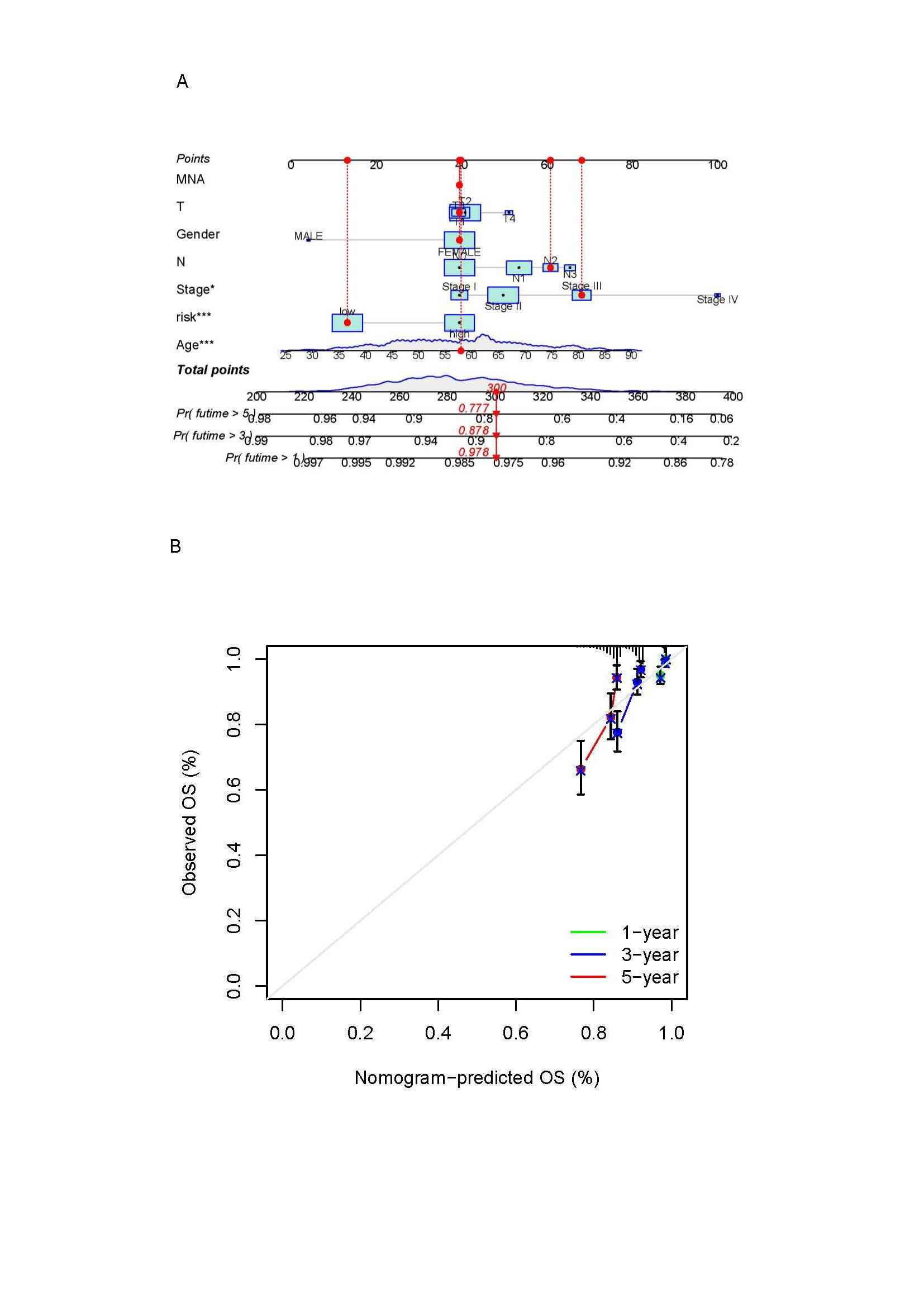


**Figure 8.** (A) Nomogram of overall survival (OS) prognosis. (B) Calibrated curves of the nomogram.

Three elements made up the GO functional enrichment analysis: biological process (BP), cellular component (CC), and molecular function (MF). According to BP, **Figure 9** demonstrates that cuproptosis-related lncRNAs were primarily enriched in leukocyte migration, muscular system process, and epithelial cell proliferation. In MF, lncRNAs were enriched in signal receptor activation activity, receptor ligand activity, DNA-binding transcription activator activity and RNA polymerase II-specific DNA-binding transcription activator activity. For CC, lncRNAs were mainly concentrated in collagen−containing extracellular matrix.


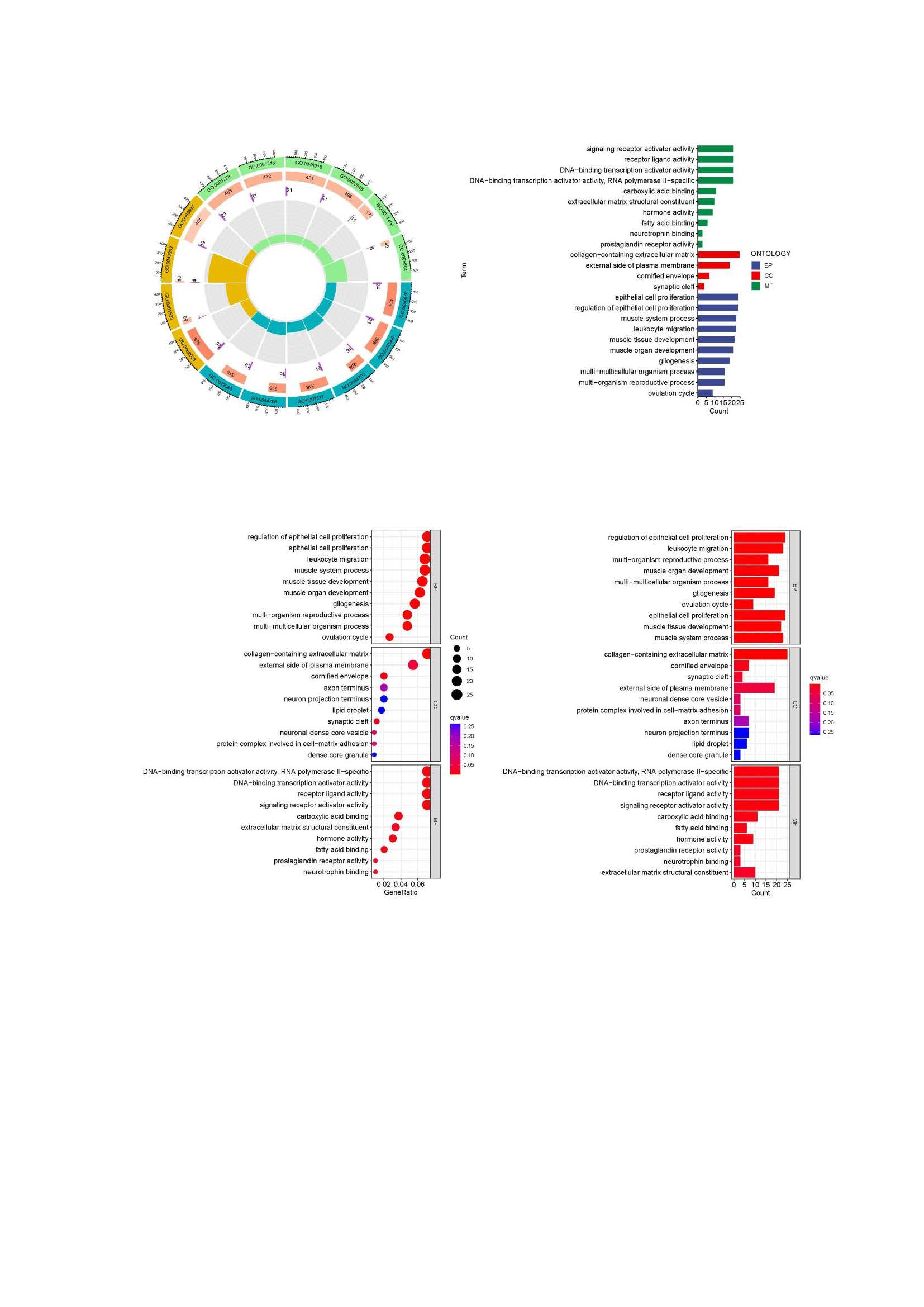


**Figure 9.** Results of GO analyses in the TCGA cohort.

In KEGG pathway enrichment analysis, cuproptosis-related lncRNAs were mainly related to neuroactive ligand-receptor interactions, as shown in **Figure 10**.


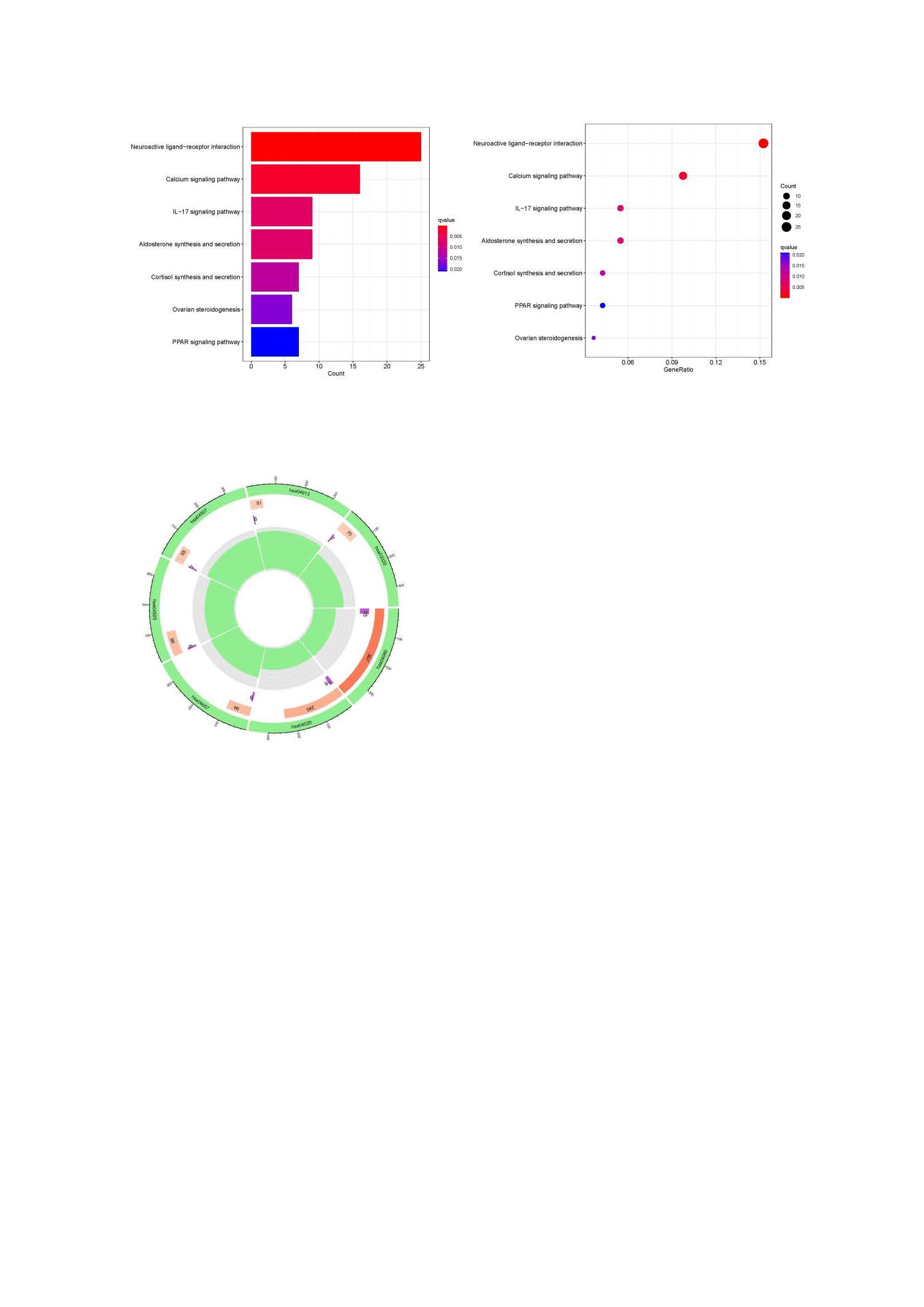


**Figure 10.** Results of KEGG analyses in the TCGA cohort.

In order to compare patients in two risk groups for mutations, mutation data were acquired from the TCGA, among which TP53 and TTN had the highest rate of mutation. TMB varied across high and low risk groups and the prognosis for high TMB was poor as **Figure 11**. In contrast to patients in other groups, those in the group with a high TMB in conjunction with a high risk had the worst outcome (p<0.05).


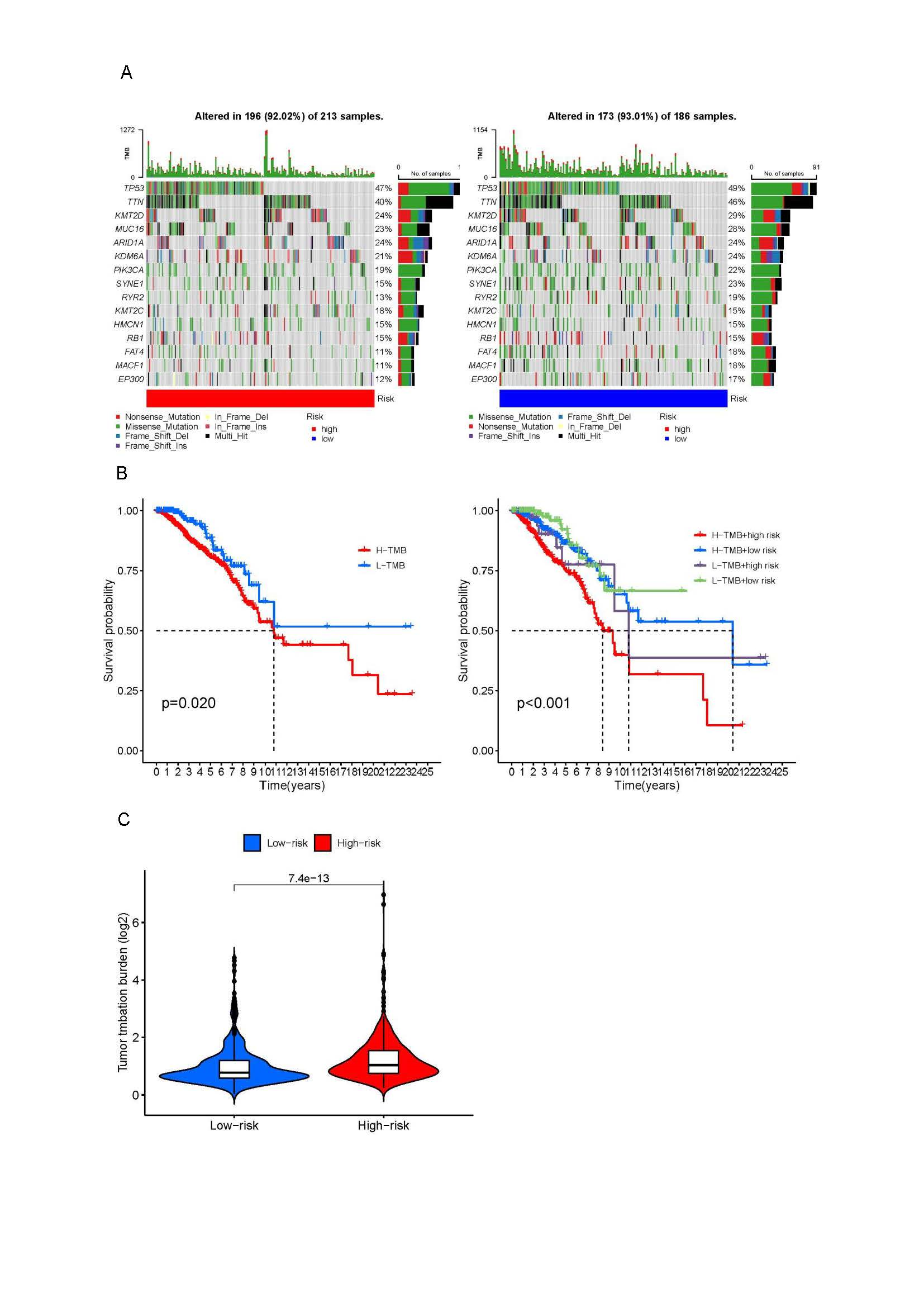


**Figure 11.** (A) The hierarchical clustering heat diagram of ssGSEA enrichment score. (B) Survival analysis of TMB. (C) Violin diagram of the variation analysis of tumor mutational burden (TMB).

The ssGSEA enrichment score reflects the level of immune-related function activity in samples from high- and low-risk groups, and the results are displayed in the hierarchical clustering heat diagram **(Figure 12)**. The graph demonstrates a substantial difference in type II IFN response and APC co-stimulation between two risk group (p<0.05).


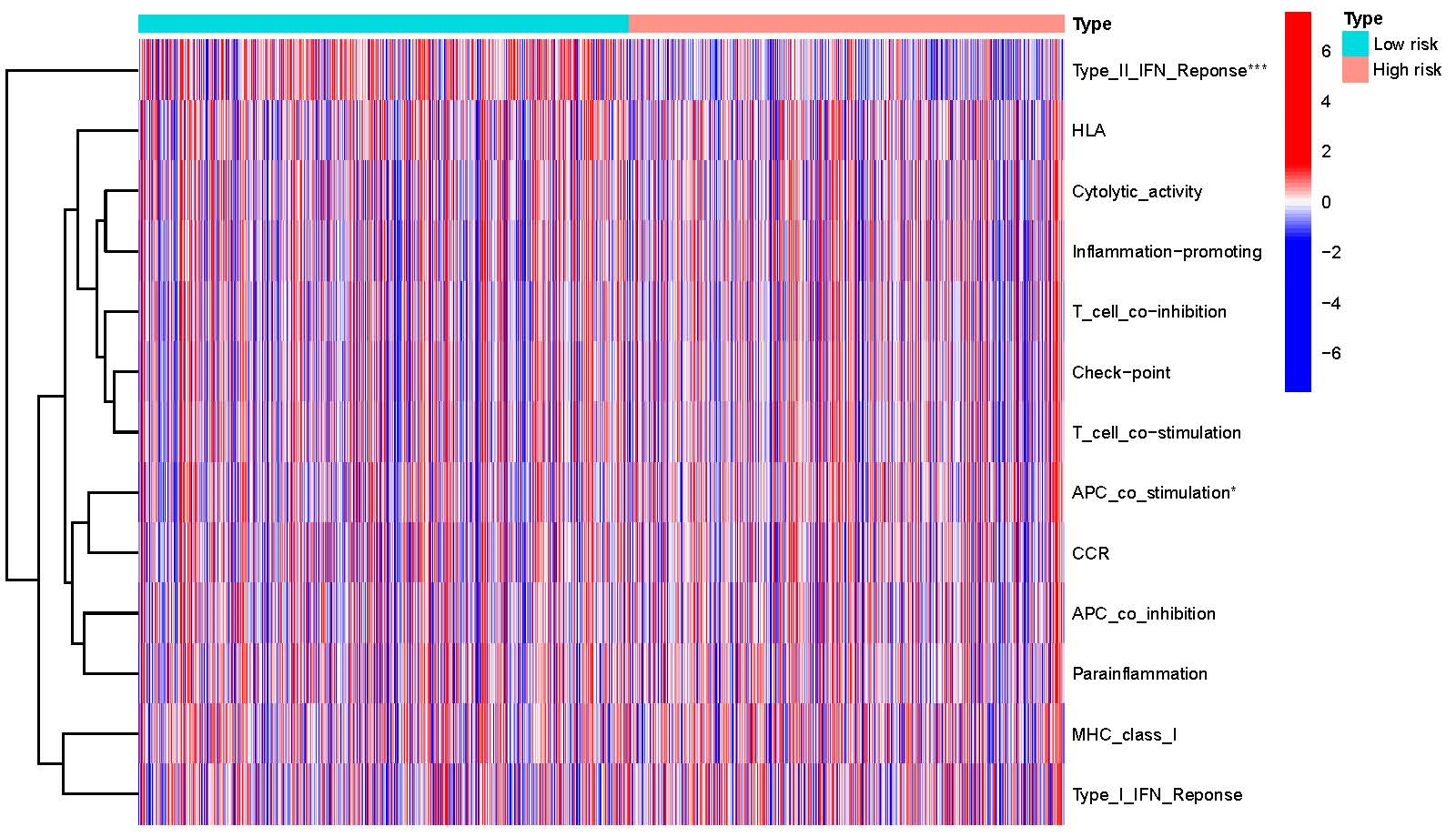


**Figure 12.** Heatmap of immune function.

The expression level of AL137847.1, LRRC8C−DT, and NIFK−AS1 were notably higher in MDA-MB-231 cell than in normal mammary epithelial cells in **Figure 13 to 15**.


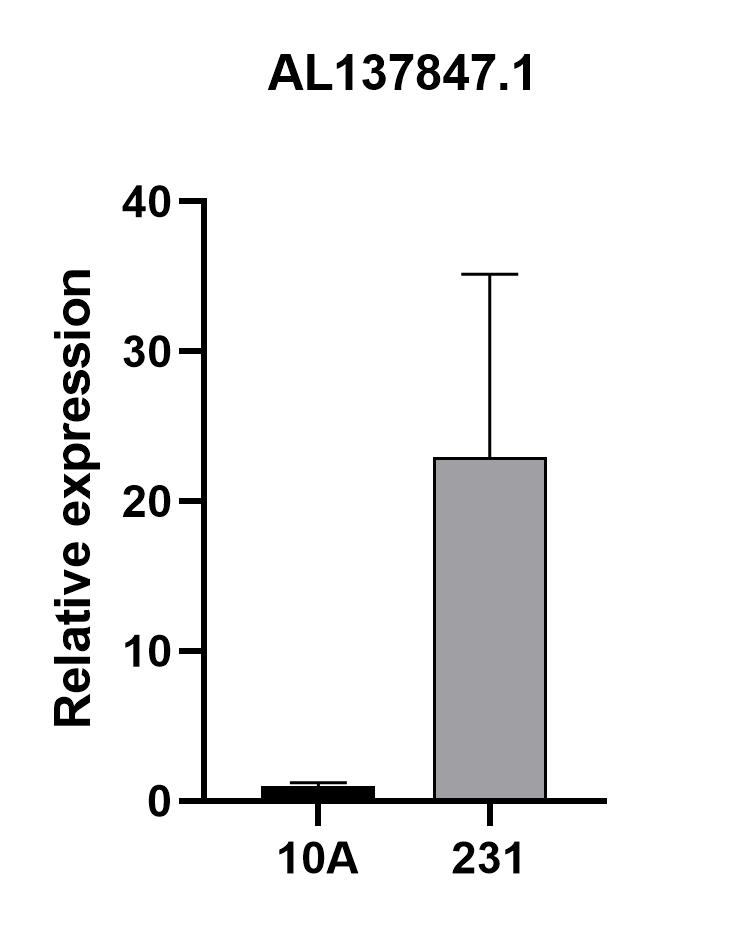


**Figure 13.** The expression level of AL137847.1.


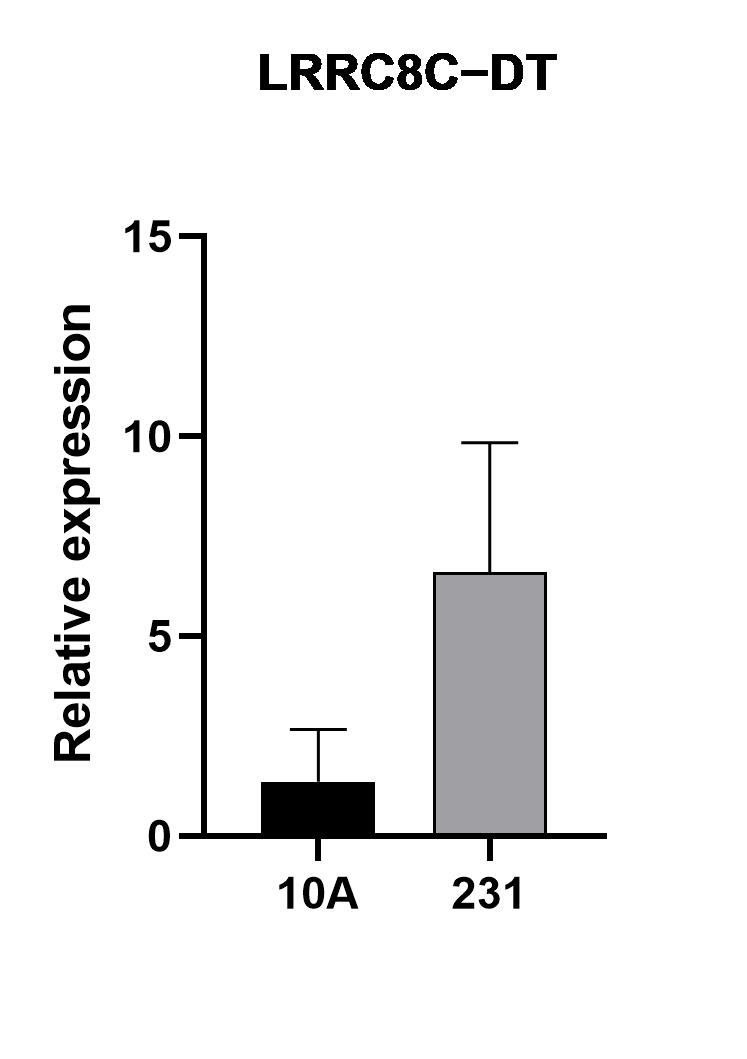


**Figure 14.** The expression level of LRRC8C−DT.


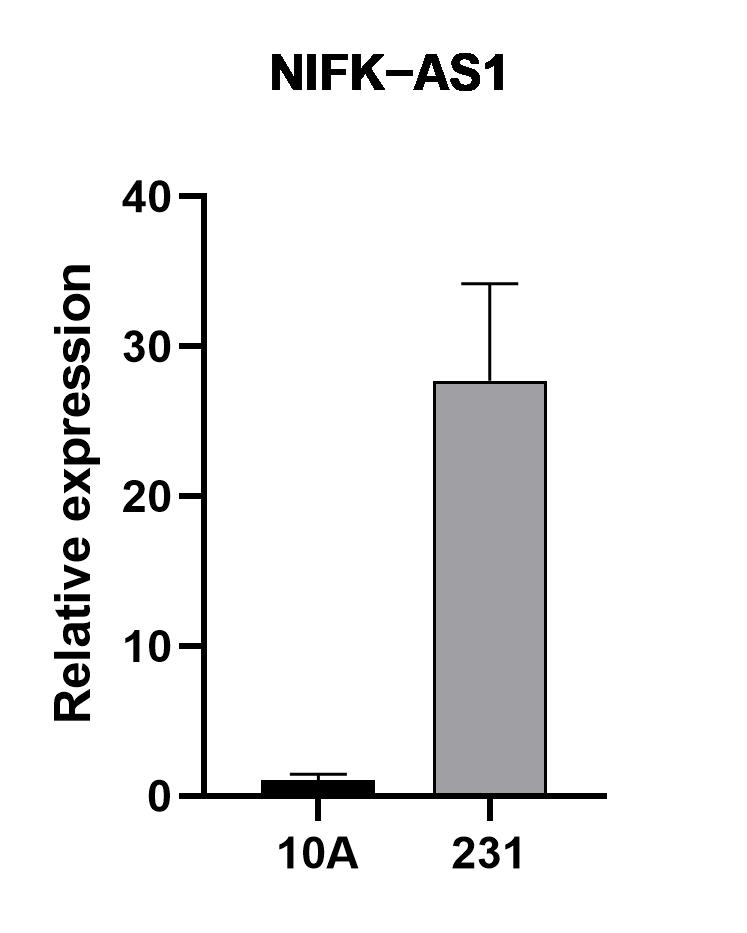


**Figure 15.** The expression level of NIFK−AS1.

Boxplot was drawn to predict drug sensitivity, A−770041, AC220, AP−24534, AS601245, AS605240, and BAY 61−3606, BI−2536, BMS−509744, BMS345541, BX−912, CAL−101, CGP−082996, CGP−60474, CMK, CP724714, DMOG, FH535, FMK, FR−180204, GSK107091 6, GSK1904529A, GW−2580, GW843682X, IPA−3, JW−7−52−1, KIN001−102, KIN001−135, MS−275, NG−25, NPK76−II−72−1, NSC−87877, OSU−03012, QL− XII−47, PHA−665752, PF−562271, S-triphenylmethyl-L-cysteine, TAE684, TGX221, TL−2−105, VX−11e, VX−680, WZ−1−84, XMD8−85, XMD14−99, XL−184, YM155, Z−L LNle−CHO, ZSTK474, Ebomycin B, Obakra mesulfonate, siterpene lactone, Bexarotene, pheneformin, Bleomycin, Dasatinib, toxic carotene, anthracene, fluorouracil, nuclear inhibitor, cyclopamine, gemcitabine, Genentech Cpd 10. Lapatinib, rapamycin, lincetinib, rusotinib, middoxoline, pazopanib, Zipotentam, Secatinib, Sunitinib, bryostatin 1, Tipirfanil, pyrimidine, Ispin mesulate, shiverin, and paclitaxel were significantly different between two risk groups. Patients in low-risk group were more susceptible to AS605240, CP724714, FH535, GW−2580, MS−275, YM155 and pyrimethamine. In high-risk group were more sensitive to others.

The research has an array of implications. In the beginning, we set out to create an algorithm for predicting for breast cancer (BRCA) based on the potency of curopotosis-related lncRNA, which may result in novel remedies for BRCA. Our study also took into account overall survival (OS) simply, progression-free survival (PFS), risk score, immune-related function analysis, immune escape score, potential drug screening for medical conditions, and various additional variables as opposed to conventional prognostic models that only focus on overall survival (OS), which is the primary outcome. More specifically, for individuals with advanced cancer, an improvement in PFS may be accompanied by a decrease in tumor burden and symptom alleviation. To estimate survival time and investigate risk factors, patients were spanided into high- and low- risk groups. The TIDE score and TMB analysis can help to provide guidance for immunotherapy strategy.
